# Characterization of RufT Thioesterase Domain Reveals Insights into Rufomycin Cyclization and the Biosynthetic Origin of Rufomyazine

**DOI:** 10.1021/acschembio.4c00802

**Published:** 2025-03-06

**Authors:** Yaoyu Ding, Gustavo Perez-Ortiz, Alexandra-Georgiana Butulan, Hamzah Sharif, Sarah M. Barry

**Affiliations:** Department of Chemistry, Faculty of Natural, Mathematical and Engineering Sciences, King’s College London, Britannia House, 7 Trinity Street, London SE1 1DB, U.K.

## Abstract

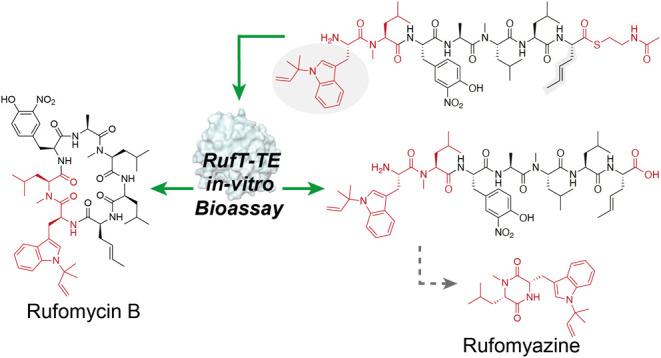

The nonribosomal cyclic peptides (NRcPs) rufomycins, produced by *Streptomyces atratus*, have attracted attention as antimycobacterials. Thus, there has been interest in engineering the corresponding biosynthetic pathway to produce novel derivatives. We have thus investigated the type I thioesterase (TE) of the NRPS RufT that catalyzes rufomycin peptide macrocyclization to understand its tolerance to changes in substrate peptide sequence. In contrast to our previously reported efficient cyclization chemistry, the recombinant RufT-TE domain and RufT-PCP-TE didomain, while tolerating some substrate structural changes, both produce high levels of hydrolyzed peptide. Closer analysis led to the identification of the natural product diketopiperazine rufomyazine in assays. The data indicate, with significant implications for rufomycin production, that RufT produces both cyclic and linear peptides. We propose that rufomyazine forms non-enzymatically from the linear peptide. In addition, it provides evidence for TE domains as gatekeepers in NRPS biosynthesis.

Nonribosomal cyclic peptides (NRcPs) are a family of structurally complex microbial natural products that have historically been a reliable source of antibiotics (*e.g.*, colistin, vancomycin, and daptomycin). Nonribosomal peptide biosynthesis employs the modular multidomain enzymes, nonribosomal peptide synthetases (NRPSs), that act as assembly lines in which each module is responsible for sequentially adding a new amino acid to the covalently bound linear peptide.^1,2^ The staggering structural diversity of NR peptides is due to the ability of NRPS enzymes to utilize non-proteinogenic amino acids and incorporate modifications during peptide elongation, *e.g.*, *N*-methylation, epimerization, and oxidation.^3^ Chemically challenging macrocyclization is a crucial biosynthetic step, typically catalyzed by a C-terminal type I thioesterase (TE) domain on the NRPS and can be head-to-tail or branched, resulting in amide or ester bonds.^2^ Peptide cyclization confers greater resistance to proteases and restricts peptide conformation to provide a more defined surface to interact with a target.^4,5^

Despite their importance to NR peptide biosynthesis, there has been limited biochemical characterization of TE domains, while the dynamic nature of TE domains has created challenges gathering structural data on substrate–protein interactions.^6−9^ Thus, we have little understanding of what determines TE activity and promiscuity. This issue has restricted the potential of TE domains as peptide cyclization biocatalysts as well as the rational engineering of these enzymes to facilitate reprogramming of NRPS pathways to enable, for example, the production of antibiotic derivatives.^10^ In addition, while the chemical synthesis of linear peptides is straightforward, regio- and chemoselective peptide macrocyclization remains a significant synthetic challenge,^11^ exacerbated by the structural complexity of natural products.

We recently developed a chemical peptide cyclization method inspired by NRPS modular logic and thioesterase-catalyzed cyclization (Figure 1D).^12^ The success of our chemistry relies on using a biosynthetically defined peptide sequence. In doing so, we could cyclize peptides in minutes, indeed, more rapidly than previously characterized TE domains. We developed the chemistry using rufomycins^13^ (also known as ilamycins), a family of nonribosomal peptides produced by several strains of the bacterium *Streptomyces atratus*(14−17) with anticancer^18^ and importantly antimycobacterial activity^19,20^ (Figure 1A). The rapidity and success of the chemistry led us to study the NRPS TE domain responsible for rufomycin cyclization, RufT-TE.^14,17,21^ Type I TE domains typically catalyze cyclization between termini with l and d configuration. However, rufomycin and cyclomarin TE domains are the only two known to catalyze macrolactamization between two amino acids with homo configuration, a reaction reported to be chemically disfavored.^9^ Our goal was to elucidate the biochemical activity, efficiency, and substrate tolerance of the unusual thioesterase RufT-TE and to compare these data to the results of our chemical cyclization of the same sequences (Figure 1D).^12^

**Figure 1:**
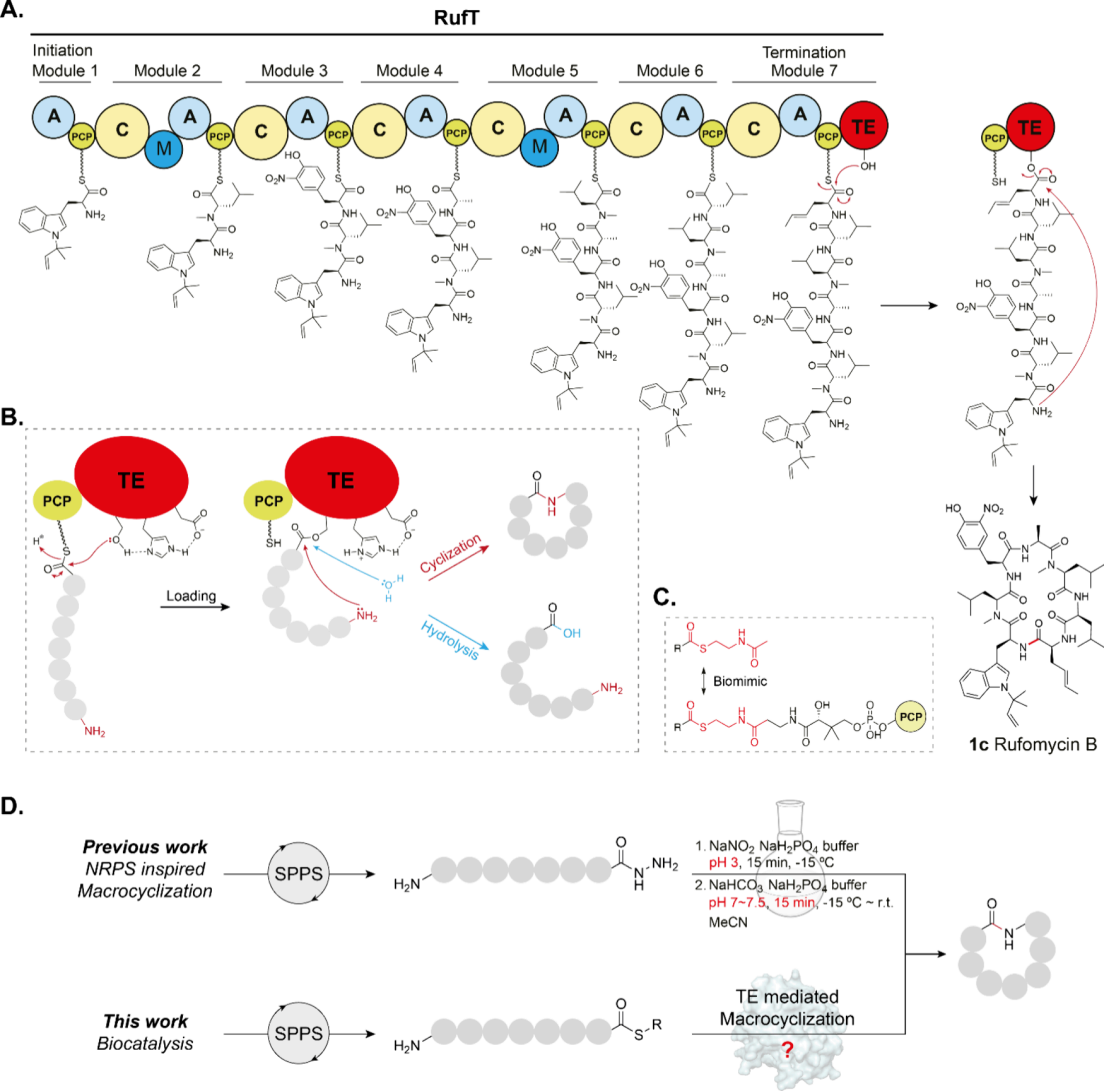
**Biosynthesis of rufomycins and exploitation of NRPS enzymes for NRcP diversification strategies.** Gray circles represent amino acids. (A) NRPS-directed biosynthesis of rufomycins and Type I TE catalytic mechanism. Rufomycin B is biosynthesized by NRPS RufT. Circles in RufT represent individual domains. A = adenylation domain, which selects and loads amino acids onto the PCP; PCP domain = peptidyl carrier protein modified by phosphopantetheine (pPant); C = condensation domain, which catalyzes amide bond formation; M = methyltransferase, which catalyzes SAM-dependent *N*-methylation; TE = thioesterase. (B) TE-domain-catalyzed peptide cyclization or hydrolysis employing catalytic triad (Ser-His-Asp). (C) *N*-Acetylcysteamine thioesters can act as pPant mimics in *in vitro* thioesterase assays. (D) (top) Our previous work inspired by NRPS enzymology and assembly line logic led to a method to generate natural product cyclic peptides and understand the order of NRPS modules, producing rufomycin derivatives with 65–90% conversion. (bottom) This work investigates the complementary approach of utilizing TE domains as peptide macrocyclization biocatalysts *in vitro*.

To characterize RufT-TE, we required a robust synthesis of the linear rufomycin precursor peptide. Previous studies have shown that peptides derivatized with *N*-acetylcysteamine (NAC) can be used as truncated mimics of phosphopantetheinyl (Ppant)-bound peptides and are often sufficient for recognition by recombinant TE domains (Figure 1C).^23,24^ During NRPS peptide elongation, the peptide is covalently bound via a Ppant arm to a peptidyl carrier protein (PCP) (also known as a thiolation domain)^25^ and is transferred for macrocyclization to a catalytic serine in the TE domain to form an acyl enzyme intermediate (Figure 1B). *N*-Acetylcysteamine thioester (SNAC) peptides were synthesized using acyl azide intermediates to form thioesters to avoid epimerization (Supplementary Figure 1).^26−28^ The non-proteinogenic amino acids *trans*-l-crotylglycine and *N*-prenyl-l-tryptophan were synthesized as we previously reported.^12,29−31^ The linear rufomycin precursor **1a**, containing a C-terminal acyl hydrazide, was synthesized via Fmoc SPPS and, following cleavage, oxidized to the acyl azide peptide **S1** as previously reported (Figure 2D).^32^ Addition of NAC at low temperature afforded SNAC peptide **1b** cleanly within minutes (Supplementary Figure 1). Derivatives **2b**–**8b** were similarly synthesized and purified by HPLC. In addition, the synthetic cyclic peptide rufomycin B **1c** was prepared as a standard (Figure 1A and Supplementary Figure 2).

A construct encoding *rufT-TE* was designed by aligning the RufT-TE domain amino acid sequence with structurally characterized NRPS TE domains to identify the domain boundary (Supplementary Figure 4). Recombinant His_6_-SUMO-RufT-TE was produced in *Escherichia coli* Tuner DE3 and assayed for macrocyclization with SNAC peptide **1b** (Supplementary Figures 10 and 12 and Figure 2). While His_6_-SUMO-RufT-TE was active as an isolated domain, conversion of **1b** to **1c** was low (4%), with hydrolyzed peptide **1d** a significant side product (20%) (Figure 2 and Supplementary Figure 16). The enzymatically cyclized product was confirmed by comparison to rufomycin B previously isolated from *S. atratus* culture^13^ and chemically synthesized **1c** (Supplementary Figure 2B and 16).^12^ No spontaneously hydrolyzed product was detected in negative controls containing thermally denatured RufT-TE within 3 h (Supplementary Figure 16). Thus, we conclude that the SNAC peptide is recognized but hydrolysis of either the SNAC peptide in the active site or the acyl enzyme intermediate competes significantly with cyclization. Several conditions, including temperature, ionic strength, ratio of substrate and enzyme, BSA, and detergent were screened, but there was no appreciable improvement in conversion or the ratio of **1c** to hydrolyzed product **1d** (Supplementary Figures 14 and 27). These results surprised us, as they are in sharp contrast to the ease with which the corresponding acyl azide peptide cyclizes in our previously reported macrocyclization chemistry (>80% within 15 min) (Supplementary Figure 2).^12^ We hypothesized that the N-terminal SUMO tag, added to improve protein solubility, may affect the enzyme activity given the flexibility of the thioesterase lid region (Supplementary Figure 24).^33^ However, removal of the SUMO tag also did not significantly improve macrocyclization (Supplementary Figures 10 and 15).

**Figure 2:**
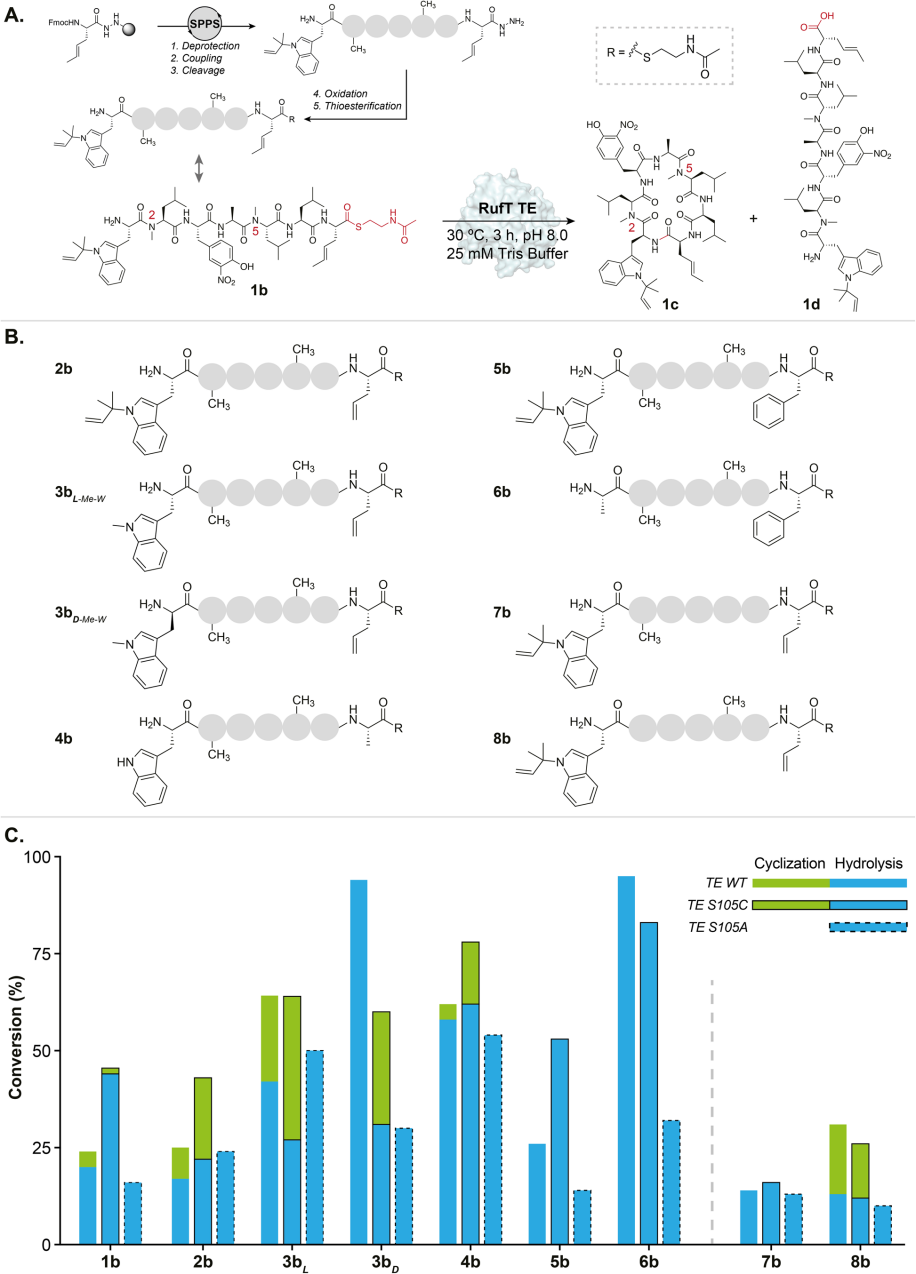
**Chemoenzymatic synthesis of rufomycin B and derivatives using RufT-TE.** (A) Solid-phase peptide synthesis (SPPS) of linear SNAC rufomycin peptides (see Supplementary Figure 1 for full details). SPPS (Chlorotrityl resin) results in C-terminal hydrazides that are oxidized to acyl azides using NaNO_2_ in pH 3 buffer (6 M GdmHCl, 50 mM NaH_2_PO_4_, 1.5 mM EDTA), −15 °C, 20 min. Thioesterification is achieved by adding *N*-acetylcysteamine (50 equiv), 15 min, 0 °C then neutralise with NaHCO_3_ to pH 7. RufT-TE cyclization of SNAC peptides: 12.5 μM enzyme, 50 μM SNAC peptide, 25 mM Tris buffer containing 300 mM NaCl, pH 8, 30 °C, 3 h, 800 rpm. (B) Library of SNAC peptides to study RufT-TE substrate tolerance. Peptides **1b**–**6b** contain sterically different C-terminal and N-terminal residues and the same peptide backbone. Peptides **2b**, **7b**, and **8b** contain different peptide backbones but the same C-terminal and N-terminal residues. All peptides were synthesized by SPPS. NAC derivatives were produced via acyl hydrazides and purified by preparative HPLC (Supplementary Figure S1). (C) Comparison of conversion of SNAC peptides **1b**–**8b** by RufT-TE and its variants. Conversion was calculated by HPLC monitored at 355 nm. Percent conversions in the RufT-TE assay were calculated by averaging the results of two repeats.

Type I thioesterases are part of the α/β-hydrolase superfamily. They contain a conserved catalytic triad of serine, histidine, and aspartate (Figure 1B). Like other serine hydrolases, TE domains act via a two-stage mechanism. The substrate is loaded to form an acyl enzyme intermediate which is subsequently activated via hydrogen bonding of the ester carbonyl to N–H in the protein backbone and subsequent stabilization of the tetrahedral intermediate in a so-called oxyanion hole (Figure 1B).^34^ The thioester peptide substrate could be hydrolyzed in the active site prior to loading, or water could compete with the amine nucleophile once the acyl enzyme intermediate has formed. To determine at which point in the mechanism hydrolysis occurs, catalytic serine 105 was mutated to alanine so that RufT-TE was incapable of loading the linear peptide (Supplementary Figure 11). Reaction of RufT-TE S105A with **1b** resulted in some hydrolysis of the SNAC peptide, although it does not account for all hydrolyzed product in the assay with wild-type enzyme. This indicates that water is also a competitive nucleophile in the reaction cyclization step (Figure 2C). We also created the serine to cysteine mutant RufT-TE S105C, which has been previously shown to improve cyclization activity.^35,36^ While the conversion of peptide **1b** to all products increased to 46%, surprisingly, cyclization was reduced compared to wild type (1.5%), indicating that loading of the SNAC peptide to form a thioester-based acyl enzyme was improved, but hydrolysis of this species still outcompeted cyclization.

We turned to exploring the substrate tolerance of RufT-TE. Previous reports indicate that changes at the N- and C-termini can dramatically affect enzymatic cyclization,^8,9,37^ and thus, peptides **2b**–**6b** allowed us to investigate this effect for RufT-TE. They were also assayed with the S105A mutant to gain insight into the origin of hydrolysis and the S105C mutant to determine the impact of a more reactive acyl enzyme intermediate on cyclization (Figure 2C). All of the substrate SNAC peptides **2b** to **6b** are accepted by RufT-TE, and in fact, two unnatural substrates, **2b** and **3b**_**L**_, give superior conversions to cyclized products compared to the native substrate **1b**. However, hydrolysis is a major competing reaction (Figure 2C and Supplementary Figures 16–23). We note that these data contrast with the corresponding chemical cyclization, where all peptides were efficiently cyclized with >65% conversion in 15 min.^12^ From peptides **1b**–**3b**_**L**_, where the steric bulk at the N- and C-termini is reduced, we see a significant increase in cyclization, with a significant portion of the hydrolysis in **2b** and **3b**_**L**_ occurring prior to loading. Monitoring the rate of cyclization versus wild type for **3b**_**L**_ confirms that hydrolysis is more rapid (Supplementary Figure 28). We note that while the conversion to hydrolyzed peptide is greater in S105A, hydrolysis occurs at a higher rate in the wild type. This indicates that hydrolysis occurs before peptide loading in RufT-TE but hydrolysis of the acyl enzyme intermediate is faster (Supplementary Figure 26). Peptide **4b** produces less cyclized product than **3b**_**L**_, indicating an interaction between the indole and alkene side chains and the protein. Peptides **5b** and **6b** intriguingly produced no cyclized product. The C-terminal phenylalanine appears to be too sterically bulky to allow cyclization in the active site. Indeed, our previous work on chemical cyclization of the corresponding C-terminal azide of peptide **5b** also gave the lowest conversion (65%, compared to ∼90% for other derivatives) due to a steric clash between the N- and C-termini.^12^ Intriguingly, however, unlike the wild type, the S105C mutant does produce a correlation in conversion to cyclic product based on sterics, with reduction in steric bulk at the N-terminus leading to greater conversion to cyclic product with the C-terminal alkene maintained.

To assess the impact of the configuration of the N-terminal peptide residue on cyclization, RufT-TE was assayed with **3b**_**D**_. While peptide loading appeared to be improved (∼60% for **3b**_**L**_ to over 90% for **3b**_**D**_), no enzymatically cyclized product was detected, although interestingly the peptide spontaneously cyclized (<1%) (Supplementary Figure 18). Other characterized thioesterases, including SurE,^9^ Skyxy-TE,^38^ PenA,^39^ Ulm16,^40^ and TryC-TE,^8^ are also stereoselective in this fashion. However, to our surprise, RufT-TE S105C did enable heterochiral lactamization to produce the cyclized product at significant levels (Figure 2C and Supplementary Figure 22). This could indicate that this mutation results in wider changes in the active site to allow accommodation of N-terminal 1-*N*-methyl-d-tryptophan rather than just a change in the reactivity of the acyl enzyme intermediate.

Finally, our chemical cyclization data indicate that backbone *N*-methylation is a crucial factor in facilitating cyclization, as removing a backbone methyl group from N5 or N2 was detrimental to successful cyclization (Figure 2).^12^ To test the tolerance of RufT-TE to changes in backbone methylation, the enzyme was assayed with peptides **7b** and **8b**. Only peptide **8b** produced cyclized product, with a similar ratio of hydrolyzed to cyclized product as the corresponding bismethylated peptide **2b**. However, peptide **7b** lacking the *N*-methyl near the C-terminus (N5) resulted in no cyclization (Figure 2A). This indicates that N5 methylation increases the backbone flexibility that is vital for enzymatic cyclization (Figure 2A). These results are aligned with our results for chemical cyclization indicating that the N5 methyl group is most important to enable the peptide to adopt a procyclization conformation.^12^ Pleasingly, many rufomycins have been isolated that lack the N2 methyl group, while the N5 methyl is usually maintained.^19^ Taken together, these data indicate that peptides “skipping” module 2 methylation can be processed by the whole NRPS, including the TE domain, while any skipping of module 5 methylation is more likely to result in hydrolysis by the TE domain (Figure 1).^19^

However, our data presented above do not entirely explain the high levels of hydrolysis observed for peptide **1b** containing the native rufomycin biosynthetic sequence. The use of standalone TE domains can contribute to low macrocyclization, as protein–protein interactions between the TE domain and adjacent cognate carrier protein (PCP or thiolation (T) domain) are important (Supplementary Figure 24). However, while the carrier protein may improve loading of the peptide and hydrophobicity of the active site, it would be expected that selectivity of cyclization be determined by the TE domain.^34^ Thus, a construct encoding *rufT-PCP-TE* was designed by aligning the RufT PCP-TE domain sequence with the structurally characterized EntF PCP-TE domain (PDB entry2ROQ)^41^ to identify the domain boundary. Recombinant His_6_-RufT-PCP-TE was thus successfully produced in *E. coli* Tuner DE3 in the apo form (Supplementary Figures 5, 9, 10D, and 24). Peptides **1b**–**8b** were assayed under the same conditions as for the monodomain (Figure 3). As anticipated, all peptides exhibited enhanced conversion to the cyclized product relative to the monodomain. This is attributable to the PCP domain impacting the conformation of the lid domain, reducing water in the active site. Unexpectedly, peptide **3b**_**D**_ is also cyclized by the didomain, acting similarly to the RufT-TE S105C mutant. Thus, surprisingly, the addition of PCP alters the stereoselectivity of the enzyme. The substantial conversion of this peptide to hydrolyzed product in the monodomain implies that the peptide is loaded but the acyl enzyme is hydrolyzed. However, in reducing water entry to the active site, the didomain perhaps allows sufficient time for peptide to cyclize **3b**_**D**_.

**Figure 3:**
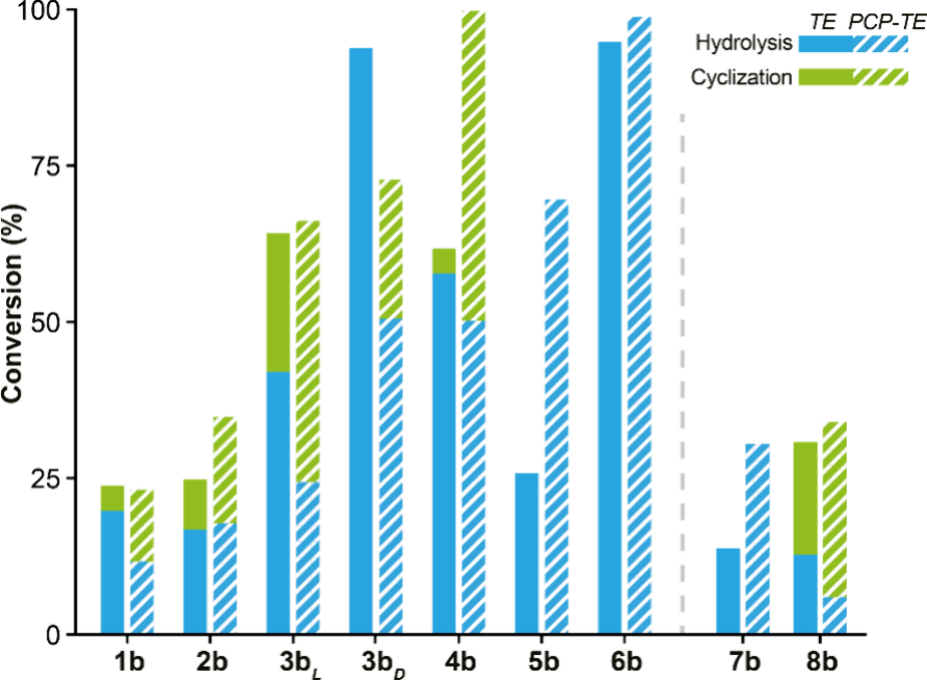
**Chemoenzymatic synthesis of rufomycin B and derivatives using didomain RufT-PCP-TE.** The chart shows conversion of SNAC peptides by RufT-PCP-TE to cyclic and hydrolysed peptides (striped) in comparison to RufT-TE (solid, as per Figure 2C). RufT-PCP-TE assay: 12.5 μM enzyme, 50 μM SNAC peptide, 25 mM Tris buffer containing 300 mM NaCl, pH 8, 30 °C, 3 h, 800 rpm. Conversion was calculated by HPLC monitored at 355 nm.

Even with improved macrocyclization using the didomain, hydrolysis is a significant side reaction. Reanalyzing our data, we noted that in several assays, a compound could be observed with the mass of the diketopiperazine natural product, rufomyazine **2e** (or corresponding derivative) (Figure 4A). This compound was previously isolated from *S. atratus* cultures.^13,42^ The identity of the peak in our assays was confirmed by comparison to synthesized rufomyazine **2e** (Figure 4C and Supplementary Figure 3). We previously proposed that rufomyazine could be the product of offloading from module 2 (Figure 1) but noted that there is no type II proofreading thioesterase in the rufomycin gene cluster.^13^ However, N-terminal degradation of linear peptides via diketopiperazine formation is a known process and in this case is likely to be facilitated by N2 methylation.^43^ To investigate further, we first interrogated whether peptides were impacted by sample extraction into ethyl acetate (EtOAc) prior to LCMS analysis. Thus, an assay of the model peptide **2b** was analyzed before and after EtOAc extraction. Indeed, rufomyazine **2e** is present in detectable amounts in the extracted sample (Figure 4B). Rufomyazine is extracted from *S. atratus* culture also using acidified EtOAc.^13^

**Figure 4:**
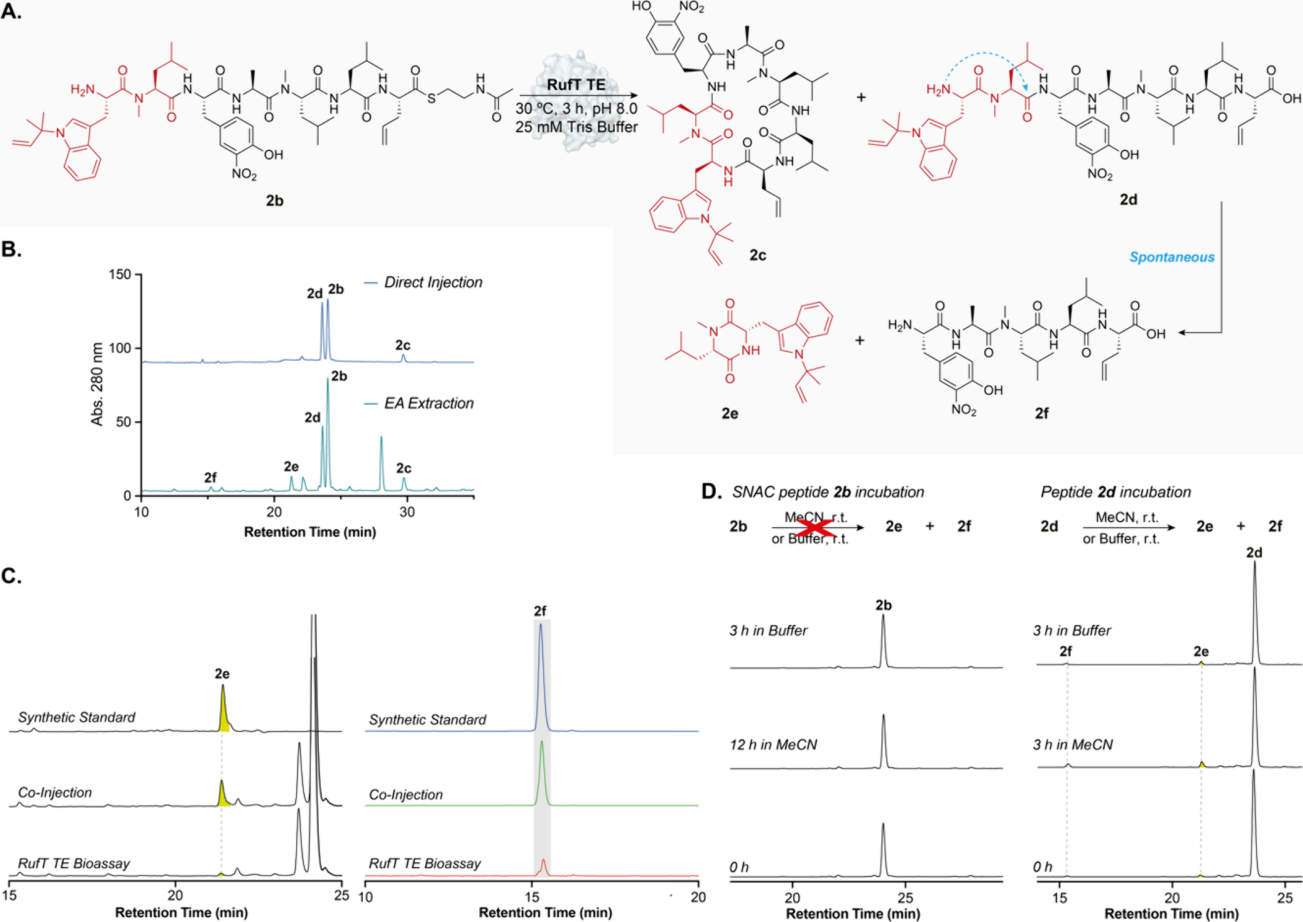
**Investigation of rufomyazine formation.** (A) RufT-TE reaction scheme using SNAC peptide **2b** illustrating formation of rufomyazine. The assay contained: 12.5 μM enzyme, 50 μM SNAC peptide, 25 mM Tris buffer containing 300 mM NaCl, pH 8, 30 °C, 3 h, 800 rpm. (B) HPLC analysis (280 nm) of the RufT-TE assay. Top chromatogram: direct injection of assay solution without ethyl acetate extraction. No rufomyazine product **2e** was detected. Bottom chromatogram: products were extracted from assay solution using EtOAc, and the residue was redissolved in MeCN. (C) Confirmation of peak identity by spiking assays with a synthetic standard of rufomyazine (Supplementary Figure 3). (D) HPLC analysis (280 nm) of linear rufomycin peptides in solution. Linear SNAC peptide **2b** was stable in RufT-TE assay buffer over 3 h or in MeCN over 12 h (left column). Linear peptide **2d** (200 μM) forms rufomyazine **2e** and linear pentapeptide **2f** in RufT-TE assay buffer or MeCN over 3 h (right column). Full timecourses are shown in Supplementary Figure S25. Note: MeCN was chosen because it is used to dilute samples for HPLC analysis.

To further analyze the stability, thioester **2b** was incubated in assay buffer (pH 8) or organic solvent (MeCN) over 3 h. The peptide remained intact, and no diketopiperazine was observed (note also that no spontaneous thioester hydrolysis was observed within 12 h). However, when hydrolyzed peptide **2d** was incubated under the same conditions, we observed the formation of rufomyazine **2e** and the corresponding pentapeptide **2f** in both (Figure 4D). Taken together, our data support the proposal that RufT naturally produces both cyclic and linear rufomycins. Thus TE-domain-catalyzed peptide hydrolysis is a feature of the enzyme. Our assays demonstrate that rufomyazine formation from linear rufomycin occurs in hours; however, like most streptomycetes, *S. atratus* is slow-growing, and extractions to isolate rufomycins are carried out after ∼6 days of growth.^44^ In that time, hydrolyzed linear rufomycin **1d** can degrade to rufomyazine **2e**, a process likely expedited by extraction of the culture medium into acidified EtOAc to isolate rufomycins. Therefore, rather than being a shunt metabolite resulting from module 2 offloading, rufomyazine is a product of slow degradation of linear rufomycin **1d** and thus an indicator of linear rufomycin formation by RufT-TE.

In conclusion, we report characterization of the recombinant macrocyclization enzyme RufT-TE both as a standalone domain and as a didomain with the adjacent PCP (RufT-PCP-TE). RufT-TE tolerates, and indeed prefers, substrates with changes in the N- and C-termini. However, its promiscuity is limited, and RufT-TE cannot cyclize all peptides known to be capable of cyclization based on our previous data.^12^ This provides further evidence for the role of TE domains as gatekeepers to peptide cyclization to ensure that incorrect sequences are not cyclized.^34^ We demonstrate that RufT-TE appears to be inefficient at cyclizing its native substrate, producing significant amounts of the hydrolyzed linear peptide. The PCP domain appears to be crucial for efficient cyclization by reducing peptide hydrolysis as well as enabling surprising flexibility in stereoselectivity. However, hydrolysis of the acyl enzyme intermediate remained a prominent reaction, and we propose that this is a feature of rufomycin biosynthesis leading to formation of the diketopiperazine rufomyazine **2e**. This allows us to propose a biosynthetic origin for this natural product as a degradation product of linear rufomycin **1d**. These data have significant implications for engineering RufT, as they imply that significant amounts of biosynthetic effort may be directed toward the linear peptide. We hope that this work will provide insight to enable both engineering of the rufomycin biosynthetic pathway^21^ and greater understanding of NRPS TE domains more generally.

## Methods

Full details of all methods, including small-molecule characterization and molecular biology, can be found in the Supporting Information.

## Supporting Information Available

All experimental details, including the synthesis and characterization of all small molecules and peptides and molecular biology and protein characterization, as well as supporting data (PDF)

## Abbreviations

EtOAc: ethyl acetate
NRcP: nonribosomal cyclic peptide
DCM: dichloromethane
TE: thioesterase
NRPS: nonribosomal peptide synthetase
DMF: dimethylformamide
MeCN: acetonitrile
SPPS: solid-phase peptide synthesis
