# Supplementary material for: Characterization of RufT Thioesterase Domain Reveals Insights into Rufomycin Cyclization and the Biosynthetic Origin of Rufomyazine
Source: ACS Chem Biol. 2025 Mar 6;20(3):573–80. doi: 10.1021/acschembio.4c00802 (PMC11934086; doi:10.1021/acschembio.4c00802)
Supplement: Supplementary file 1 — cb4c00802_si_001.pdf [file cb4c00802_si_001.pdf]

Supplementary information for:

Characterisation of RufT thioesterase domain reveals insights into rufomycin cyclization and the biosynthetic origin of rufomyazine.

Yaoyu Ding, Gustavo Perez Ortiz, Alexandra-Georgiana Butulan‡, Hamzah Sharif‡ and Sarah M. Barry*

Department of Chemistry, Faculty of Natural, Mathematical and Engineering Sciences, King's College London, Britannia House, 7 Trinity Street, London, SE1 1DB, UK

* To whom correspondence should be addressed
Email: sarah.barry@kcl.ac.uk
‡ denotes equal contribution

Table of Contents

1. General Materials **3**

2. Supplementary Methods **4**

2.1 General procedure for SNAC-peptide synthesis *4*

2.2 General Procedure for bioinspired peptide cyclization (semi-prep scale) *5*

2.3 Reverse-Phase Chromatography Methods *6*

2.3.1 Analytical HPLC 6

2.3.2 Semi-preparative HPLC 6

2.3.3 Preparative HPLC 7

2.3.4 UPLC-HRMS 7

2.4 General synthesis of Rufomyazine 2e *8*

2.4.1 Synthesis of Fmoc-*N*Me-*L*-Leu-*N*-(1,1-dimethyl-1-allyl)-*L*-Trp-OMe (**10**). 8

2.4.2 Synthesis of (3S,6S)-6-isobutyl-1-methyl-3-((1-(2-methylbut-3-en-2-yl)-1H-indol-3-yl)methyl)piperazine-2,5-dione (**2e**). 9

2.5 Cloning of RufT-TE and Protein Overproduction *11*

2.5.1 RufT-TE and RufT-PCP-TE construct design 11

2.5.2 Cloning the thioesterase domain of RufT to create overexpression plasmid RufT-TE-His_6 13

2.5.3 Subcloning *rufT-TE* to create overexpression His_6-SUMO-RufT-TE plasmid 14

2.5.4 Overexpression and purification of His_6-SUMO-RufT-TE and His_6-RufT PCP-TE 17

2.5.5 Protein quantification and size estimation of recombinant protein 18

2.5.6 Cleavage of the His_6-SUMO tag from His_6-SUMO-RufT TE with ULP-1 19

2.5.7 General procedure for site-directed mutagenesis of RufT-TE 20

2.6 Biochemical Characterisation of His_6-SUMO-RufT-TE *21*

2.6.1 General procedure for RufT-TE catalyzed peptide cyclization 21

3. Supplementary Figures **22**

4. Supplementary Characterization Data **43**

4.1 ^1H and ^{13}C NMR spectra *43*

4.2 High Resolution Mass Spectra and Fragmentation of Linear Peptides *45*

4.3 High Resolution UPLC-HRMS analysis of His_6-SUMO-RufT TE *84*

5. Supplementary References **87**

1. General Materials

All the reagents and solvents were obtained from commercial suppliers and were used without further purification. Dry reactions were conducted in oven-dried glassware under a nitrogen atmosphere. Anhydrous THF, MeOH and DCM were obtained from the Inert Solvent Purification System, all other solvents were supplied as Sureseal® bottles by Sigma Aldrich, DMF (sequencing grade) was purchased from Cambridge Reagent Ltd used for Solid Phase Peptide Synthesis (SPPS). Nuclear Magnetic Resonance (NMR) spectra were recorded using a Bruker UltraShieldTM 400 MHz or 700 MHz and at default temperature (18 ºC). The chemical shifts (δ) are reported in parts per million (ppm) using the abbreviations: s, singlet; d, doublet; dd, double of doublet; t, triplet; q, quartet. Resonances that could not be easily interpreted were designated multiplets (m). Chemical shifts (δ) are referenced to the residual solvent signal. Spin-spin coupling constants *J* are quoted in Hz. Flash column chromatography was performed using 60 Å (40-64 micron) silica and solvent mixtures of hexane and ethyl acetate or DCM and MeOH. Analytical thin layer chromatography was performed on TLC Silica gel 60 F_{254} (Aluminium sheets). Visualization was assisted with 254 nm UV-lamp, potassium permanganate ($KMnO_4$) stain and ninhydrin stain. High-resolution mass spectra were recorded on a Waters Acquity UPLC-Class I equipped with an ACQUITY UPLC column. The detector was a Waters Xevo-G2-XS QTof with electrospray ionization source. The instrument was operated in positive mode full-scan with detection window set from 50 to 2000 Da. For peptide fragmentation, a collision energy ramp from 15 V to 40 V was employed. For protein production a New Brunswick Scientific Inova 44 was used for incubation and a Beckmann Coulter Avanti J-26 XP-1 for centrifugation. Protein purification was carried out using ÄKTA Pure Chromatography System (GE).

2. Supplementary Methods

2.1 General procedure for SNAC-peptide synthesis

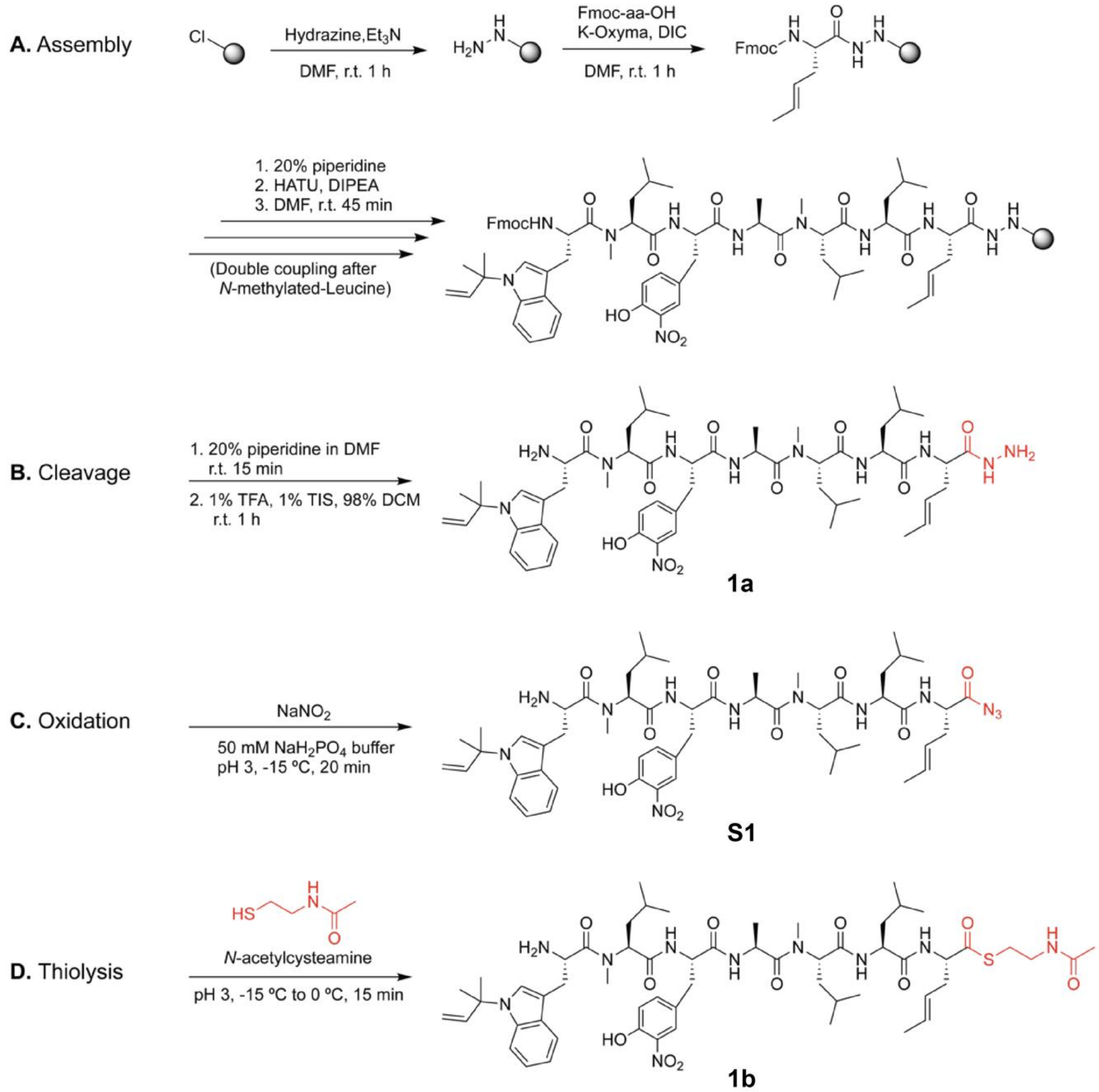

Supplementary Figure 1. General synthesis of peptide hydrazide through Fmoc-strategy SPPS and one-pot synthesis of SNAC-peptide. Exemplified by the synthesis of the native linear rufomycin peptide **1**. All SNAC rufomycin derivatives were synthesised in the same fashion.

The hydrazide linear peptide was prepared using solid phase peptide synthesis as previously described.[1] The linear hydrazide peptide was used directly after cleavage without further purification. All the buffer solutions were freshly prepared and degassed before using. It should be noted 5 M sodium nitrite stock was prepared as using degassed water to dissolve sodium nitrite. Typically, to a suspension of crude hydrazide linear peptide (1 equiv., 1.5 ~ 4 mM) in phosphate buffer pH 3 (6 M GdmHCl, 50 mM NaH_2PO_4, 1.5 mM EDTA) was added sodium nitrite stock (20 equiv.) at -15 ºC (Supplementary Fig. 1C). Then the solution was left to stir for 20 min before *N*-

acetylcysteamine (50 equiv.) was added (Supplementary Fig. 1D). The resulting mixture was stirred for 15 min at 0 ºC, then neutralized with saturated sodium bicarbonate to pH 7 as measured with pH paper and the resulting solution was extracted with ethyl acetate (3 x 10 mL). Additonal water (2 mL) was added to facilitate better separation. Combined organic layers were concentrated *in vacuo* at room temperature and redissolved in 80% acetonitrile in H_2O, then purified through HPLC using different gradients (70% ~ 85% isolated yields).

Gradient 2 for SNAC peptide **1b**

Gradient 3 for SNAC peptide **3b** (Both *D*-1Me-Trp and *L*-1Me-Trp)

Gradient 4 for SNAC peptide **5b**

Gradient 5 for SNAC peptide **2b**, **7b** and **8b**

Gradient 6 for SNAC peptide **4b**, **6b** and penta linear peptide **2f**

2.2 General Procedure for bioinspired peptide cyclization (semi-prep scale)

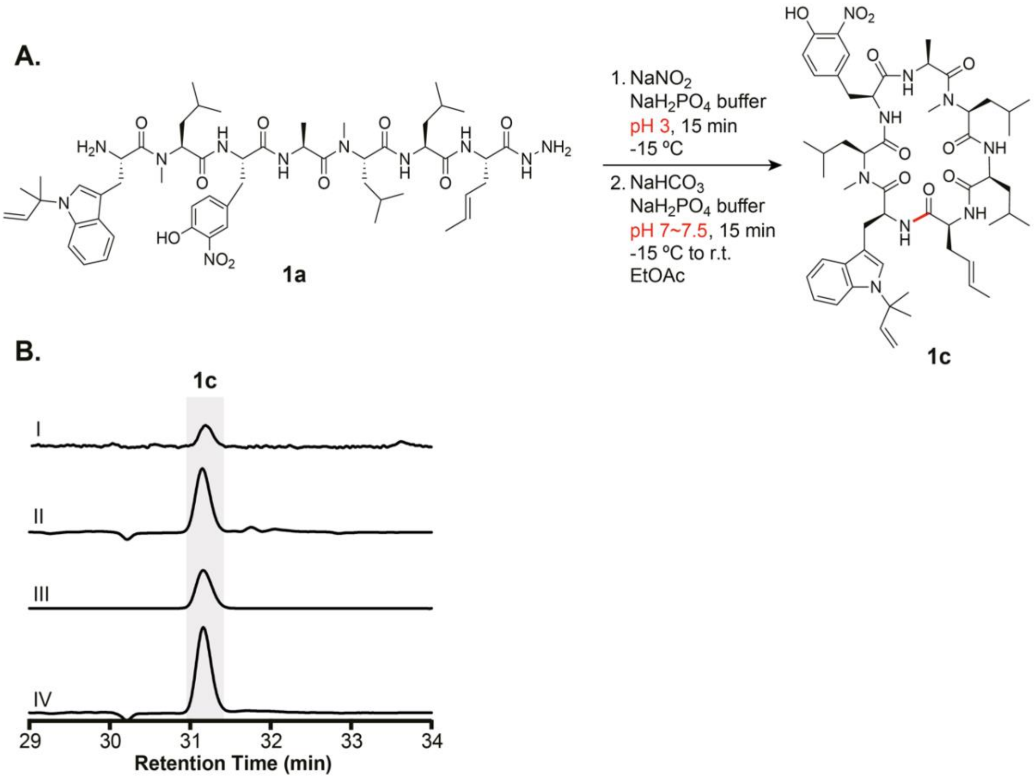

Supplementary Figure 2. Chemical cyclization of natural product rufomycin B 1c. A. One-pot style fast macrocyclization to produce rufomycin B including oxidation of C-terminal acyl hydrazide peptide to acyl azide followed by in-situ head to tail cyclization. **B.** HPLC comparison of cyclic product of RufT-TE domain assay (I), rufomycin B bacteria extract (II) and chemical cyclization (III). (IV) co-injection of I, II and III. All UV-vis traces were recorded at 355 nm.

The synthesis of rufomycin B was carried out as previously reported.[1] Brieflly, to a suspension of crude hydrazide linear peptide **1a** (0.03 mmol, 1 equiv., 3 mM) in 10 mL of phosphate buffer (6 M GdmCl, 50 mM NaH_2PO_4, 1.5 mM EDTA, pH 3) added

sodium nitrite (0.60 mmol, 20 equiv.) at -15 °C under nitrogen. The resulting solution was stirred for 20 min at -15 °C before gently raising up the pH to 7~7.5 with saturated sodium bicarbonate. 15 mL (1.5 volume) of ethyl acetate was added and the mixture was stirred vigorously for 15 min. Then the solution was further extracted with ethyl acetate (3 x 15 mL). The combined organic layers were concentrated *in vacuo* at room temperature and redissolved in 80% acetonitrile in water, then purified through preparative HPLC using Gradient: 30% to 70%B over 40 min using a reversed phase Agilent Zorbax 300SB-C18 PrepHT 21.2 x 150 mm 7 μm column was used at a constant flow rate of 20 mL/min at room temperature. After lyophilization, 11 mg of rufomycin B **1c** was harvested as a yellowish powder. (HPLC yield: 84%, isolated yield: 39%).

2.3 Reverse-Phase Chromatography Methods

Analytical high-performance liquid chromatography (HPLC) was performed on an Agilent 1260 Infinity II instrument equipped with a DAD detector with an Agilent Eclipse XDB-C18 5 μm 4.6 x 150 mm column at a constant flow rate of 1 mL/min at 40 ºC. The UV-vis absorbance of the eluent was monitored at 220 nm, 280 nm and 355 nm. Typical gradients using mixture of two solvents are described below.

Solvent A: H_2O containing 0.1% TFA

Solvent B: MeCN containing 0.1% TFA

Solvent C: H_2O containing 0.1% FA

Solvent D: MeCN containg 0.1% FA

2.3.1 Analytical HPLC

Gradient 1: linear gradient from 5-100% B over 40 min, wash 5 min with 100% B, then back to 5% B over 3 min.

2.3.2 Semi-preparative HPLC

In semi-preparative mode, an Agilent ZORBAX 300SB-C18 5 μm 9.4 x 250 mm column was used at a constant flow rate of 3.5 mL/min at 60 ºC.

Gradient 2: 2 min with 20% B, linear gradient from 20%~70% B over 30 min, then 70%~95% B over 1 min, wash 5 min with 95% B, then back to 20% B over 2 min.

2.3.3 Preparative HPLC

HPLC purification of the peptides was performed on an Agilent 1260 Preparative HPLC system using a reversed phase Agilent Zorbax 300SB-C18 PrepHT 21.2 x 150 mm 7 µm column was used at a constant flow rate of 20 mL/min at room temperature.

Gradient 3: linear gradient from 30%~45% B over 30 min, then 45%~95% B over 1 min, wash 2 min with 95% B, then back to 30% B over 2 min.

Gradient 4: linear gradient from 40%~60% B over 30 min, then 60%~95% B over 1 min, wash 2 min with 95% B, then back to 40% B over 2 min.

Gradient 5: linear gradient from 30%~70% B over 40 min, then 70%~95% B over 1 min, wash 2 min with 95% B, then back to 30% B over 2 min.

Gradient 6: 2 min with 5% B, linear gradient 5%~60% B over 40 min, then 60%~95% B over 1 min, wash 2 min with 95%B, then back to 5 % B over 2 min.

2.3.4 UPLC-HRMS

All hydrazide peptides were analyzed on a Waters ACQUITY UPLC BEH C8, 1.7 µm, 2.1 x 50 mm column, the rest of peptides on a Waters ACQUITY UPLC BEH C18, 1.7 µm, 2.1 x 50 mm column, the proteins were analyzed on a Waters ACQUITY UPLC BEH C4, 300 A, 1.7 µm, 2.1 x 50 mm column with constant flow of 0.4 mL/min 40 ºC. High-resolution mass spectra were recorded on a Waters Acquity UPLC-Class I equipped with an ACQUITY UPLC column. The detector was a Waters Xevo-G2-XS QTof with electrospray ionization source. The instrument was operated in positive mode full-scan with detection window set from 50 to 2000 Da. For peptide fragmentation, a collision energy ramp from 15 V to 40 V was employed. Typical gradients are described below.

Gradient 7: 0.5 min with 5% solvent D in solvent C, linear gradient from 5~95% solvent D in C over 4 min, wash 1 min with 95% solvent D in C, then back to 5% D in C over 0.5 min and then 0.5 min with 5% solvent D in C.

2.4 General synthesis of Rufomyazine 2e

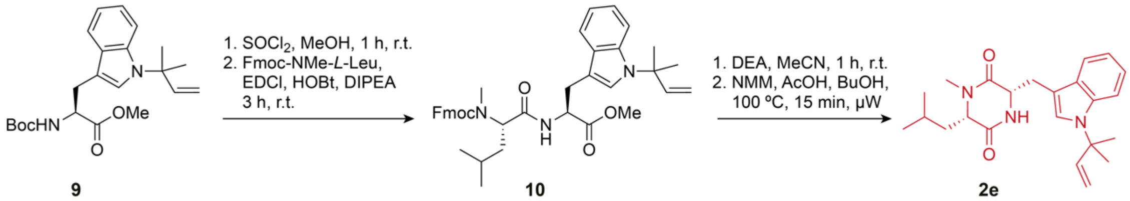

9 | **10** 55% | **2e** 90%

Supplementary Figure 3. Synthesis of rufomyazine 2e.[2]

2.4.1 Synthesis of Fmoc-*N*Me-*L*-Leu-*N*-(1,1-dimethyl-1-allyl)-*L*-Trp-OMe (10).

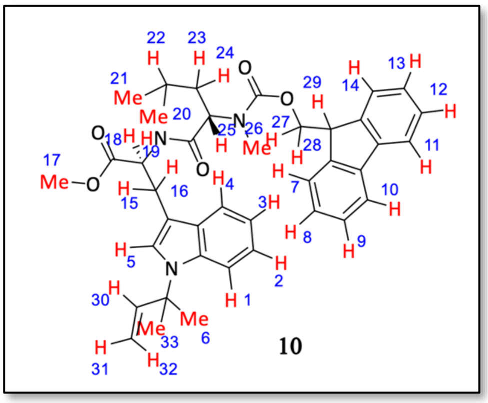

10

Compound **9** was prepared as previously reported by us.[1] A solution of **9** (121 mg, 0.31 mmol) in MeOH (4 mL) was added to an ice cooled solution of thionyl chloride (33.8 µL, 0.47 mmol, 1.5 equiv) in methanol (4 mL) while stirring at 50 ºC for 2h, to give *N*-(1,1 dimethyl-1-ally)-*L*-Trp-OMe hydrochloride, which was taken directly over and used. To a 0 ºC solution of Fmoc-NMe-*L*-Leu (110 mg, 0.31 mmol) in DCM (13 mL), HOBt (46 mg, 0.34 mmol, 1.1 equiv.), EDCI (65 mg, 0.34 mmol, 1.1 equiv.), and DIPEA (0.6 mL, 0.34 mmol, 1.1 equiv.) were successively added and stirred under nitrogen for 20 min. A solution of DIPEA (0.6 mL, 0.34 mmol, 1.1 equiv.) and *N*-(1,1 dimethyl-1-ally)-*L*-Trp-OMe hydrochloride (0.31 mmol) in DCM (13 mL) was then added to the reaction and allowed to stir (~ 3 h) at r.t. until completion (TLC analysis, 40% ethyl acetate : Hexane). The reaction was then quenched with NH_4Cl solution at 0 ºC and extracted with DCM (3 × 10 mL), with the organic layer being dried and concentrated to produce a crude foam, which was purified using silica gel chromatography (20% ethyl acetate : Hexane) to give 108.50 mg (yield 55% over 2 steps) of the product **10** as a white solid.

^{1}H NMR ($CDCl_3$, 400 MHz) δ 7.69-6.97 (13H, m, H1-5,7-14), 6.63 (1H, s, H19) 5.98 (1H, m, H30), 5.04 (2H, m, H31,32), 4.75 (1H, m, H29), 4.62 (1H, m, H25), 4.36 (1H, m, H18), 4.28 (1H, m, H15), 4.14 (1H, m, H16), 3.61 (3H, m, H26), 3.18 (2H, m, H27,28), 2.54 (1.7H, s, H17), 2.41 (1.3H, s, H17) 1.60 (6H, s, H6,33), 1.55 (1H, m, H23), 1.26 (2H, m, H22,25), 0.84-0.63 (6H, m, H21,22).

^{13}C NMR ($CDCl_3$, 100 MHz) δ 171.20 (C23), 170.48 (C25), 157.02 (C32), 144.04 (C38), 143.84-107.34 (20C, C1-20), 135.51 (C39), 67.60 (C21), 59.00 (C26), 57.00 (C33), 52.96 (C31), 52.36 (C34), 47.25 (C22), 36.65 (C27), 29.60 (C36), 27.84 (C37), 25.78 (C28), 23.24 (C29), 21.82 (C30).

2.4.2 Synthesis of (3S,6S)-6-isobutyl-1-methyl-3-((1-(2-methylbut-3-en-2-yl)-1H-indol-3-yl)methyl)piperazine-2,5-dione (2e).

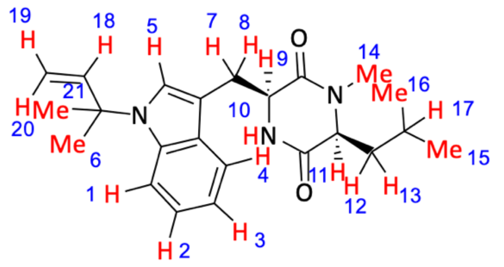

2e

Compound **10** (30 mg, 0.047 mmol) was dissolved in acetonitrile (1 mL) and treated with diethylamine (1 mL) at r.t. and stirred (~ 1 hr) until completion (TLC analysis, 20% Ethyl acetate : Hexane). The solution was concentrated and the crude residue *N*-Me-*L*-Leu-*N*-(1,1-dimethyl-1-allyl)-*L*-Trp-OMe (0.047 mmol) was placed directly in a 10 mL microwave vessel along with acetic acid (0.24 mL), *N*-methylmorpholine (0.16 mL) and butanol (1.6 mL). The mixture was heated to 100 °C in microwave for 15 min, at which time TLC analysis (3% MeOH/DCM) was performed. The solvents were evaporated, and crude residue was purified by prep HPLC to give 16.34 mg (yield 91%) of the product **2e** as a white fluffy solid.

$[\alpha]^{25}$D = + 10.26 (c = 0.05, MeOH).

^{1}H NMR ($CDCl_3$, 400 MHz) δ 7.50 (1H, d, *J* = 8 Hz, H1), 7.41 (1H, d, *J* = 8 Hz, H4), 7.07 (1H, s, H5), 7.04 (2H, m, H2,3), 6.04 (1H, dd, *J* = 8, 16 Hz, H10,18), 5.10 (2H, m, H19,20), 4.21 (1H, m, H9), 3.70 (1H, q, *J* = 4 Hz, H11), 3.41 (1H, dd, *J* = 4, 8 Hz, H7), 3.08 (1H, dd, *J* = 8,12 Hz, H8), 2.88 (3H, s, H14), 1.70 (1H, m, H17), 1.67 (3H, s, H6),

1.66 (3H, s, H21), 1.23 (2H, m, H12,13), 0.84 (3H, d, *J* = 4 Hz, H15), 0.77 (3H, d, *J* = 4 Hz, H16).

^{13}C NMR ($CDCl_3$, 100 MHz) δ 168.04 (C16), 165.91 (C19), 143.95 (C12), 135.78 (C6), 128.93 (C8), 124.45 (C7), 121.30 (C1), 119.44 (C2), 118.81 (C3), 114.02 (C4), 113.67 (C13), 107.66 (C5), 60.30 (C18), 59.11 (C9), 56.77 (C15), 42.60 (C20), 33.07 (C17), 31.81 (C14), 27.91 (C10), 27.86 (C11), 25.02 (C21), 23.11 (C22), 22.11 (C23).

HRMS $[M + H]^+$ (ESI) m/z 382.2490 (calc. for $C_{23}H_{32}N_3O_2$, 382.2489, *Δ* = 0.26 ppm).

2.5 Cloning of RufT-TE and Protein Overproduction

2.5.1 RufT-TE and RufT-PCP-TE construct design

The Ruf-TE and PCP domain sequences were identified using the PKS/NRPS Analysis http://nrps.igs.umaryland.edu/.[3] The sequence was aligned, using Clustal Omega, with other TE domains including SrfC_A, a structurally characterized termination module from surfactin biosynthesis, to determine conserved regions. Analysis of the SrfA-C crystal structure allowed us to identify the end of the first *N*-terminal beta sheet of the TE domain (Supplementary Fig. 4). RufT-PCP-TE domain boundary was identified by alignment with structurally characterised EntF-PCP-TE (PDB 2ROQ) (Supplementary Figure 5)

```
RufT_TE         --HD-----------VDEGAGR---SASSRGPSSAFEVLLPLRTKGDRAPLFCLHSGGGM 44
SrfA_C_TE       ------------------------------GGSDGLQDVTIM-NQDQEQIIFAFPPVLGY 29
FenB_TE         ---------------------SNQLS------AAGEQHVIQL-NQQGGKNLFCFPPISGF 32
GrsB_TE         ITHQESENNVHQPILVNVEADREALSLNGEKQRKNIELPILL-NEETDRNVFLFAPIGAQ 59
TycC_TE         -----------------------------KNFEGNSGSAILL-NEEMARKVFCFTPIGAQ 30
                                                         : .:     :* :    .

RufT_TE         SWNYASLLPHIGADIPVYGLQARGLSDPDDLPGSVEEVADDCIEAMVRVQPEGPYRLMGH 104
SrfA_C_TE       GLMYQNLSSRLP-SYKLCAFDFIE----------EEDRLDRYADLIQKLQPEGPLTLFGY 78
FENB_TE         GIYFKDLALQLNHKAAVYGFHFIE----------EDSRIEQYVSRITEIQPEGPYVLLGY 82
GrsB_TE         GVFYKKLAEQIP-TASLYGFDFIE----------DDDRIQQYIESMIQTQSDGQYVLIGY 108
TycC_TE         SVYYQKLADEIK-GVSLYSFDFIQ----------EDNRLEQYIEAIVAIDPQGPYTLMGY 79
                .  : .*  .:     : .:.              :.  :   . :   : :*   *:*:

RufT_TE         SFGGIVAHAVAARLAERGQQVELIVCLDAKPAEDEEDIPEHGHEEYYRGILELLGVSTAE 164
SrfA_C_TE       SAGCSLAFEAAKKLEEQGRIVQRIIMVDSYKKQGVSDLDGRTVESDVEALMNVNRDN--- 135
FenB_TE         SAGGNLAFEVVQAMEQKGLEVSDFIIVDAYKKDQSITADTENDDS--------------- 127
GrsB_TE         SSGGNLAFEVAKEMERQGYSVSDLVLFDVYWKGKVFEQTKEEEEENIKIIMEELREN--- 165
TycC_TE         SSGGNLAFEVAKELENQGYVVTDLILFDSYWKDKVMERTLAETESDITQLFAEIGEN--- 136
                * *  :*. ..  : .:*  *  :: .*               :.

RufT_TE         LPVEDLTFEDFAAVARTTNTVLGSIEE-SEFLTVMRVMENNIEITKGYRHRQVATEMMLF 223
SrfA_C_TE       ------------EALNSEAVKHGLKQKTHAFYSYYVNL-----IS----TGQVKADIDLL 174
FenB_TE         ------------AAYLPEAVRETVMQKKRCYQEYWAQL-----IN----EGRIKSNIHFI 166
GrsB_TE         PGMFNMTREDFELYFANEFVKQSFTRKMRKYMSFYTQL-----VN----YGEVEATIHLI 216
TycC_TE         IEMFNMTQEDFNLYAANEFVKQSFIRKTVSYVMYHNQL-----IN----TGATSAAIHLI 187
                                   .     .:   :      :     :.         : : ::

RufT_TE         AATQETD-------------TVLE----PDVWHDYLAGPLEYRRMDCSHAGMLKPEVLSQ 266
SrfA_C_TE       TSGADFD----------------MPEWLA-SWEEATTGVYRVKRGFGTHAEMLQGETLDR 217
FenB_TE         EAGIQTET----------SGAMVLQKWQDA----A-EEGYAEYTGYGAHKDMLEGEFAEK 211
GrsB_TE         QAEFEEEKIDENEKADEEEKTYLEEKWNEKAWNKA-AKRFVKYNGYGAHSNMLGGDGLER 275
TycC_TE         QSELESG-----------EDDLVAVKWNETAWAQA-TKRLMTYEGYGIHSRMLGGNYVSM 235
                 :  :                                           *  **  :  .

RufT_TE         IGTLIQDRLRRGPAGSR------     283
SrfA_C_TE       NAEILLEFLNTQTVTVS------     234
FenB_TE         NANIILNILDKINSDQKVLPNKH     234
GrsB_TE         NSSILKQILQGTFVVK-------     291
TycC_TE         NASILREILQELFILK-------     251
                 . :: : *
```

Supplementary Figure 4. Alignment of RufT-TE with TE domains of SrfA_C (PDB: 2VSQ), FenB (NCBI: WP_127695096), GrsB (NCBI: CAA43838.1), TycC_TE (NCBI: WP_106655228). Sequence in red indicates disordered loop between two beta sheets. Orange line indicates start of first structural feature based on crystal structure. Red line indicates RufT-TE domain start based on analysis using

PKS/NRPS Analysis. Solid blue line indicates start of RufT-TE used for construct design. Conserved, catalytic triad, residues are highlighted in yellow.

```
RufT        DRLPGYMVPAAVTALPELPLSPNGKVDRKALPAPDYGGRAGRPPRTPQEEVLCTLFAEVL
EntF        -------------MLPELKAQAPG-----------------RAPKAGSETIIAAAFSSLL
                         ****  .. *                 *.*:: .* ::.: *:.:*

RufT        GLPGVGVDDGFFDLGGHSLLATGLISRIQTVLGVDLPLRILFEASTVAELAQRLDHDVDE
EntF        GCDVQDADADFFALGGHALLAMKLAAQLSRQVARQVTPGQVMVASTVAKLATIIDAEEDS
            *    ..* .** ****:***  * :::.  :. ::.   :: *****:**  :* : *.

RufT        GAGRSASSRGPSSAFEVLLPLRTKGDRAPLFCLHSGGGMSWNYASLLPHIGADIPVYGLQ
EntF        ---------TRRMGFETILPLRE-GNGPTLFCFHPASGFAWQFSVLSRYLDPQWSIIGIQ
                         .**.:****  *: ..***:*...*::*::: *  ::..: .: *:*

RufT        ARGLSDPDDLPGSVEEVADDCIEAMVRVQPEGPYRLMGHSFGGIVAHAVAARLAERGQQV
EntF        SPRPNGPMQTAANLDEVCEAHLATLLEQQPHGPYYLLGYSLGGTLAQGIAARLRARGEQV
            :   ..* : ...::**.:  : :::. **.*** *:*:*:** :*:.:****  **:**

RufT        ELIVCLDAKPAEDEEDIPEHGHEEYYRGILELLGVSTAELPVEDLTFEDFAAVARTTNTV
EntF        AFLGLLDTWPPETQNWQEKEANGLDPEVLAEINREREAFLAAQ-----------------
             ::  **: *.* ::   :..:    . : *:     * *..:

RufT        LGSIEESEFLTVMRVMENNIEITKGYRHRQVATEMMLFAATQETDTVLEPDVWHDYLAGP
EntF        QGSTSTELFTTIEGNYADAVRLLTTAHSVPFDGKATLFVAERTLQEGMSPERAWSPWIAE
             ** . . * *:     : :.: .  :   .  :  **.* :  :  :.*:   .   .

RufT        LEYRRMDCSHAGMLKPEVLSQIGTLIQDRLRRGPAGSR--
EntF        LDIYRQDCAHVDIISPGTFEKIGPIIRATLNRLEHHHHHH
            *:  * **:*..::.* .:.:**.:*:  *.*     :
```

Supplementary Figure 5. Alignment of RufT PCP-TE domain with EntF PCP-TE domains (PDB: 2ROQ).[4] Conserved, catalytic triad, residues are indicated under red cycles. Blue arrow indicates RufT PCP-TE domain start based on analysis using PKS/NRPS Analysis. Red arrow indicates start of RufT PCP-TE used for construct design.

2.5.2 Cloning the thioesterase domain of RufT to create overexpression plasmid RufT-TE-His_6

The DNA sequence encoding the *C*-terminal thioesterase domain of the NRPS RufT (NCBI: WP_114243093.1 from NZ_CP027306.1) represents the last 273 amino acid residues (819 bp) of the multimodular enzyme (7991 amino acid residues), identified from the published BGC.[5] The sequence was partially codon optimized for its expression in *E. coli* and was purchased from Integrated DNA Technologies (IDT) (Supplementary Fig. 6).

```
RufT_WT_sequence          GCGAGCTCCAGGGGACCGTCCAGTGCCTTCGAGGTGCTGCTGCCGCTGCGCACCAAGGGC  60
RufT_codon_optimized      GCGAGCTCCAGGGGACCGTCCAGTGCCTTCGAGGTGCTGCTGCCGCTGCGCACCAAGGGC  60
                          ************************************************************

RufT_WT_sequence          GACCGGGCGCCCCTGTTCTGTCTGCACTCGGGCGGCGGGATGAGCTGGAATTACGCGAGC  120
RufT_codon_optimized      GACCGGGCGCCCCTGTTCTGTCTGCACTCGGGCGGCGGGATGAGCTGGAATTACGCGAGC  120
                          ************************************************************

RufT_WT_sequence          CTGCTGCCGCACATCGGCGCCGACATCCCTGTATACGGCCTCCAGGCGCGTGGCCTGTCG  180
RufT_codon_optimized      CTGCTGCCGCACATCGGCGCCGACATCCCTGTATACGGCCTCCAGGCGCGTGGCCTGTCG  180
                          ************************************************************

RufT_WT_sequence          GATCCGGACGACCTGCCGGGGTCGGTCGAGGAGGTGGCCGACGACTGCATCGAGGCGATG  240
RufT_codon_optimized      GATCCGGACGACCTGCCGGGGTCGGTCGAGGAGGTGGCCGACGACTGCATCGAGGCGATG  240
                          ************************************************************

RufT_WT_sequence          GTCCGGGTGCAGCCCGAAGGGCCGTACCGGCTGATGGGGCACTCCTTCGGGGGCATCGTC  300
RufT_codon_optimized      GTCCGGGTGCAGCCCGAAGGGCCGTACCGGCTGATGGGGCACTCCTTCGGGGGCATCGTC  300
                          ************************************************************

RufT_WT_sequence          GCCCACGCGGTGGCGGCCCGGCTGGCCGAGCGCGGTCAGCAGGTCGAACTGATCGTCTGC  360
RufT_codon_optimized      GCCCACGCGGTGGCGGCCCGGCTGGCCGAGCGCGGTCAGCAGGTCGAACTGATCGTCTGC  360
                          ************************************************************

RufT_WT_sequence          CTCGACGCGAAGCCCGCCGAGGACGAGGAGGACATCCCCGAGCACGGGCACGAGGAGTAC  420
RufT_codon_optimized      CTCGACGCGAAGCCCGCCGAGGACGAGGAGGACATCCCCGAGCACGGGCACGAGGAGTAC  420
                          ************************************************************

RufT_WT_sequence          TACCGGGGGATCCTCGAACTGCTGGGCGTGAGCACCGCCGAACTACCGGTGGAGGACCTG  480
RufT_codon_optimized      TACCGGGGGATCCTCGAACTGCTGGGCGTGAGCACCGCCGAACTACCGGTGGAGGACCTG  480
                          ************************************************************

RufT_WT_sequence          ACGTTCGAGGATTTCGCAGCGGTGGCCCGGACGACCAACACCGTCCTCGGCAGCATCGAG  540
RufT_codon_optimized      ACGTTCGAGGATTTCGCAGCGGTGGCCCGGACGACCAACACCGTCCTCGGCAGCATCGAG  540
                          ************************************************************

RufT_WT_sequence          GAGAGCGAGTTCCTCACCGTCATGCGGGTGATGGAGAACAACATCGAGATCACCAAGGGC  600
RufT_codon_optimized      GAGAGCGAGTTCCTCACCGTCATGCGGGTGATGGAGAACAACATCGAGATCACCAAGGGC  600
                          ************************************************************

RufT_WT_sequence          TATCGGCACCGACAGGTCGCAACCGAGATGATGCTGTTCGCGGCCACGCAGGAGACCGAC  660
RufT_codon_optimized      TATCGGCACCGACAGGTCGCAACCGAGATGATGCTGTTCGCGGCCACGCAGGAGACCGAC  660
                          ************************************************************

RufT_WT_sequence          ACCGTCCTGGAGCCGGATGTGTGGCACGATTACCTTGCCGGGCCACTGGAGTACCGGCGC  720
RufT_codon_optimized      ACCGTCCTGGAGCCGGATGTGTGGCACGATTACCTTGCCGGGCCACTGGAGTACCGGCGC  720
                          ************************************************************

RufT_WT_sequence          ATGGACTGCTCCCACGCCGGCATGCTCAAGCCGGAGGTGCTGAGCCAGATCGGCACGTTG  780
RufT_codon_optimized      ATGGACTGCTCCCACGCCGGCATGCTCAAGCCGGAGGTGCTGAGCCAGATCGGCACGTTG  780
                          ************************************************************

RufT_WT_sequence          ATCCAGGACCGCCTGCGGCGCGGACCCGCCGGTTCCCGGTAG    822
RufT_codon_optimized      ATCCAGGACCGCCTTAGACGGGGACCAGCGGGTTCTCGGTAA    822
                          **************  * ** ***** ** ***** *****
```

Supplementary Figure 6. Sequence alignment of the last 822 bp of NRPS RufT (NCBI: LC257593.1) of *S. atratus* and the codon optimized sequence for heterologous expression in *E. coli*.

The sequence included 4 base pairs (CACC) at the 5' end necessary for directional cloning in a TOPO cloning reaction (Thermofisher). The stop codon was omitted as the recombinant protein was designed as a *C*-terminal His-tagged protein. The DNA

sample was diluted to 2 ng µL^{-1} for cloning into pET101/D-TOPO plasmid as directed by the Invitrogen TOPO cloning manual.

2-3 µL of the cloning mixture was added to TOP10 cells (25 µL), stored on ice for 30 min before being incubated (42 ºC, 42 s) followed by addition of 250 µL ice-cold LB. The cells were incubated (37 ºC, 180 rpm, 60 min) and plated out onto LB agar (100 µg mL^{-1} carbenicillin). Colonies picked from these plates were used to inoculate 10 mL LB (100 µg mL^{-1} of carbenicillin) and incubated at 37 ºC for 16 h. Plasmid DNA was purified from these overnight cultures using Qiagen kit according to manufacturers instructions. Clones were confirmed as containing the correct gene inserts by Sanger sequencing by Genewiz. The resulting plasmid was named GPO101-TE01. (Supplementary Fig. 7)

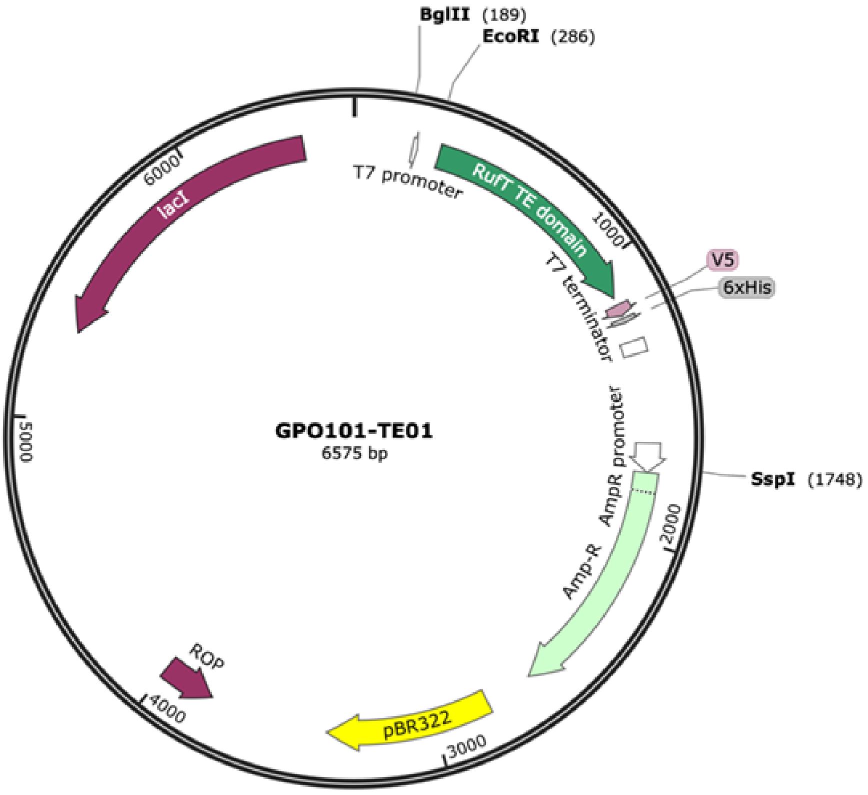

Supplementary Figure 7. Plasmid map of GPO101-TE01. The map shows the relative location of the TE domain of gene *rufT* and structural features, for example, an ampicillin resistance gene (AmpR), used for selection. The vector contains incorporates a 6x polyhistidine tag for protein purification by IMAC, a TEV cleavage site and T7 promoter and lac repressor protein (lacI) for IPTG-induced gene expression.

2.5.3 Subcloning *rufT-TE* to create overexpression His_6-SUMO-RufT-TE plasmid

GPO101-TE01 encoding His_6-RufT-TE resulted in poor protein solubility. To improve RufT-TE overproduction, the *rufT-TE insert* was subcloned by PCR from the GPO101-

TE01 plasmid and assembled into a pET15b plasmid (SUMOpET15b, kind donation from Manuel Mueller, KCL) using the NEBuilder High-Fidelity DNA Assembly Cloning Kit from New England Biolabs®.

Supplementary Table 1 shows the sequence of the oligos for each plasmid and the annealing temperatures. The primers were designed using the NEBuilder Assembly Tool (http://nebuilder.neb.com).

Supplementary Table 1. Oligos used for each PCR reaction designed with the NEBuilder Assembly Tool. The oligos forward and reverse use to amplify *rufT-TE* contain 21 nucleotides extra, that overlap the SUMOpET15b sequences on both ends. This allows the assembly of both DNA fragments. Plasmid specific sequences are underlined.

Primer sequences 5' – 3'	Anneals	Overlaps	F/R	Annealing Temp (°C)
ACCACCAATCTGTTCTCTGTGAG	SUMOpET15b	GPO101-TE01	R	65.0
ACAGAGAACAGATTGGTGGTGCGAGCTCCAGGGGACCG	GPO101-TE01	SUMOpET15b	F	72.0
TGTTAGCAGCCGGATCC**TTA**CCGAGAACCCGCTGGTCC	GPO101-TE01	SUMOpET15b	R	72.0
**TAA**GGATCCGGCTGCTAAC	SUMOpET15b	GPO101-TE01	F	65.0

Each PCR reaction was prepared in triplicate with the following reagents: 5 x Q5 reaction buffer (10 μL), dNTP mix (10 mM each, 1 μL), primers (2.5 μL each of 10 μM stocks), 5x Q5 GC enhancer (10 μL) Q5 High-Fidelity DNA polymerase (New England Biolabs) (0.5 μL) and DNA template (1 μL, 3 ng) and water up to 50 μL, respectively. The PCR cycle parameters are shown in Supplementary Table 2.

Supplementary Table 2. PCR cycle parameters for the amplification of the PCR fragments. X= annealing temperature according to **Supplemantary Table 1**.

Cycles	Temperature (°C)	Time
**1**	68.5	1 min
**30**	98<br>**X**<br>72	10 sec<br>20 sec<br>3 min
**1**	72	7 min

PCR reactions were analysed by agarose gel electrophoresis (90 V for 1 hr). The bands of the correct size were cut, and the DNA fragment was extracted using the

QIAquick Gel Extraction Kit (Qiagen). DNA concentration was determined using a Labtech Nanodrop ND-8000 spectro-photometer. The assembly reaction was carried out in duplicate and was prepared as follows: 1 µL plasmid (35 ng), 1 µL gene insert (70 ng), 5 µL HiFi DNA Assembly Master Mix (New England Biolabs) and 2.5 µL water. The reaction was incubated 45 min at 50 ºC. 2 µL of the assembly reaction was used to transform NEB 5-alpha competent *E. coli* cells. Colonies were isolated on LB plates (50 µg mL^{-1} carbenicillin). Positive clones were confirmed by restriction digest (Supplementary Fig. 5A), using 500 ng of DNA, 5 µL (1X) of CutSmart buffer 10X (NEB), 1 µL EcoRI (NEB), water up to 50 µL and incubated 1 hr at 37 ºC and by sequencing (Genewiz). The resulting plasmid was named GPO15b-TE02 and encodes the fusion protein His_6-SUMO-RufT-TE (Supplementary Fig. 8B).

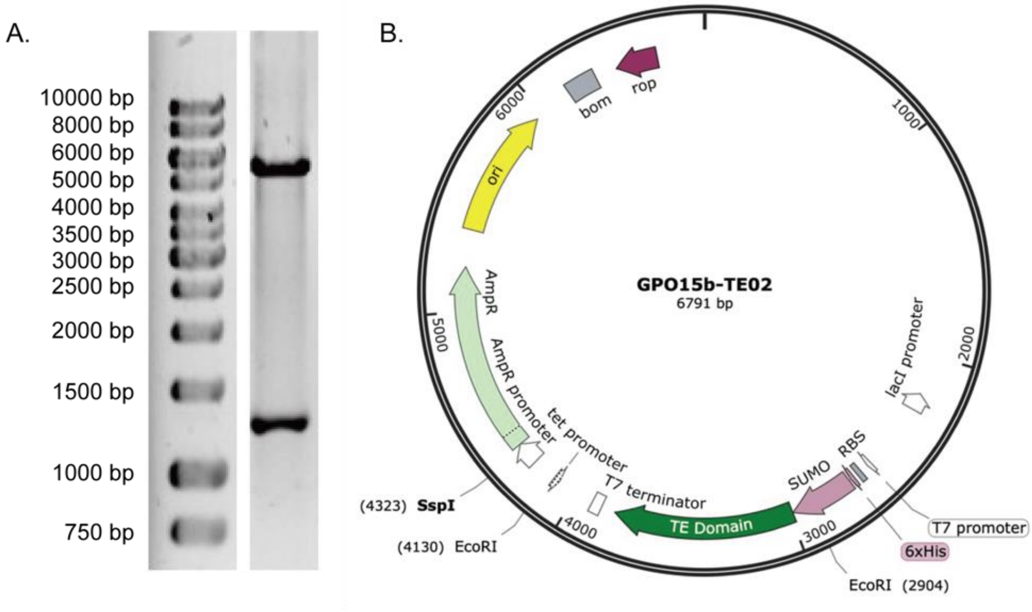

Supplementary Figure 8. Plasmid map of GPO15b-TE02 and restriction digestion of the plasmid. A. Restriction digest of GPO15b-TE02 showing the two bands expected of 5565 and 1226 bp, respectively in the presence of restriction enzyme EcoRI. **B**. Plasmid map of GPO15b-TE02. The map shows the relative location of the TE domain of RufT and structural features, for example, Small Ubiquitin-like Modifier (SUMO) solubility partner tag, an ampicillin resistance gene (AmpR), used for selection, a 6x polyhistidine tag for protein purification with IMAC, a TEV cleavage site and T7 promoter and lac repressor protein (lacI) for IPTG-induced gene expression.

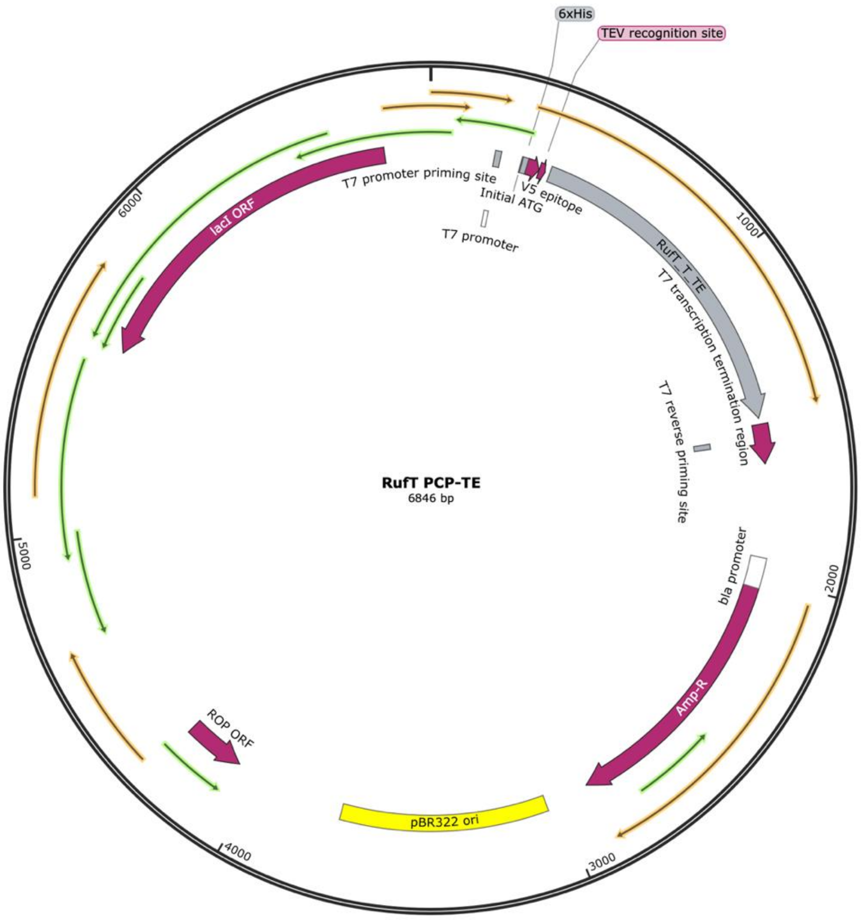

Supplementary Figure 9: Plasmid map of pET151-*rufT*- PCP-TE construct. The map shows the relative location of the insert encoding PCP-TE domain of RufT and structural features, for example, an ampicillin resistance gene (Amp-R), used for selection, a 6x polyhistidine tag for protein purification with IMAC, a TEV cleavage site and T7 promoter and lac repressor protein (lacI) for IPTG-induced gene expression.

2.5.4 Overexpression and purification of His_6-SUMO-RufT-TE and His_6-RufT PCP-TE

pET151-*rufT-P*CP-TE construct was purchased from Thermo Fisher Scientific, GPO15b-TE02 and *rufT*-PCP-TE construct were used to transform competent *E. coli* Tuner-DE3 (Sigma-Aldrich) cells according to manufacturer's instructions. Transformation mixtures were plated on LB agar (50 µg mL^{-1} carbenicillin) and incubated overnight at 37 ºC. Single colonies of transformed cells were grown in 10 mL of LB liquid media (50 µg mL^{-1} of carbenicillin) and incubated overnight at 37 ºC with shaking (220 rpm). 500 mL of pre-warmed LB media (50 µg mL^{-1} carbenicillin) was inoculated with 3 mL of the overnight culture and incubated further at 37 ºC with shaking (230 rpm). Once the OD_{600} reached 0.6, the cultures were cooled to 15 ºC and induced with isopropyl-β-D-thiogalactopyranoside (IPTG) to a final concentration of 0.5 mM and induced cultures were incubated overnight at 15 ºC with shaking (230 rpm). Cells were harvested by centrifugation (4200 rpm, 20 min, 4 ºC), the supernatant

was discarded. The pellet was resuspended in the minimum volume of Buffer A (100 mM NaCl, 20 mM Tris Base, pH 8, 20 mM imidazole, 10% v/v glycerol), adding dithiothreitol (DTT) (final conc. 0.5 mM), Pepstatin A (final conc. 1 mg mL^{-1}), DNAse A (final conc. 0.2 mg mL^{-1}) and Complete™ protease inhibitor cocktail (1 tablet per 2 L of culture) (Sigma-Aldrich). Cell lysis was carried out on a cell disruptor IXT4A (Constant System LTD) with a pressure of 25 kPa at 4 ºC. The lysate was centrifuged (18,000 rpm) for 42 min at 4 ºC. Protein purification was carried out using ÄKTA Pure Chromatography System (GE). Chromatograms at 280 nm was monitored during the purification. The AKTA system was washed with degassed water and Buffer A. The 5 mL His-Trap™ Fast Flow nickel affinity column (GE) was equilibrated with 35 mL (7 column volume) of Buffer A. The supernatant was then loaded on to the column and washed with Buffer A, until there is no decrease in the 280 nm absorbance. The mobile phase was then changed to Buffer B (100 mM NaCl, 20 mM Tris Base, pH 8, 200 mM imidazole, 10% v/v glycerol) from 0% to 100% Buffer B over 30 mL then 100% Buffer B with 20 mL, the eluent was collected in fractions. The fractions were analyzed by SDS-PAGE gel and fractions containing the protein of interest (Supplementary Fig. 9A) were combined, exchange Buffer B to Buffer C (100 mM NaCl, 20 mM Tris Base, pH 8, 10% v/v glycerol) through vivaspin concentrator (30 kDa) and concentrated to a volume of 2.5 mL. The resulting solution was direcly used for enzymatic macrocyclization without further purification.

2.5.5 Protein quantification and size estimation of recombinant protein

The Bradford assay was used to estimate protein concentration. A stock solution of 1 mg mL^{-1} of bovine serum albumin (BSA) was diluted in 5 different concentrations in triplicates (ranging from 0 to 250 µg mL^{-1}). The dilutions were prepared in Buffer C. 1 mL of Bradford reagent was added to 50 µL of each dilution. After vortexing it for a few seconds and waiting for 5 min, the absorbance at 595 nm was recorded using a Biochrom Libra S22 UV/Vis Spectrophotometer. With the triplicates a calibration curve was plotted, with a minimum R2 value of 0.99, then was used for protein quantification.

A portion of concentrated protein solution (freshly prepared) was injected onto a Superdex 200 10/300 GL Size Exclusion Chromatography column (GE) equilibrated with 1.2 column volume of Buffer C (100 mM NaCl, 20 mM Tris Base, pH 8, 10% v/v glycerol) which also acted as the mobile phase. The protein eluent with another 1.2

column volume of Buffer C. The eluent volume of His6-SUMO-RufT-TE (42.5 kDa) observed was 14 mL (Supplementary Fig. 10B), the elution volume indicates that the protein is dimeric.

2.5.6 Cleavage of the His6-SUMO tag from His6-SUMO-RufT TE with ULP-1

After the purification of His6-SUMO-RufT TE through the 5 mL His-Trap™ Fast Flow nickel affinity column (GE), imidazole was removed from the protein through buffer exchange in a viva-spin concentrator (30 kDa). ULP1 protease was added into the cleavage buffer (25 mM Tris, 150 mM NaCl, 1 mM DTT, pH 8) at a of ratio 1:3, ULP1 protease (5 μM final conc.) to His6-SUMO-RufT TE (15 μM final conc.). After a gentle manual mixing, the cleavage solution was left at 4 ºC for 2 h. Next, the cleavage solution was concentrated down to 1 mL then loaded on a pre-equilibrated (Buffer A) 5 mL His-Trap™ Fast Flow nickel affinity column (GE) and washed with 5 column volume of Buffer A. The fractions correspond to RufT TE were combined and concentrated using a vivaspin concentrator (10 kDa) to 0.5 mL for further *in vitro* bioactivity test (Supplementary Fig. 10C).

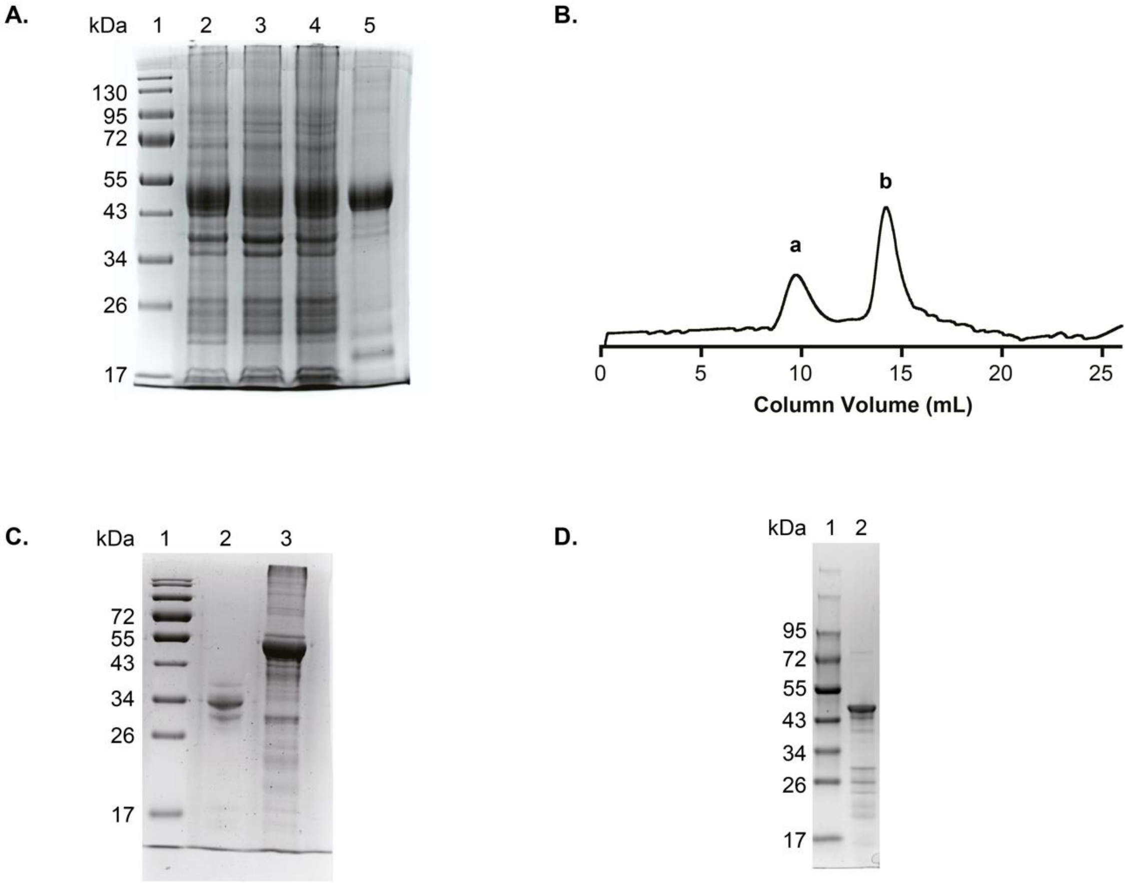

Supplementary Figure 10. Biochemical Characterization of His6-SUMO-RufT-TE and cleaved RufT-TE. A. SDS-PAGE gel of recombinant His6-SUMO-RufT-TE. Lane 1 is protein ladder, lane 2 is

cell pellet, lane 3 is cell lysate, lane 4 soluble crude protein and lane 5 is purified His_6-SUMO-RufT-TE (42.5 kDa). **B.** Size-exclusion chromatography of recombinant His_6-SUMO-RufT-TE. A small portion of purified His_6-SUMO-RufT TE into the Superdex 200 10/300 GL SEC column and eluent with Buffer C. Peak **a** eluted around 10 mL is soluable aggregates and peak **b** eluted around 14 mL is His_6-SUMO-RufT-TE as a dimer. **C.** SDS-PAGE gel of SUMO-tag cleavage RufT-TE. Lane 1 is protein ladder, lane 2 is RufT-TE (30 kDa) and lane 3 is starting His_6-SUMO-RufT-TE (42.5 kDa). **D.** SDS-PAGE gel of RufT PCP-TE. Lane 1 is protein ladder, lane 2 is RufT-PCP-TE (43.09 kDa).

2.5.7 General procedure for site-directed mutagenesis of RufT-TE

Primers were designed using NEBaseChanger. The PCR reaction mixture included: 1 µL of GPO15b-TE02 plasmid (diluted to 10 ng/µL), 5 µL of Q5 reaction buffer, 10 µM of both forward and reverse primers (listed below), 10 mM dNTP, 0.5 µL of Q5 DNA polymerase, and 15.75 µL of dH_2O to a final volume of 25 µL. The mixture was placed in a thermocycler and subjected to the following program: initial denaturation at 98 ºC for 30s, followed by 35 cycles of 98 ºC for 10s, annealing at the recommended temperature (Ta) for 20s, and extension at 72 ºC for 3 min. After the cycles, a final extension was performed at 72 ºC for 5 min. The PCR product was then incubated overnight at 37 °C with Dpn1 and purified using a PCR clean-up kit.

For ligation, the following mixture was incubated at room temperature for 4 h: 10 µL of PCR product, 2 µL of 10x kinase buffer, 2 µL of 10x ligase buffer, 2 µL of 100 mM ATP, 1 µL of 200 mM DTT, 1 µL of PNK, and 1 µL of DNA ligase. The reaction mixture (5 µL) was used to transform *E. coli* TOP10 cells, which were then streaked on an 'agar plate containing 100 µg/mL ampicillin and incubated overnight at 37 ºC. Single colonies were selected and grown overnight at 37 ºC in 5 mL of LB medium with 100 µg/mL ampicillin, shaking at 220 rpm. The cells were lysed, and the plasmid was purified using a plasmid miniprep kit.

**Point mutation**	**Codon change**	**Forward Primer (5'-3')**	**Reverse Primer (5'-3')**
**S105C**	TCC-T**G**C	TACCGGCTGATGGGGCA C*T**G**C*TTCGGGGGCATC GTCGCC	GGCGACGATGCCCC CGAA*G**C**A*GTGCCCC ATCAGCCGGTA
**S105A**	TCC-**G**CC	TACCGGCTGATGGGGC AC***G**CC*TTCGGGGGCAT CGTCGCC	GGCGACGATGCCCC CGAA*GG**C***GTGCCCC ATCAGCCGGTA

Supplementary Table 3. Primers used in this study for RufT-TE site-directed mutagenesis.

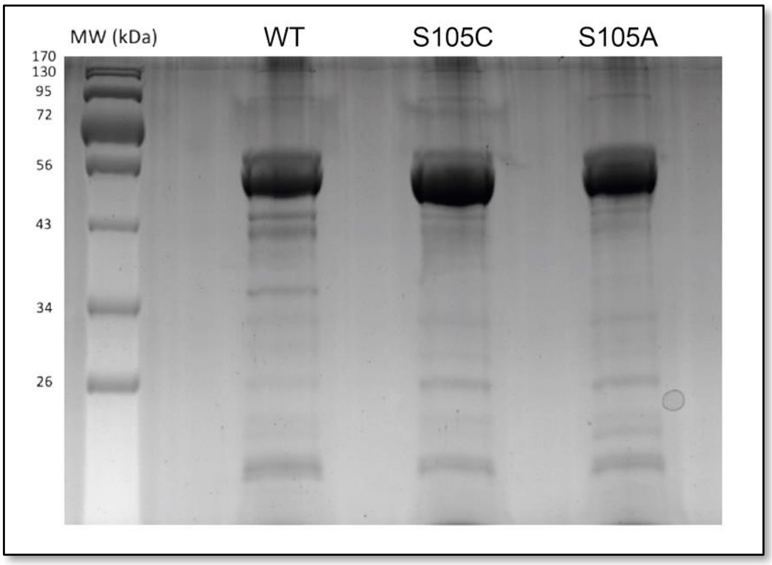

Supplementary Figure 11. SDS PAGE analysis of His$_6$-SUMO-RufT-TE WT in comparison with mutants S105C & S105A following NTA purification. Protein MW= 42.5 kDa

2.6 Biochemical Characterisation of His$_6$-SUMO-RufT-TE

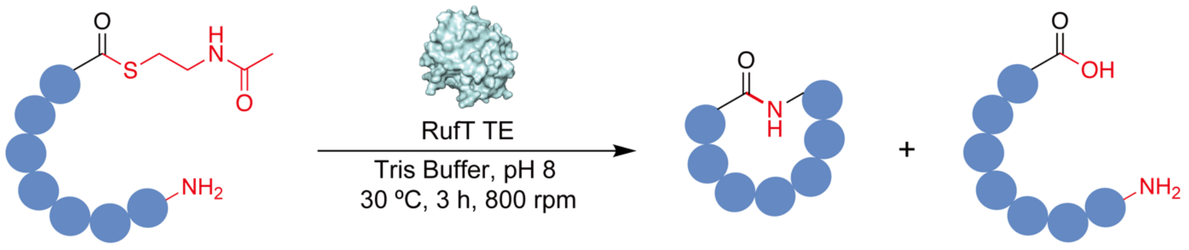

Supplementary Figure 12. RufT-TE *in vitro* assay. Incubation of linear SNAC-peptide **b** with RufT-TE to afford cyclic peptide **c** and hydrolysed linear peptide **d**.

2.6.1 General procedure for RufT-TE or RufT-PCP-TE catalyzed peptide cyclization

Analytical enzymatic cyclization was performed at a total volume of 200 µL. Typically, to SNAC peptide **b** (50 µM final conc.) in Tris Buffer (25 mM, 300 mM NaCl, pH = 8) containing 5 % DMSO, RufT-TE or RufT-PCP-TE (12.5 µM final conc.) was added to initialize the reaction. The resulting mixture was incubated for 3 h at 30 ºC with shaking 800 rpm. The reaction was extracted with ethyl acetate (3 x 400 µL). The combined organic layers were dried and redissolved in 40 µL 80% acetonitrile in H_2O. The sample was analyzed via HPLC using Gradient 1. All bioassays were carried out using the same batch of the purified enzymes. The negative control used the same

conditions as above but RufT-TE was heat inactivated by heating the protein at 100 ºC for 15 min.

3. Supplementary Figures

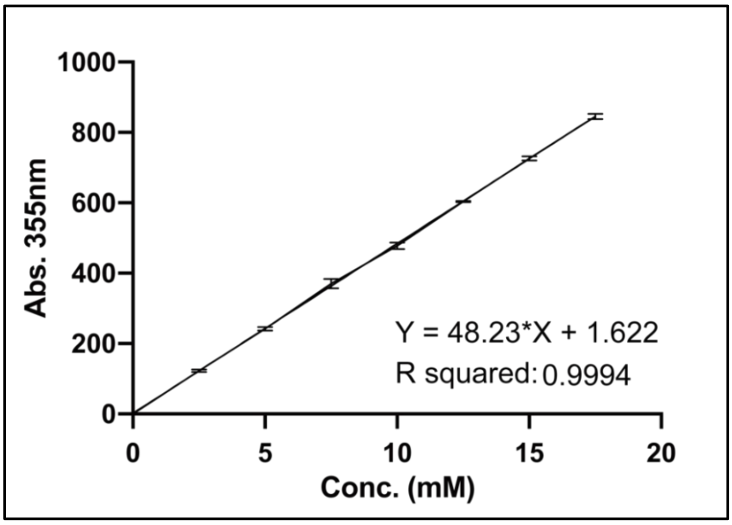

Supplementary Figure 13. Fmoc-3-nitro-L-tyrosine calibration curve. Fmoc-3-nitro-L-tyrosine stock solution (20 mM) was prepared in triplicate in 3 mL volumetric flask. 26.9 mg of Fmoc-3-nitro-L-tyrosine were dissolved in acetonitrile, few DMF was required to solublise compound. HPLC samples were diluted to six different concentrations, then each sample was injected 1 µL into HPLC. Absorbance under specific 355 nm was recorded and a calibration curve was plotted, the R squared value is 0.9994.

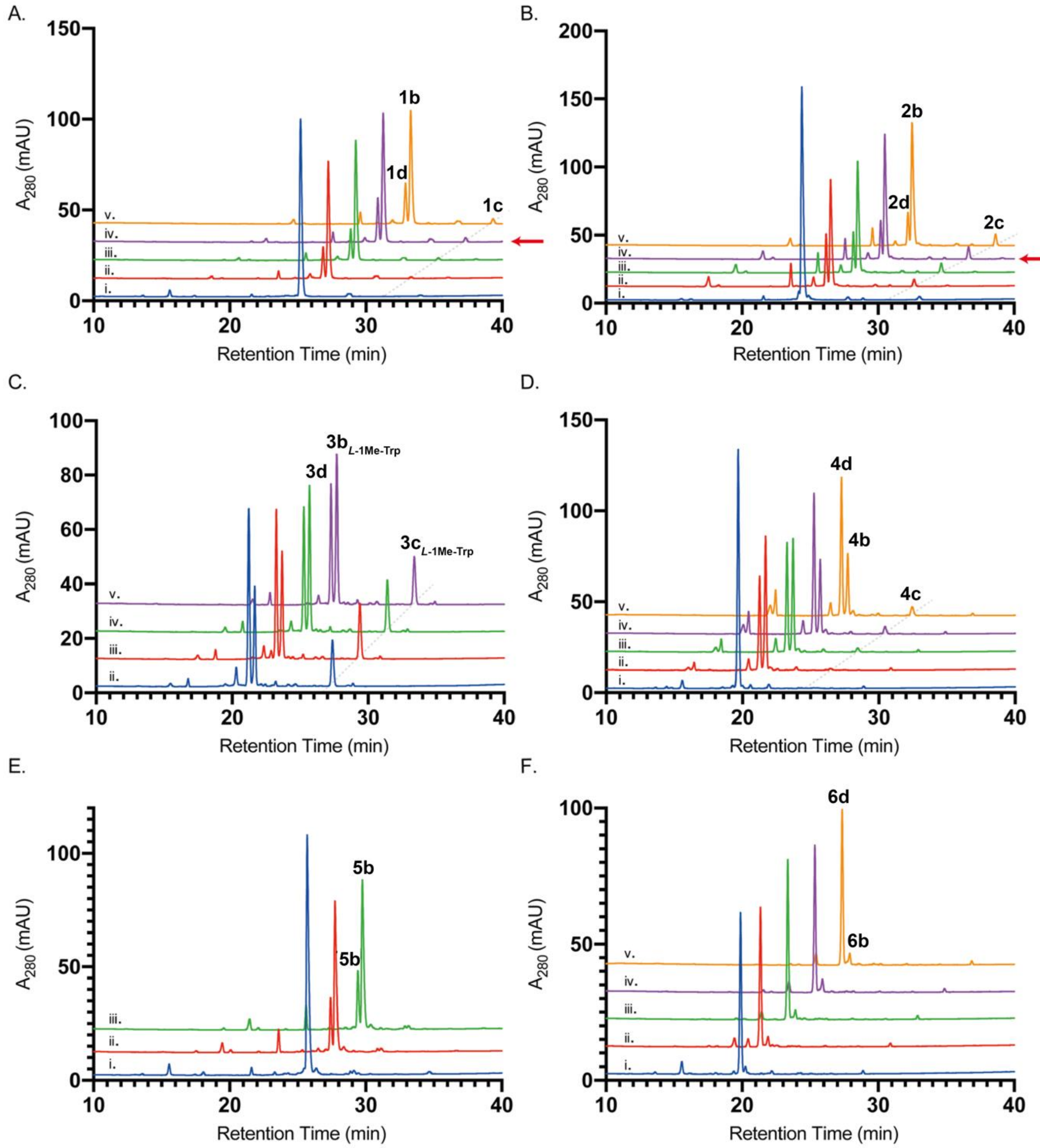

Supplementary Figure 14. HPLC analysis of His$_6$-SUMO-RufT-TE bioassay optimization of substrate 1b to 6b (ionic strength screening). Chromatograms i is the negative control with boiled enzyme. Chromatogram ii bioassay performed in Tris buffer no NaCl added in. Chromatogram iii 100 mM NaCl added. Chromatogram iv 300 mM NaCl added and Chromatogram v adding 500 mM NaCl added. Only miner difference was observed when 300 mM NaCl added.

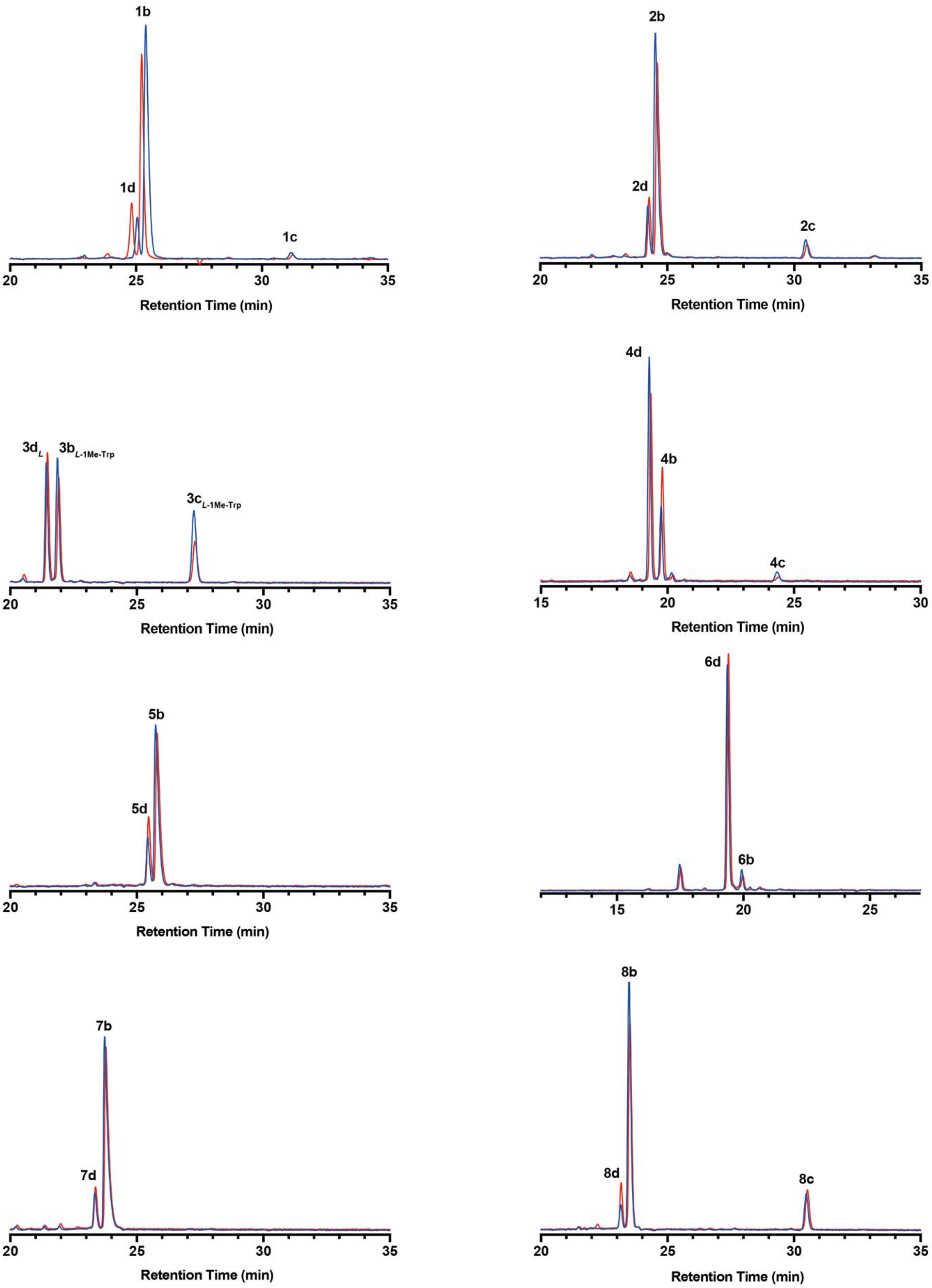

Supplementary Figure 15. HPLC analysis of bioassays catalyzed by RufT-TE vs. His$_6$-SUMO RufT-TE with different substrates. For comparison, chromatograms were overlayed. RufT-TE (dark blue) vs. His$_6$-SUMO-RufT-TE (red).

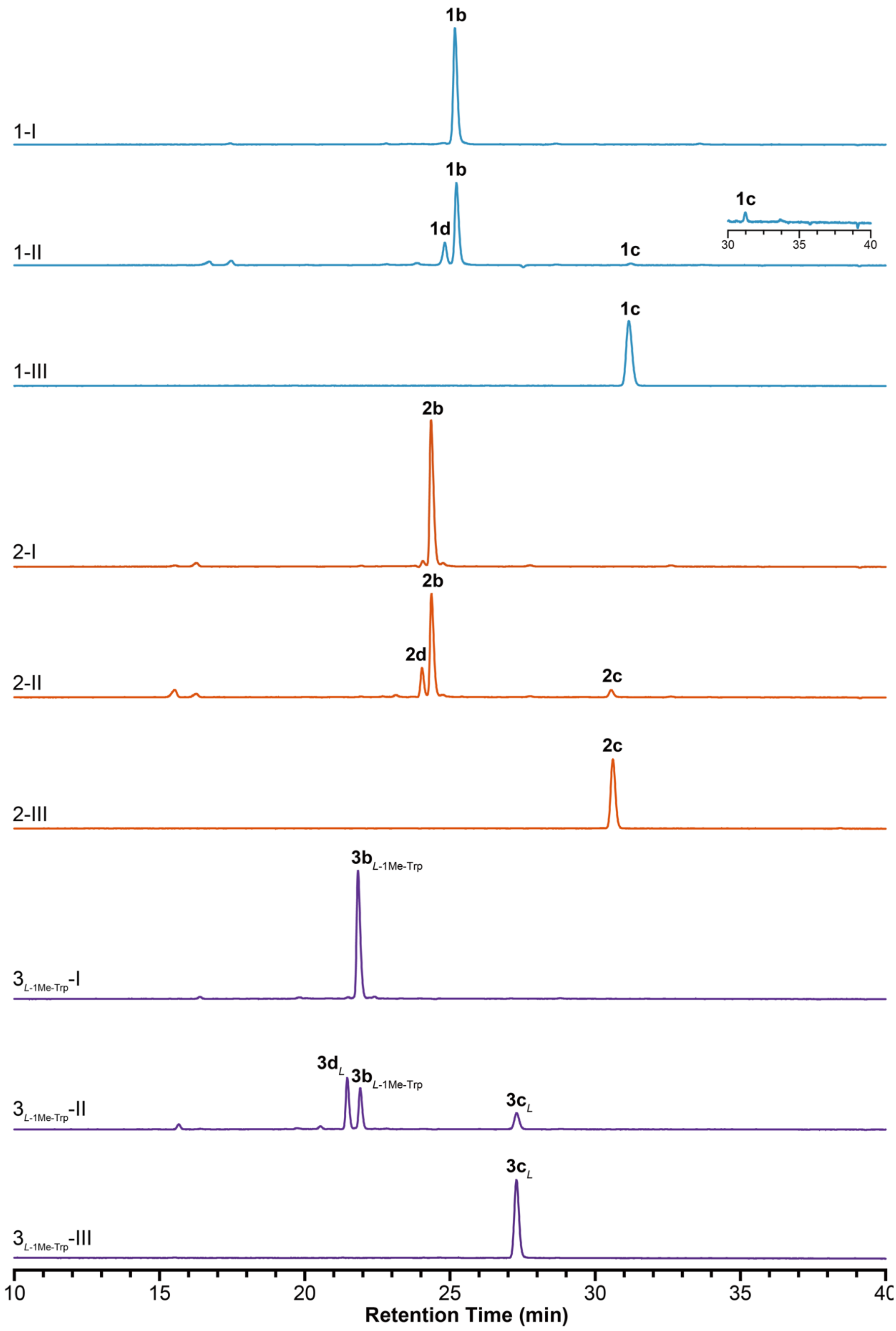

Supplementary Figure 16. HPLC analysis of RufT-TE *in vitro* bioassay of peptides 1, 2 and 3$_{L\text{-1Me-Trp}}$. Cylization and hydrolysis of SNAC peptide **b** catalyzed by His$_6$-SUMO-RufT-TE to produce cyclic peptide **c** and hydrolyzed peptide **d**. Chromatograms (I) is negative control of RufT-TE bioassay performed with inactivated enzyme. Chromatograms (II) is the enzymatic macrocyclization catalyzed by RufT-TE. Chromatograms (III) are the cyclic peptide synthesized by chemical macrocyclization. All UV-vis traces were recorded at 355 nm. All reactions contain SNAC peptide **b** (50 µM), His$_6$-SUMO-RufT-TE (12.5 µM) and 5% DMSO in Tris Buffer (25 mM, 300 mM NaCl, pH 8). And incubated at 30 ºC for 3 h with shaking 800 rpm.

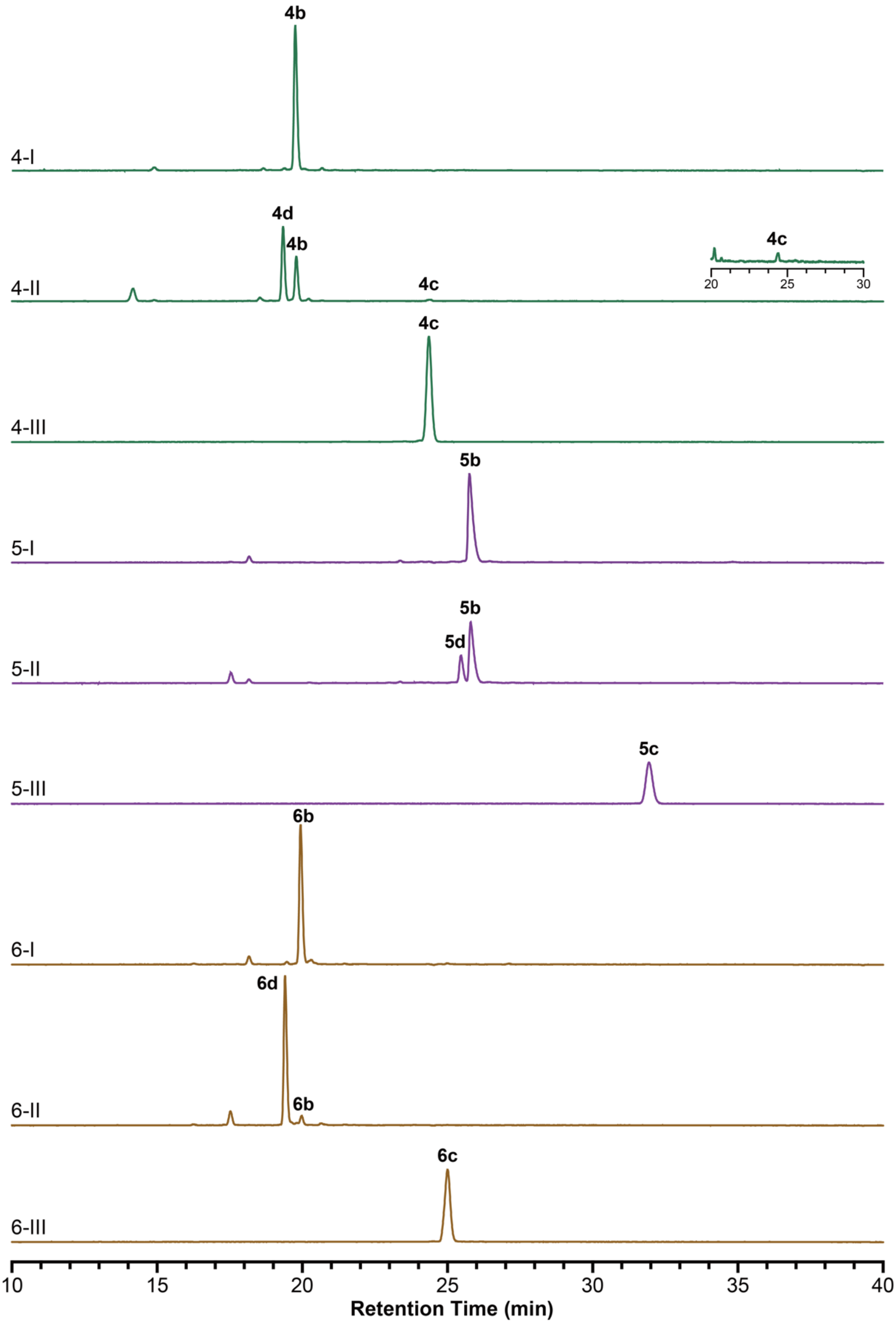

Supplementary Figure 17. HPLC analysis of RufT-TE *in vitro* bioassay of peptides 4, 5 and 6. Cylization and hydrolysis of SNAC peptide **b** catalyzed by His$_6$-SUMO-RufT-TE to produce cyclic peptide **c** and hydrolyzed peptide **d**. Chromatograms (I) is negative control of RufT-TE bioassay performed with thermally inactivated enzyme. Chromatograms (II) is the enzymatic macrocyclization catalyzed by RufT-TE. Chromatograms (III) is the cyclic peptide synthesized by chemical macrocyclization. All UV-vis traces were recorded at 355 nm. All reactions contain SNAC peptide **b** (50 µM), His$_6$-SUMO-RufT-TE (12.5 µM) and 5% DMSO in Tris Buffer (25 mM, 300 mM NaCl, pH 8). And incubated at 30 ºC for 3 h with shaking 800 rpm.

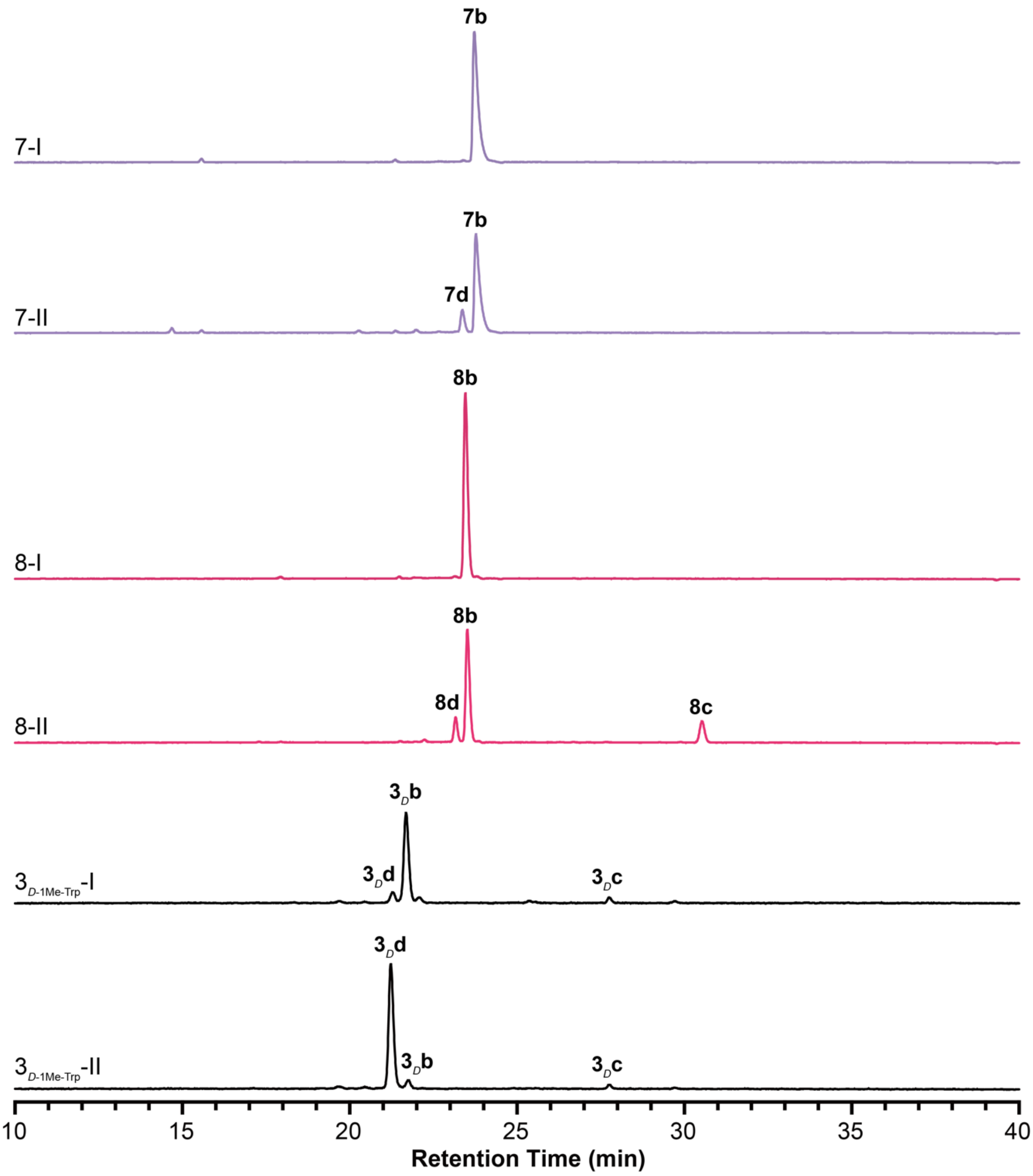

Supplementary Figure 18. HPLC analysis of RufT-TE *in vitro* bioassay of peptides 7, 8 and $3_{D\text{-}1Me\text{-}Trp}$. Cylization and hydrolysis of SNAC peptide **b** catalyzed by His_6-SUMO-RufT-TE to produce cyclic peptide **c** and hydrolyzed peptide **d**. Chromatograms (I) is negative control of RufT-TE bioassay performed with thermally inactivated enzyme. Chromatograms (II) is the enzymatic macrocyclization catalyzed by RufT-TE. All UV-vis traces were recorded at 355 nm. All reactions contain SNAC peptide **b** (50 µM), His_6-SUMO-RufT-TE (12.5 µM) and 5% DMSO in Tris Buffer (25 mM, 300 mM NaCl, pH 8), incubated at 30 ºC for 3 h with shaking 800 rpm.

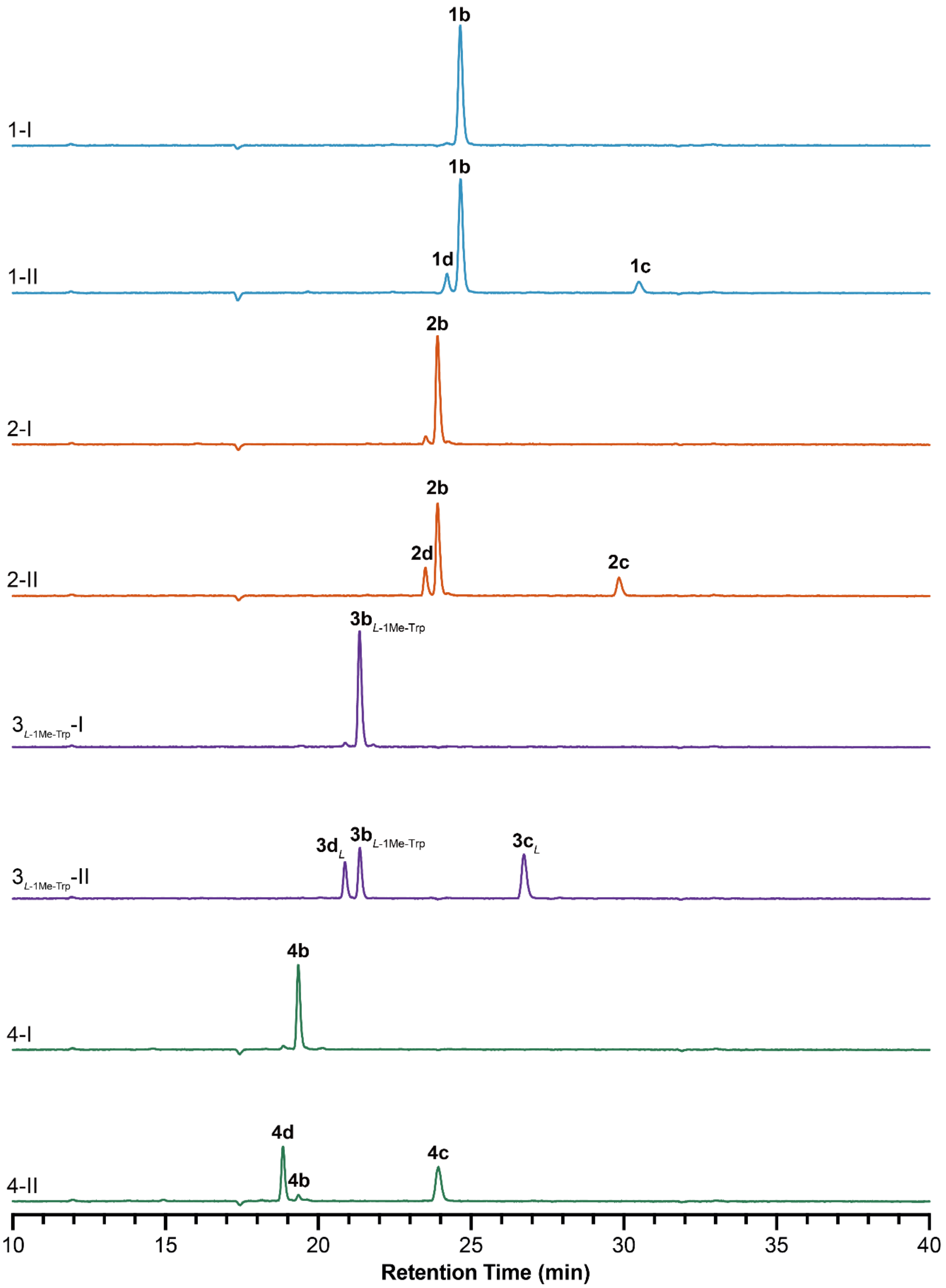

Supplementary Figure 19. HPLC analysis of RufT-PCP-TE *in vitro* bioassay of peptides 1, 2, 3$_{L\text{-1Me-Trp}}$ and 4. Cylization and hydrolysis of SNAC peptide **b** catalyzed by His$_6$-RufT-PCP-TE to produce cyclic peptide **c** and hydrolyzed peptide **d**. Chromatograms (I) is negative control of RufT-PCP-TE bioassay performed with inactivated enzyme. Chromatograms (II) is the enzymatic macrocyclization catalyzed by RufT-PCP-TE. All UV-vis traces were recorded at 355 nm. All reactions contain SNAC peptide **b** (50 µM), His$_6$-RufT-PCP-TE (12.5 µM) and 5% DMSO in Tris Buffer (25 mM, 300 mM NaCl, pH 8). And incubated at 30 ºC for 3 h with shaking 800 rpm.

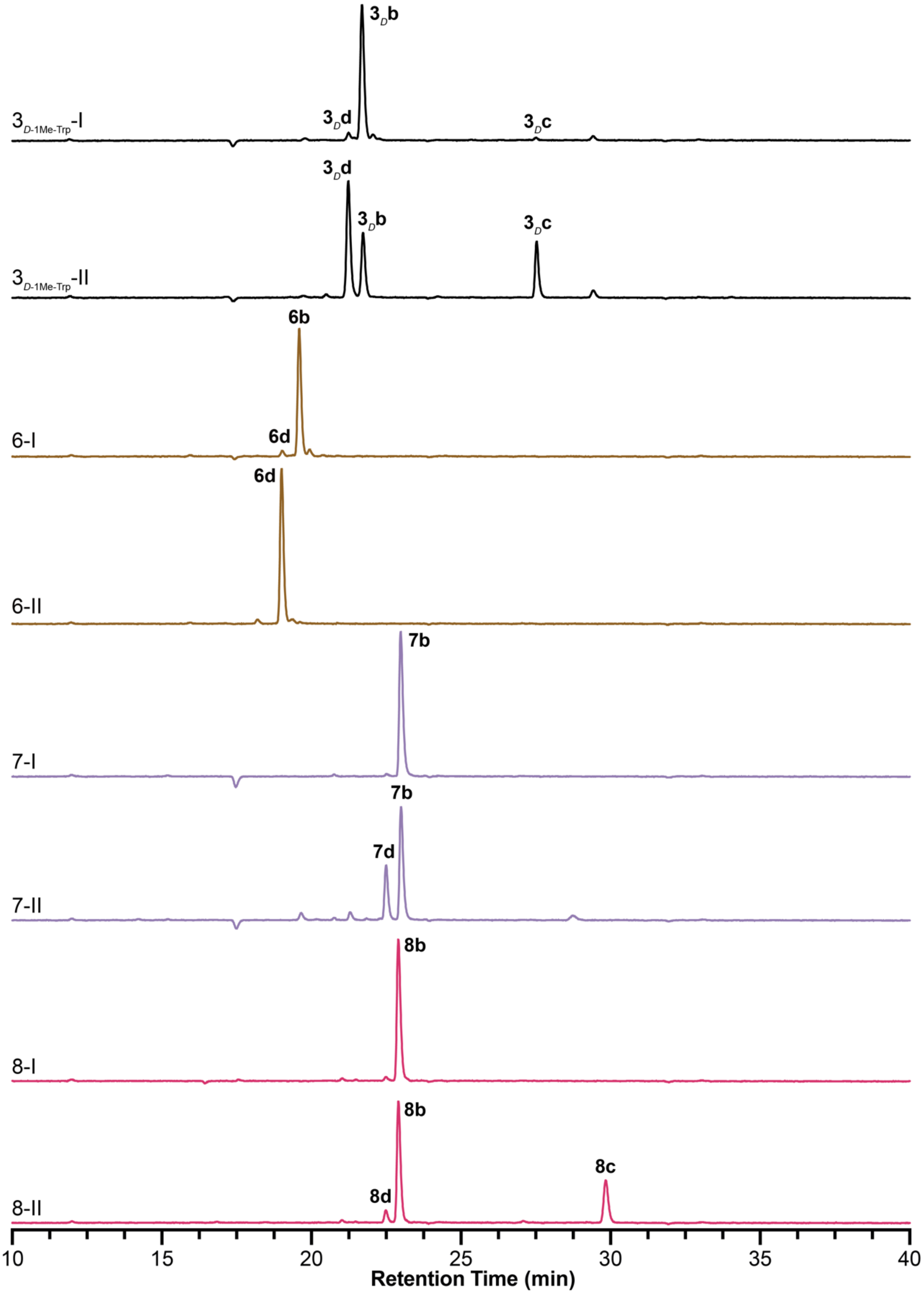

Supplementary Figure 20. HPLC analysis of RufT-PCP-TE *in vitro* bioassay of peptides $3_{D\text{-1Me-Trp}}$, 6, 7 and 8. Cylization and hydrolysis of SNAC peptide **b** catalyzed by His_6-RufT-PCP-TE to produce cyclic peptide **c** and hydrolyzed peptide **d**. Chromatograms (I) is negative control of RufT-PCP-TE bioassay performed with inactivated enzyme. Chromatograms (II) is the enzymatic macrocyclization catalyzed by RufT-PCP-TE. All UV-vis traces were recorded at 355 nm. All reactions contain SNAC peptide **b** (50 µM), His_6-RufT PCP-TE (12.5 µM) and 5% DMSO in Tris Buffer (25 mM, 300 mM NaCl, pH 8). And incubated at 30 ºC for 3 h with shaking 800 rpm.

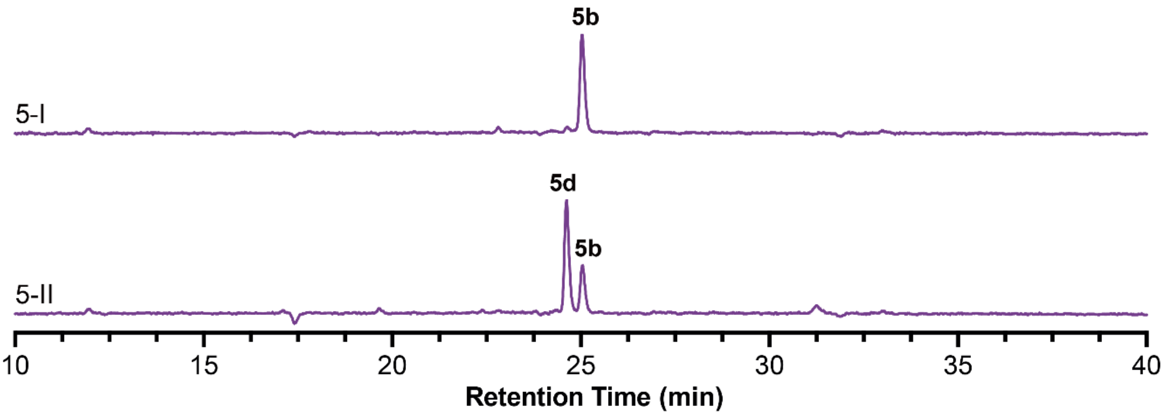

Supplementary Figure 21. HPLC analysis of RufT PCP-TE *in vitro* bioassay of peptide 5. Cylization and hydrolysis of SNAC peptide **b** catalyzed by His_6-RufT-PCP-TE to produce cyclic peptide **c** and hydrolyzed peptide **d**. Chromatograms (I) is negative control of RufT-PCP-TE bioassay performed with inactivated enzyme. Chromatograms (II) is the enzymatic macrocyclization catalyzed by RufT-PCP-TE. All UV-vis traces were recorded at 355 nm. All reactions contain SNAC peptide **b** (50 µM), His_6-RufT-PCP-TE (12.5 µM) and 5% DMSO in Tris Buffer (25 mM, 300 mM NaCl, pH 8). And incubated at 30 ºC for 3 h with shaking 800 rpm.

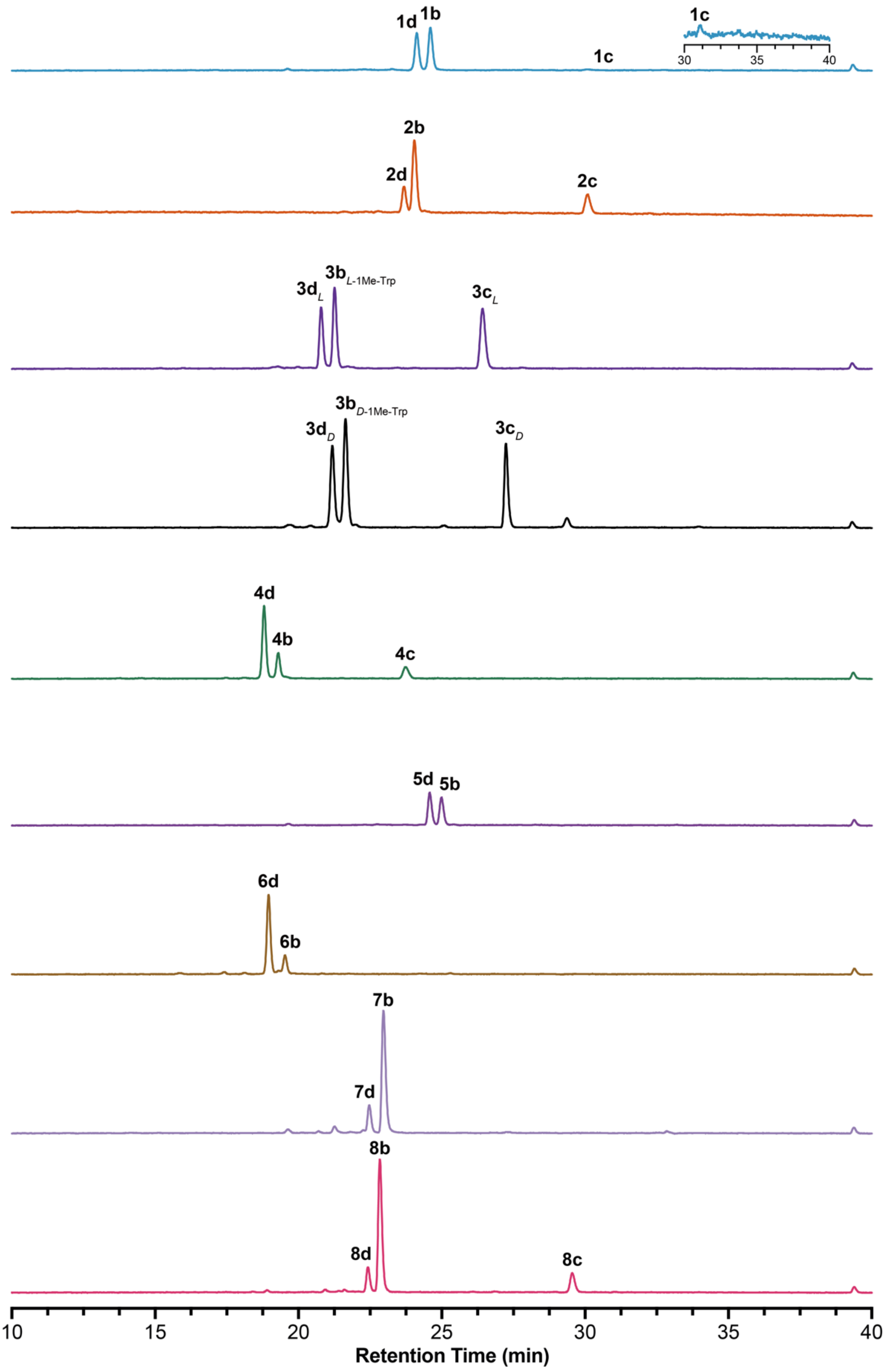

Supplementary Figure 22. HPLC analysis of RufT-TE S105C *in vitro* bioassay of peptides 1 to 8. Cylization and hydrolysis of SNAC peptide **b** catalyzed by His_6-SUMO-RufT-TE S105C to produce cyclic peptide **c** and hydrolyzed peptide **d**. Chromatograms is the enzymatic macrocyclization catalyzed by His_6-SUMO-RufT-TE S105C. All UV-vis traces were recorded at 355 nm. All reactions contain SNAC peptide **b** (50 µM), His_6-SUMO-RufT-TE S105C (12.5 µM) and 5% DMSO in Tris Buffer (25 mM, 300 mM NaCl, pH 8). And incubated at 30 ºC for 3 h with shaking 800 rpm.

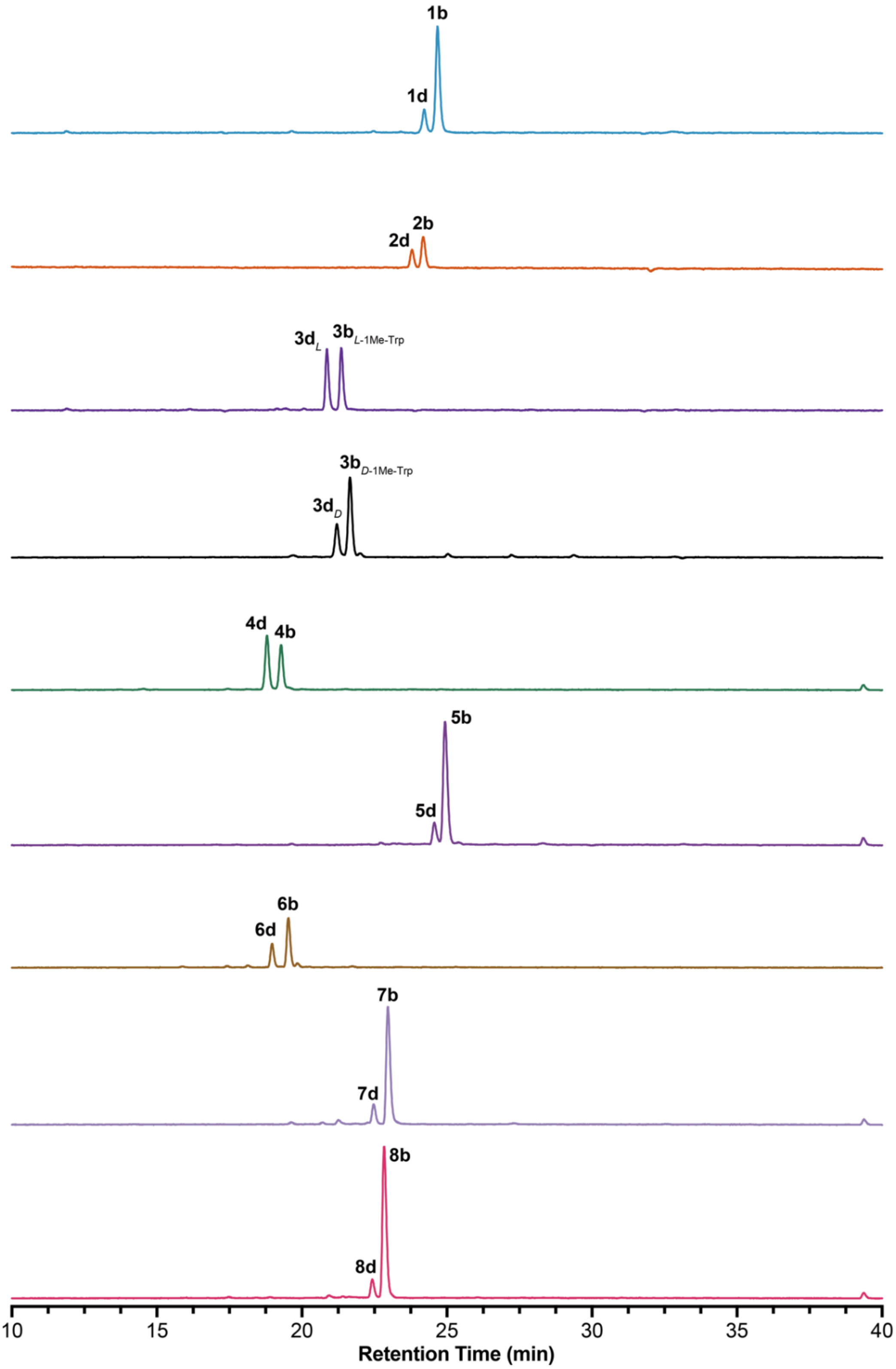

Supplementary Figure 23. HPLC analysis of RufT-TE S105A *in vitro* bioassay of peptides 1 to 8. Cylization and hydrolysis of SNAC peptide **b** catalyzed by His_6-SUMO-RufT-TE S105A to produce cyclic peptide **c** and hydrolyzed peptide **d**. Chromatograms is the enzymatic macrocyclization catalyzed by His_6-SUMO-RufT-TE S105A. All UV-vis traces were recorded at 355 nm. All reactions contain SNAC peptide **b** (50 µM), His_6-SUMO-RufT-TE S105A (12.5 µM) and 5% DMSO in Tris Buffer (25 mM, 300 mM NaCl, pH 8). And incubated at 30 ºC for 3 h with shaking 800 rpm.

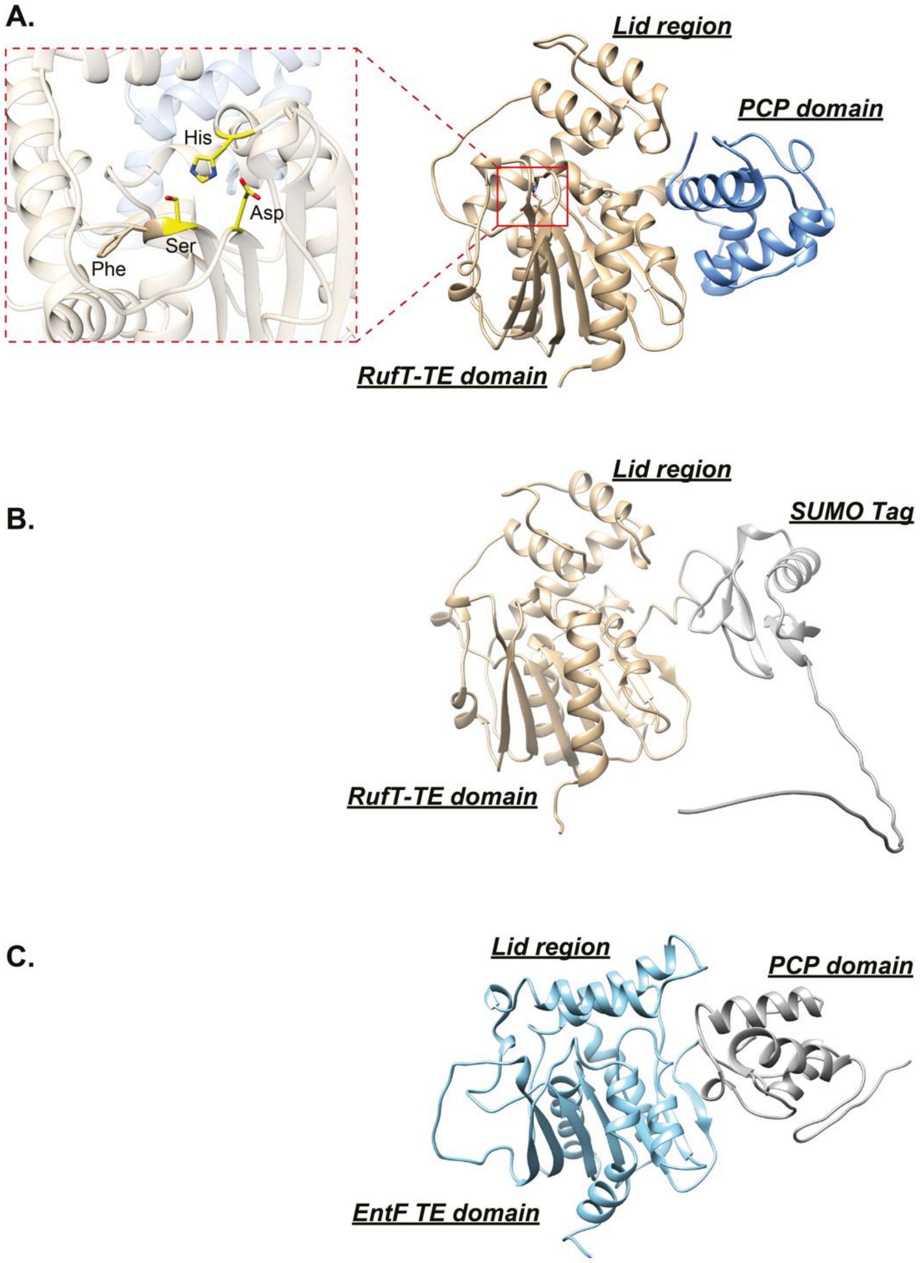

Supplementary Figure 24. Stuctures of Thioesterases. A. Alphafold predicted RufT-PCP-TE domain, catalytic triad are highlighted **B.** Alphafold predicted RufT-TE domain. **C.** NMR characterized EntF-TE (PDB: 2ROQ).[4]

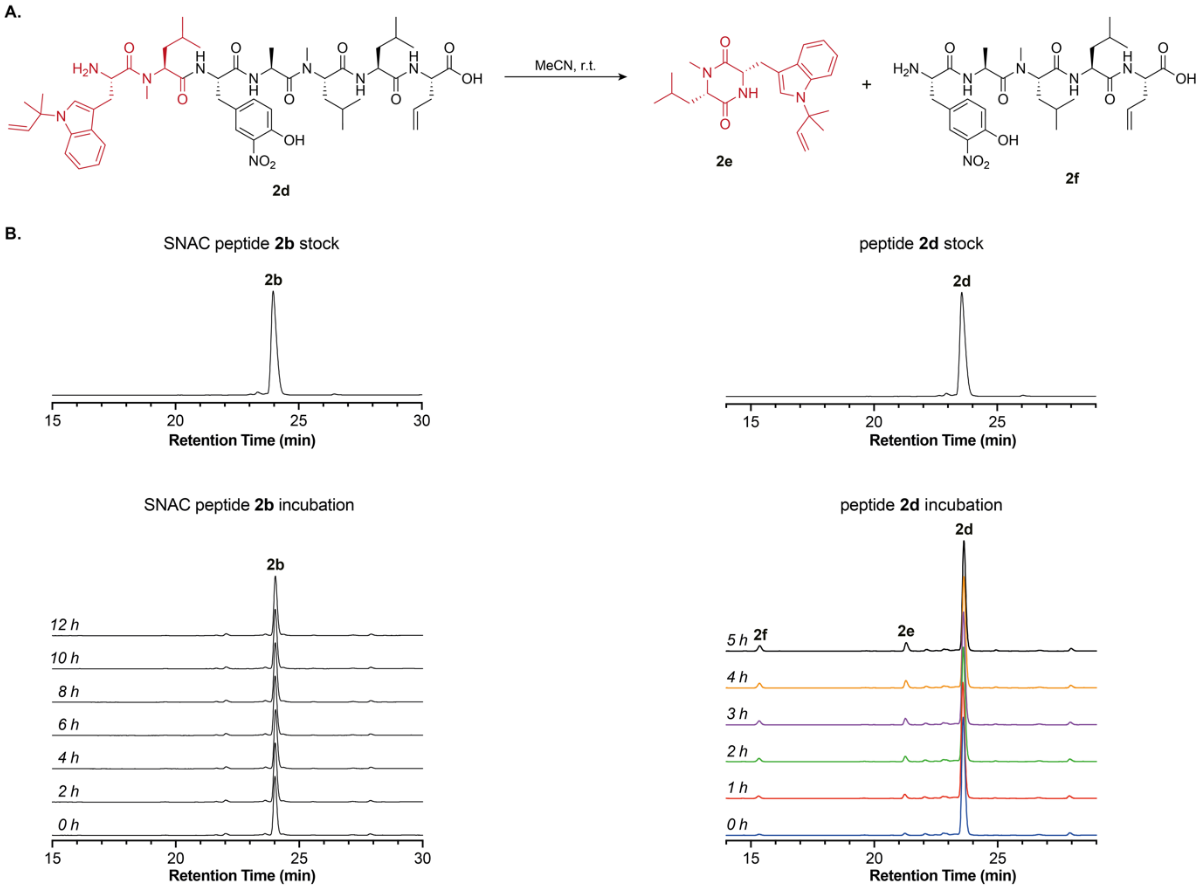

Supplementary Figure 25. Stability test of SNAC peptide 2b and hydrolyzed peptide 2d in acetonitrile. (full timecourses from Fig 4 in main manuscript).

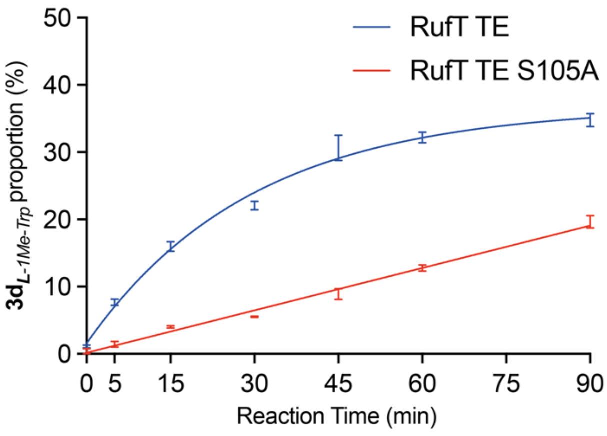

Supplementary Figure 26. Comparison of hydrolysis in reactions of the RufT-TE wild type and RufT-TE S105A mutant in reaction with peptide **3b*L***. Enzymatic cyclization was performed at a total volume of 200 µL. Typically, to SNAC peptide **3b**$_{L\text{-1Me-Trp}}$ (50 µM final conc.) in Tris Buffer (25 mM, 300 mM NaCl, pH = 8) containing 5 % DMSO, RufT-TE or RufT-TE S105A (12.5 µM final conc.) was added to initialize the reaction. The resulting mixture was incubated for various period at 30 ºC with shaking 800 rpm. The reaction was extracted with ethyl acetate (3 x 400 µL). The combined organic layers were dried and redissolved in 40 µL 80% acetonitrile in H_2O. The sample was analyzed via HPLC using Gradient 1.

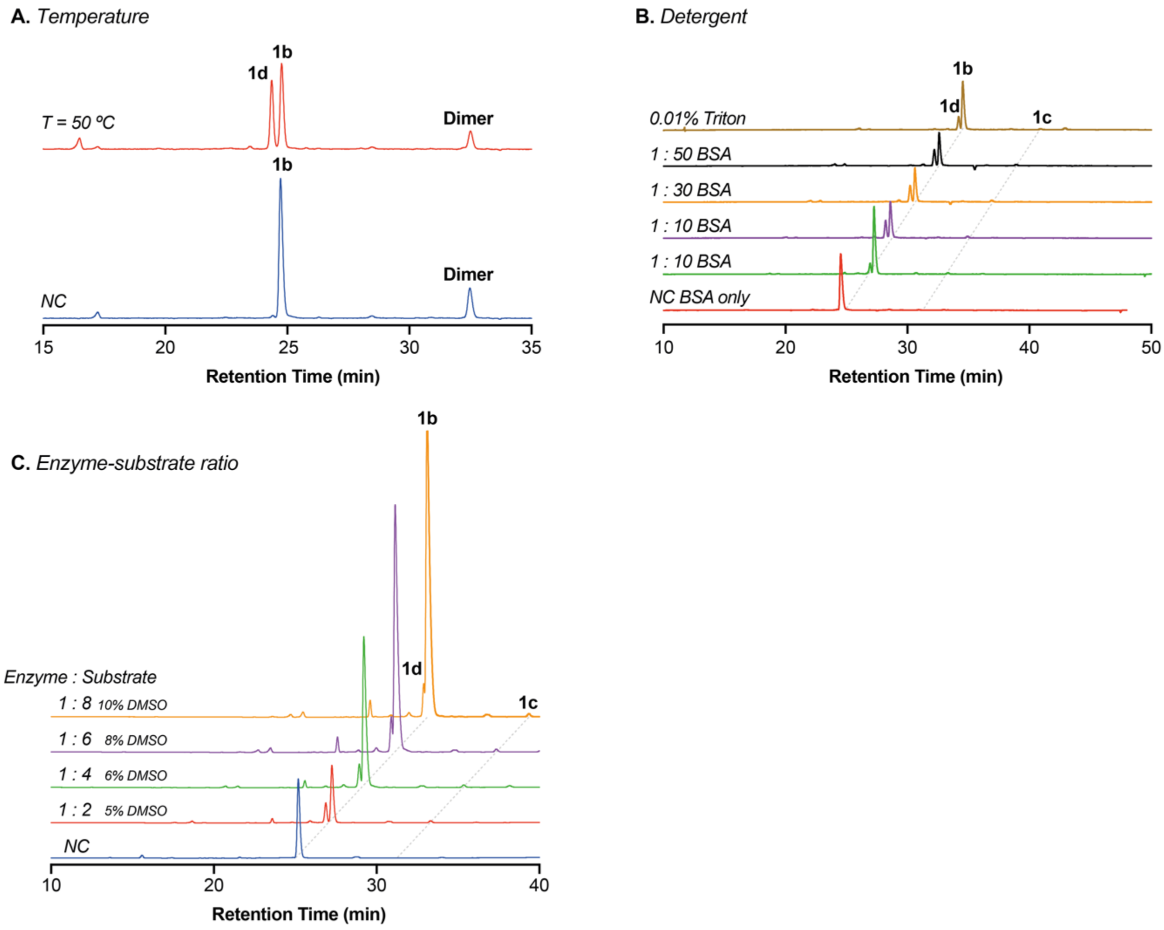

Supplementary Figure S27. HPLC analysis of representative RufT-TE bioassay optimization of substrate 1b (temperature, detergent and enzyme-substrate ratio). ALL bioassays were performed in 25 mM Tris buffer, no added NaCl. General condition: 25 µM enzyme, 50 µM SNAC peptide, 25 mM Tris buffer, pH 8, 3 h, 800 rpm.

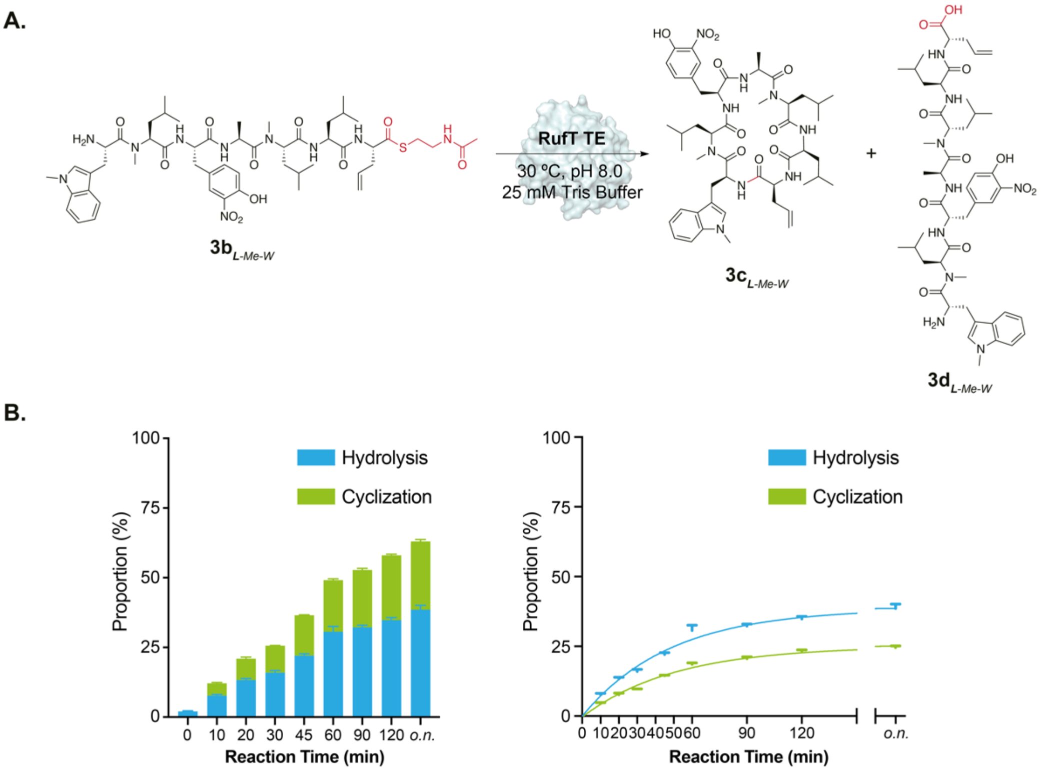

Supplementary Figure S28. RufT-TE *in vitro* bioassay timecourse of peptides 3b$_{L\text{-1Me-Trp}}$**. A.** Cylization and hydrolysis of SNAC peptide **b** catalyzed by His$_6$-SUMO-RufT-TE to produce cyclic peptide **c** and hydrolyzed peptide **d**. All reactions contain SNAC peptide **b** (50 µM), His$_6$-SUMO-RufT TE (25 µM) and 5% DMSO in Tris Buffer (25 mM, pH 8), incubated at 30 ºC with shaking 800 rpm. **B.** Comparison of cyclization and hydrolysis conversion of SNAC peptides **3b**$_{L\text{-1Me-Trp}}$. Conversion was calculated by HPLC monitored at 280 nm, o.n.: overnight.

Supplementary Table 4. HRMS of hydrazide peptide (a), SNAC-peptide (b), enzymatically produced cyclic peptide (c) and hydrolyzed peptide (d). All peptide mass listed here for $[M+H]^+$ and only monoisotopic peaks are listed in the table. All peptide samples were analyzed on LC-HRMS using Gradient 7.

Peptide	Acyl hydrazide-peptide (**a**)			SNAC-peptide (**b**)			Cyclic-peptide (**c**)			Hydrolyzed-peptide (**d**)		
	Calc. (m/z)	Found (m/z)	Error (ppm)	Calc. (m/z)	Found (m/z)	Error (ppm)	Calc. (m/z)	Found (m/z)	Error (ppm)	Calc. (m/z)	Found (m/z)	Error (ppm)
**1**	1044.6246	1044.6235	-1.05	1131.6277	1131.6283	0.53	1012.5872	1012.5898	2.56	1030.5978	1030.5958	-1.94
**2**	1030.6090	1030.6094	0.39	1117.6119	1117.6111	-0.72	998.5715	998.5714	-0.10	1016.5821	1016.5854	3.24
**3*L***	976.5620	976.5613	-0.72	1063.5651	1063.5647	-0.38	944.5245	944.5281	3.81	962.5352	962.5370	1.87
**3*D***	976.5620	976.5637	1.74	1063.5651	1063.5604	-4.40	944.5245	944.5221	-2.54	962.5352	962.5332	-2.08
**4**	936.5307	936.5313	0.64	1023.5338	1023.5331	-0.68	904.4933	904.4977	4.86	922.5038	922.5017	-2.27
**5**	1080.6246	1080.6243	-0.28	1167.6277	1167.6235	-3.60				1066.5978	1066.5962	-1.50
**6**	897.5198	897.5229	3.44	984.5228	984.5215	-1.32				883.4929	883.4932	0.34
**7**	1016.5933	1016.5936	0.30	1103.5963	1103.5941	-1.99				1002.5664	1002.5671	0.7
**8**	1016.5933	1016.5936	0.30	1103.5963	1103.5941	-1.99	984.5558	984.5583	2.54	1002.5664	1002.5671	0.7

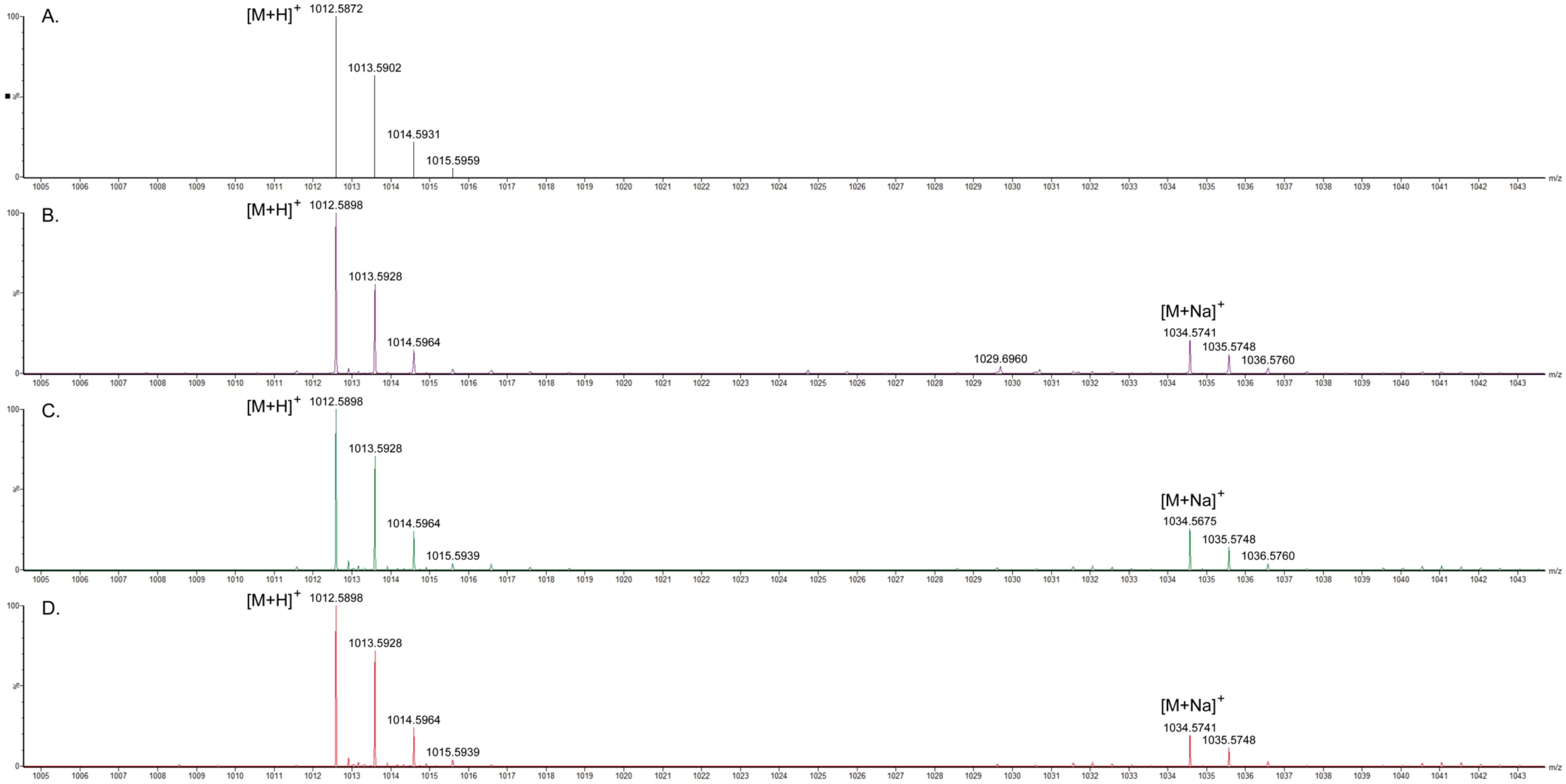

Supplementary Figure 29. HRMS analysis of cyclic peptide 1c rufomycin B. A. Simulated mass spectrum of **1c** rufomycin B. **B.** Mass spectrum of enzymatically cyclized **1c**. **C.** Mass Spectrum of chemically cyclized **1c**. **D.** Mass spectrum of **1c** rufomycin B isolated from bacteria.

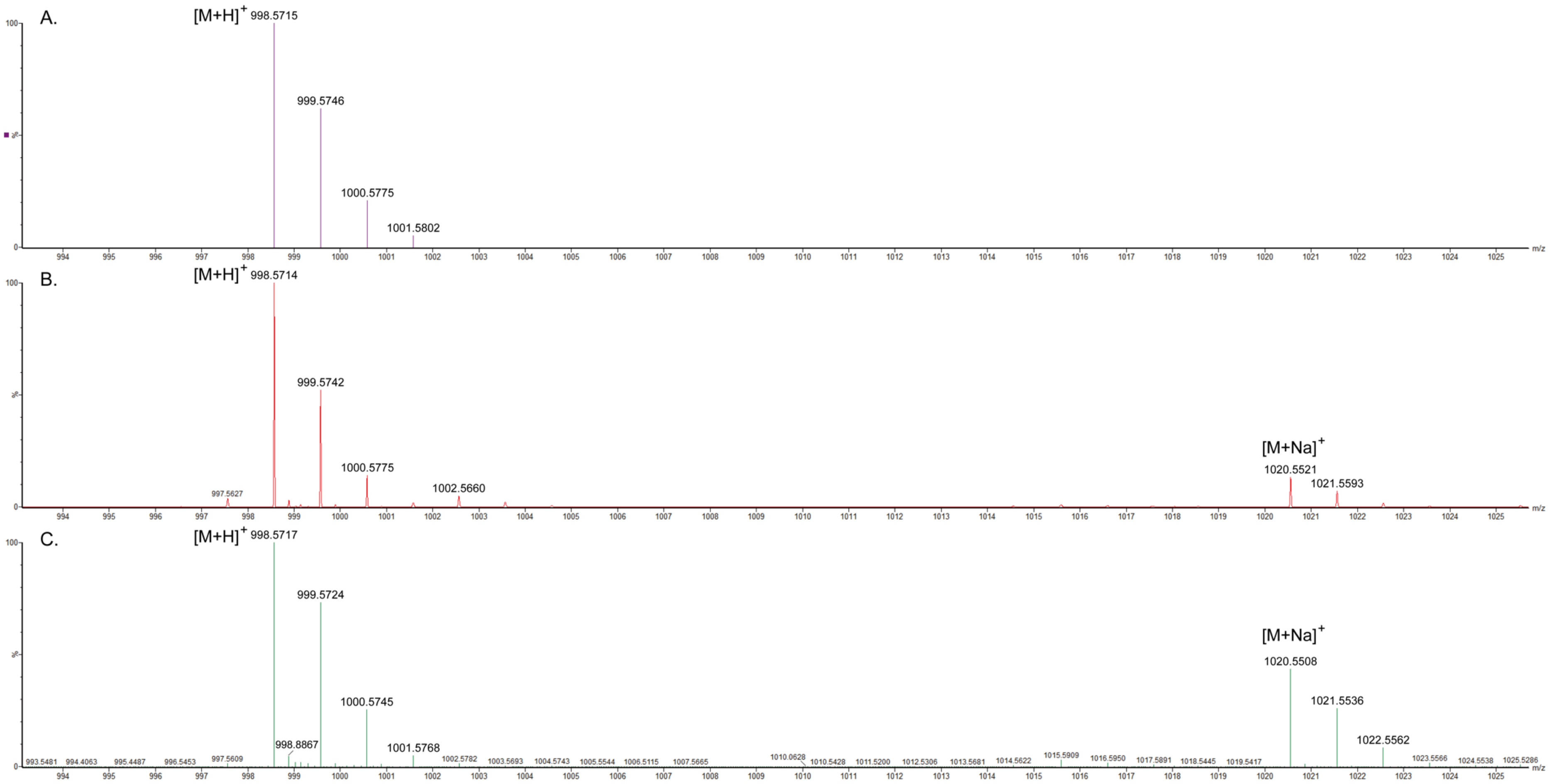

Supplementary Figure 30. HRMS analysis of cyclic peptide 2c. A. Simulated mass spectrum of **2c**. **B.** Mass spectrum of enzymatically cyclized **2c**. **C.** Mass Spectrum of chemically cyclized **2c**.

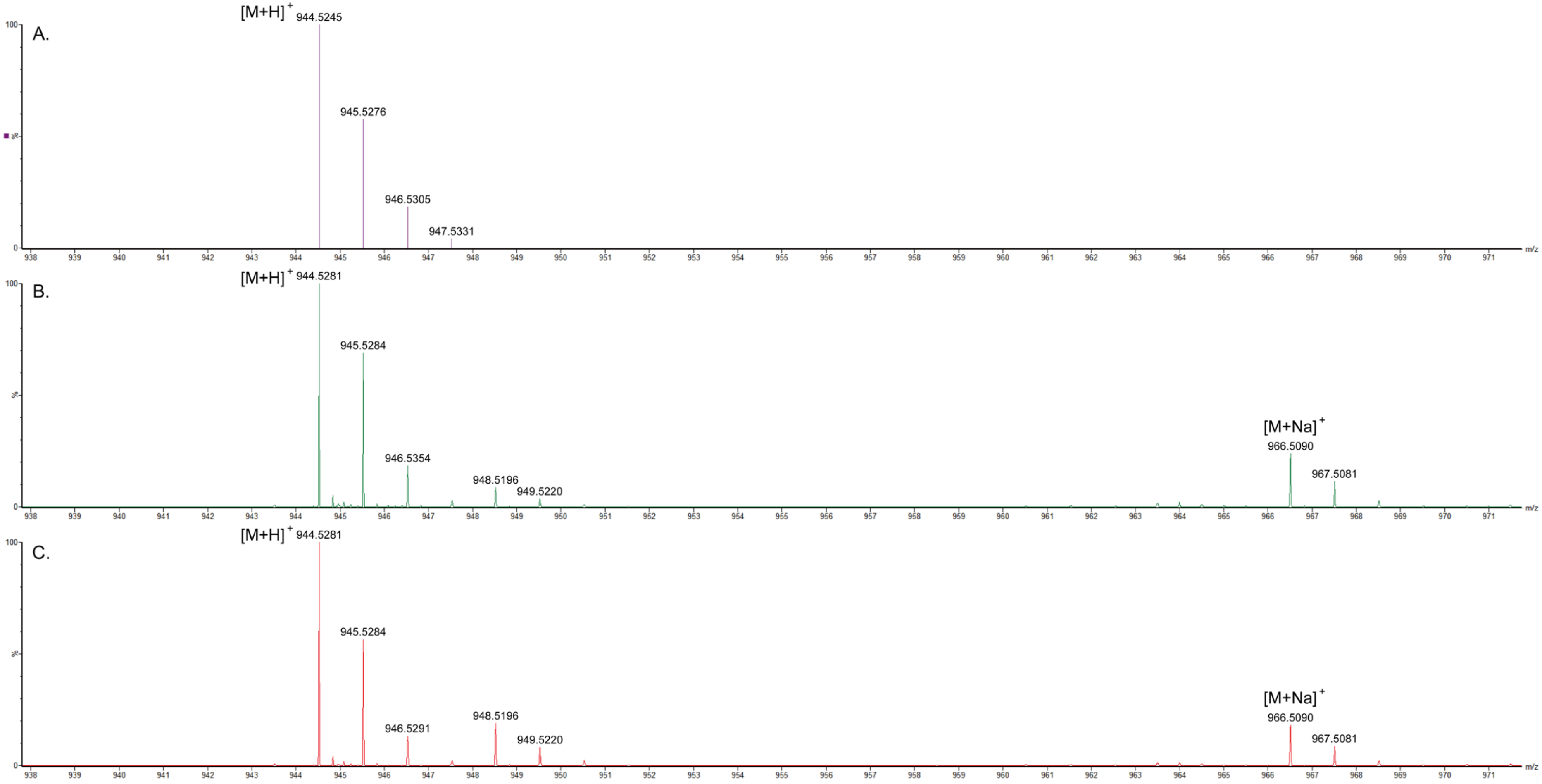

Supplementary Figure 31. HRMS analysis of cyclic peptide 3c$_{L\text{-1Me-Trp}}$**. A.** Simulated mass spectrum of **3c**$_{L\text{-1Me-Trp}}$. **B.** Mass spectrum of enzymatically cyclized **3c**$_{L\text{-1Me-Trp}}$. **C.** Mass Spectrum of chemically cyclized **3c**$_{L\text{-1Me-Trp}}$.

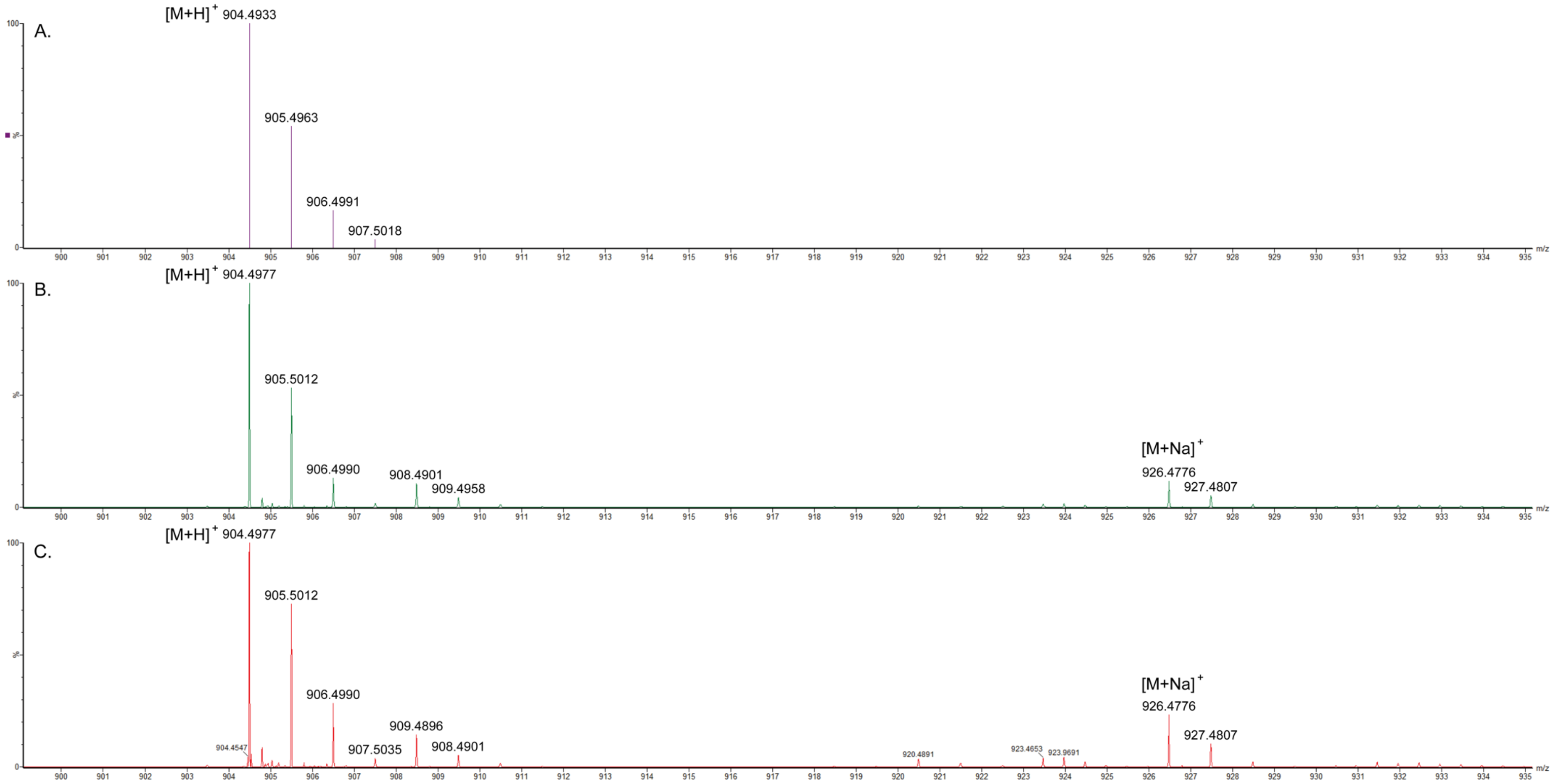

Supplementary Figure 32. HRMS analysis of cyclic peptide 4c. A. Simulated mass spectrum of **4c**. **B.** Mass spectrum of enzymatically cyclized **4c**. **C.** Mass Spectrum of chemically cyclized **4c**.

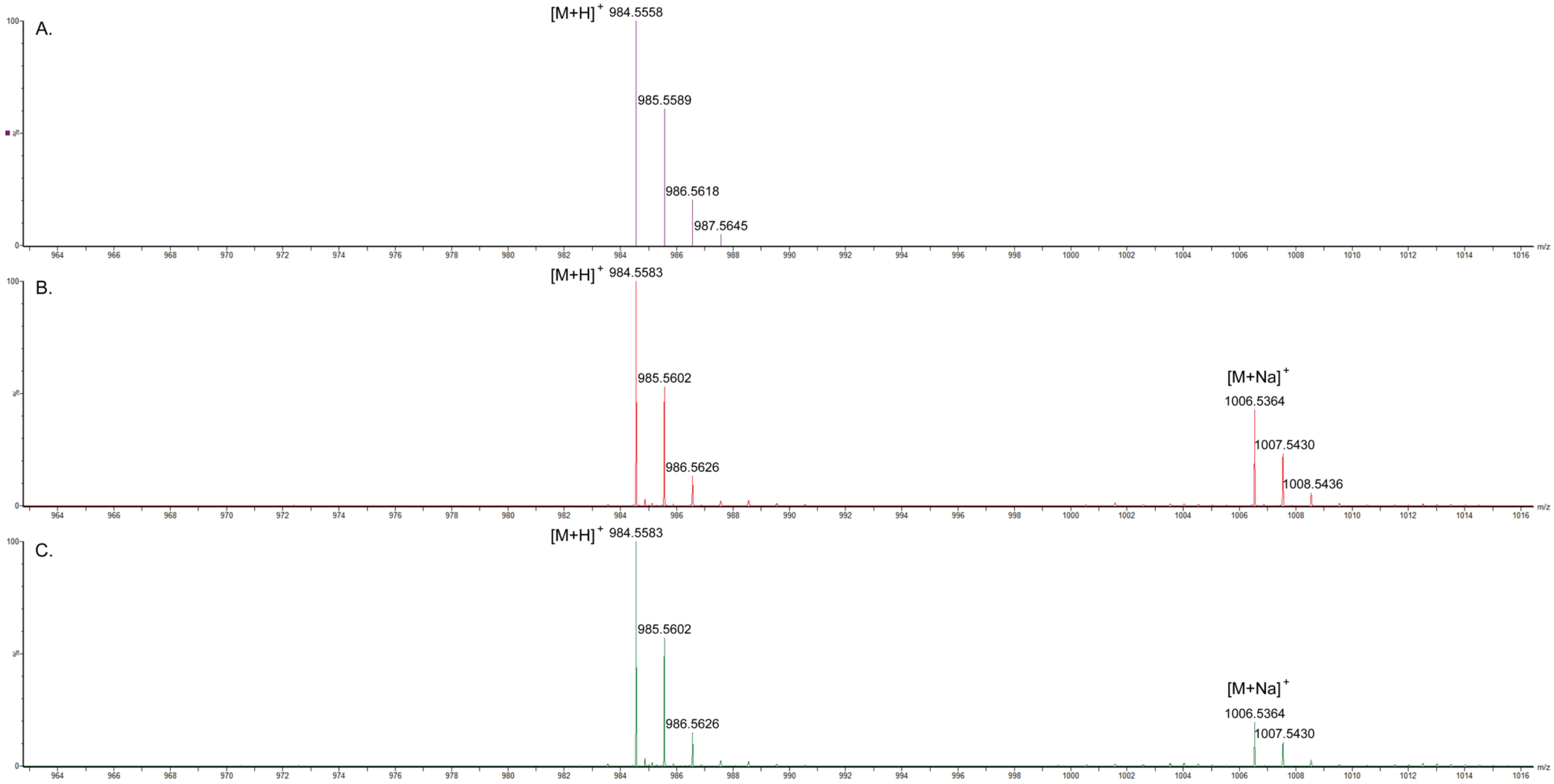

Supplementary Figure 33. HRMS analysis of cyclic peptide 8c. A. Simulated mass spectrum of **8c**. **B.** Mass spectrum of enzymatically cyclized **8c**. **C.** Mass Spectrum of chemically cyclized **8c.**

4. Supplementary Characterization Data

4.1 ¹H and ¹³C NMR spectra

¹H NMR of Fmoc-*N*Me-*L*-Leu-*N*-(1,1-dimethyl-1-allyl)-*L*-Trp-OMe (**10**).

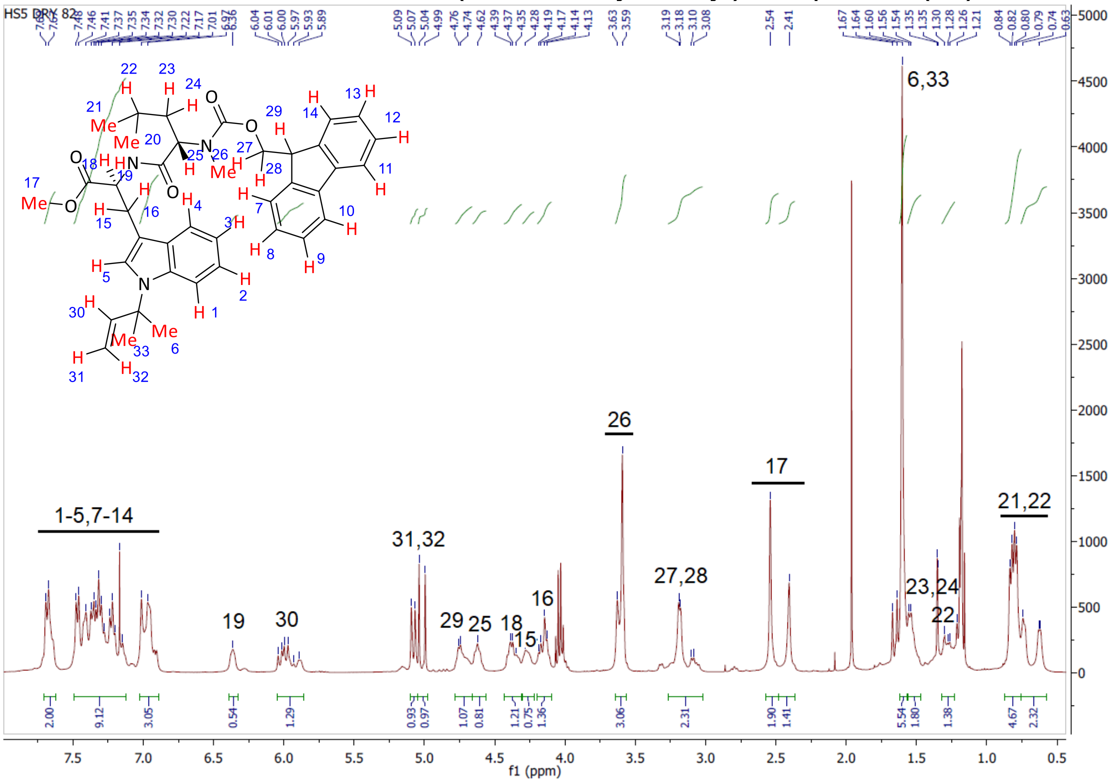

¹³C NMR of Fmoc-NMe-*L*-Leu-*N*-(1,1-dimethyl-1-allyl)-*L*-Trp-OMe (**10**).

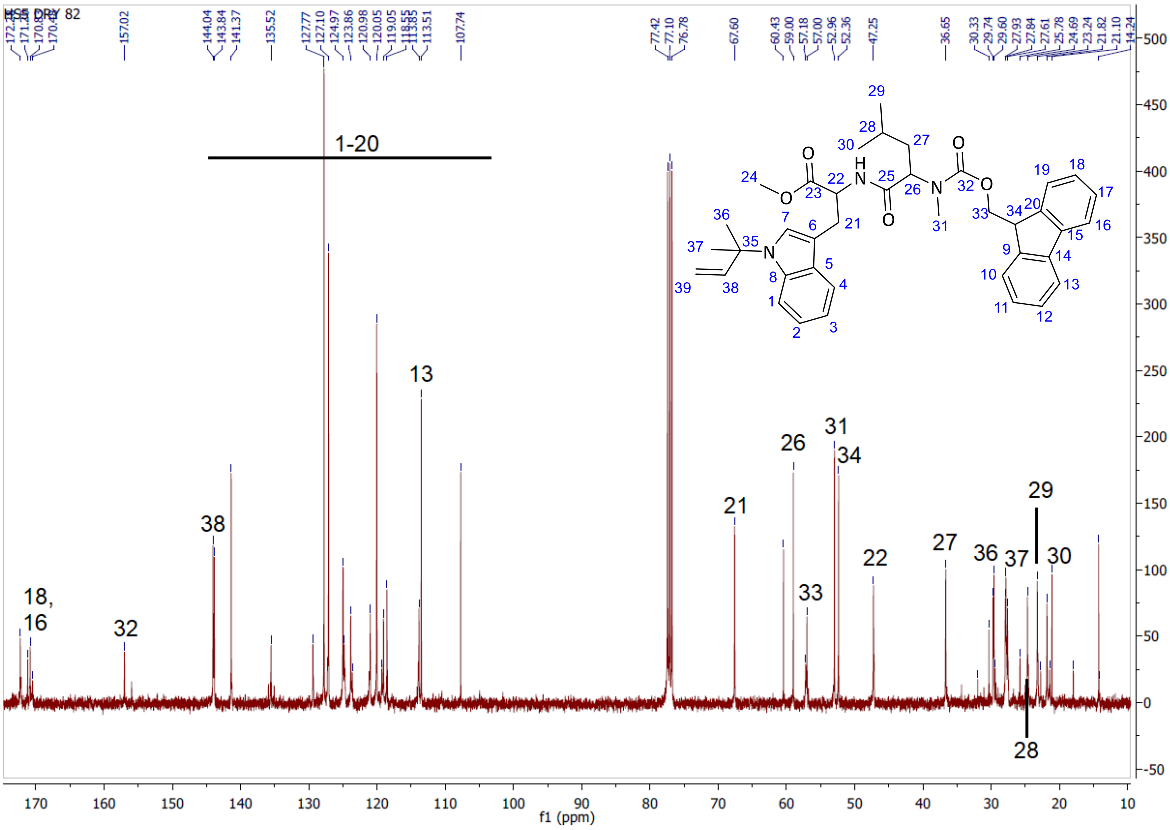

^{1}H NMR of Rufomyazine (**2e**)

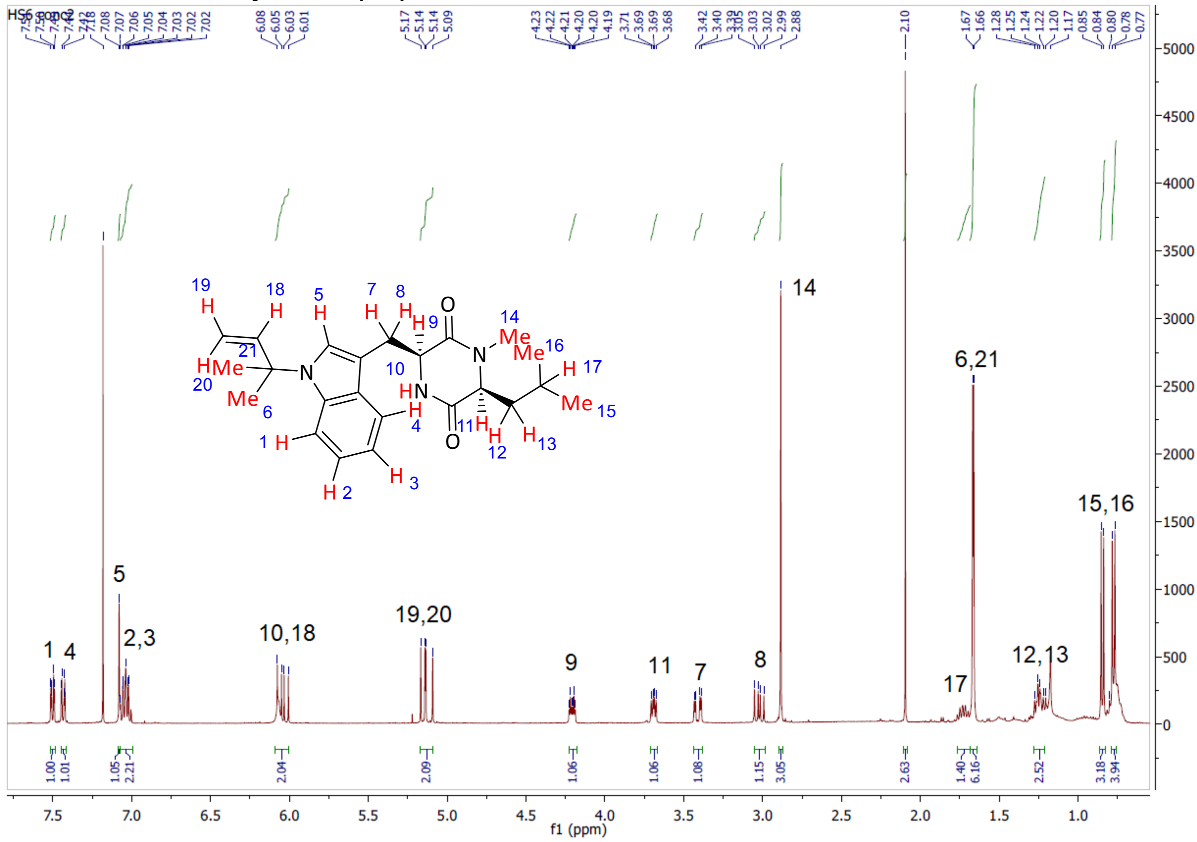

^{13}C NMR of Rufomyazine (**2e**)

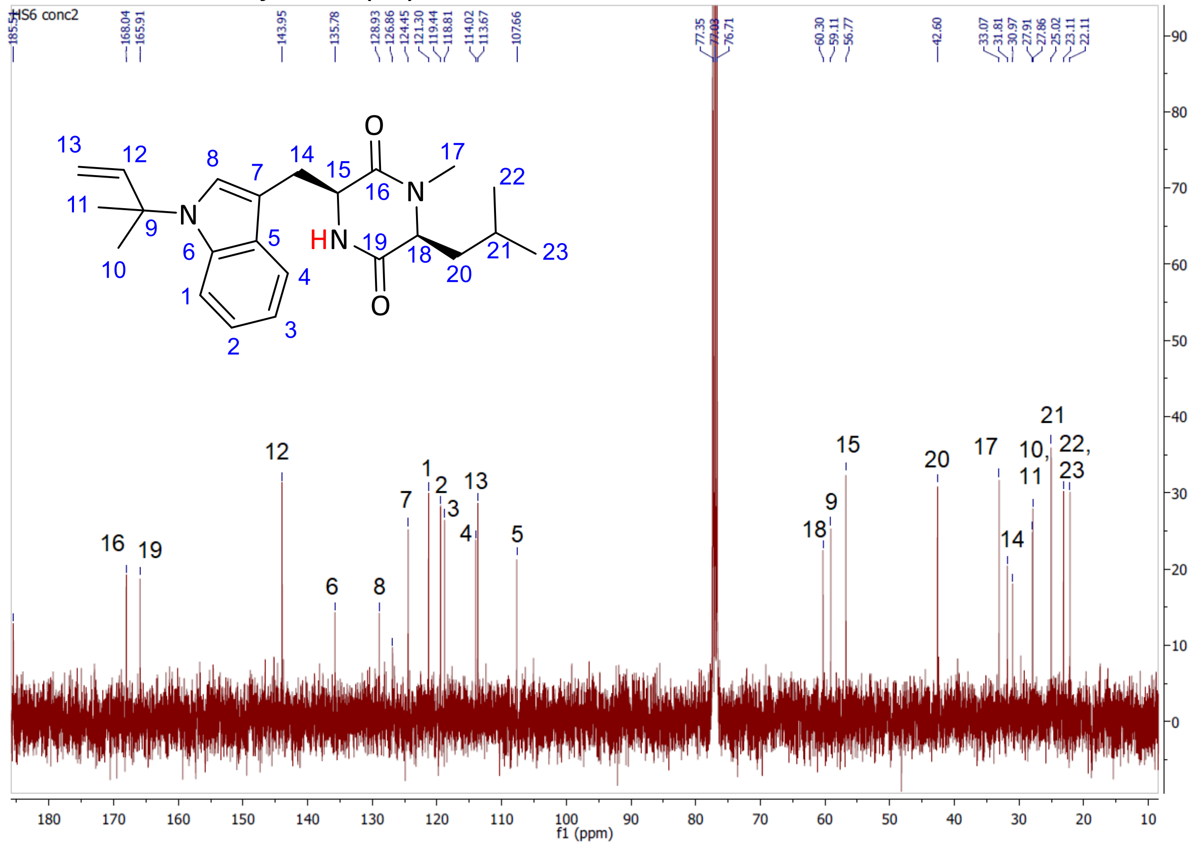

4.2 High Resolution Mass Spectra and Fragmentation of Linear Peptides

HRMS spectra for peptide ***1a*** *: simulated spectrum (top), measured spectrum (below).*

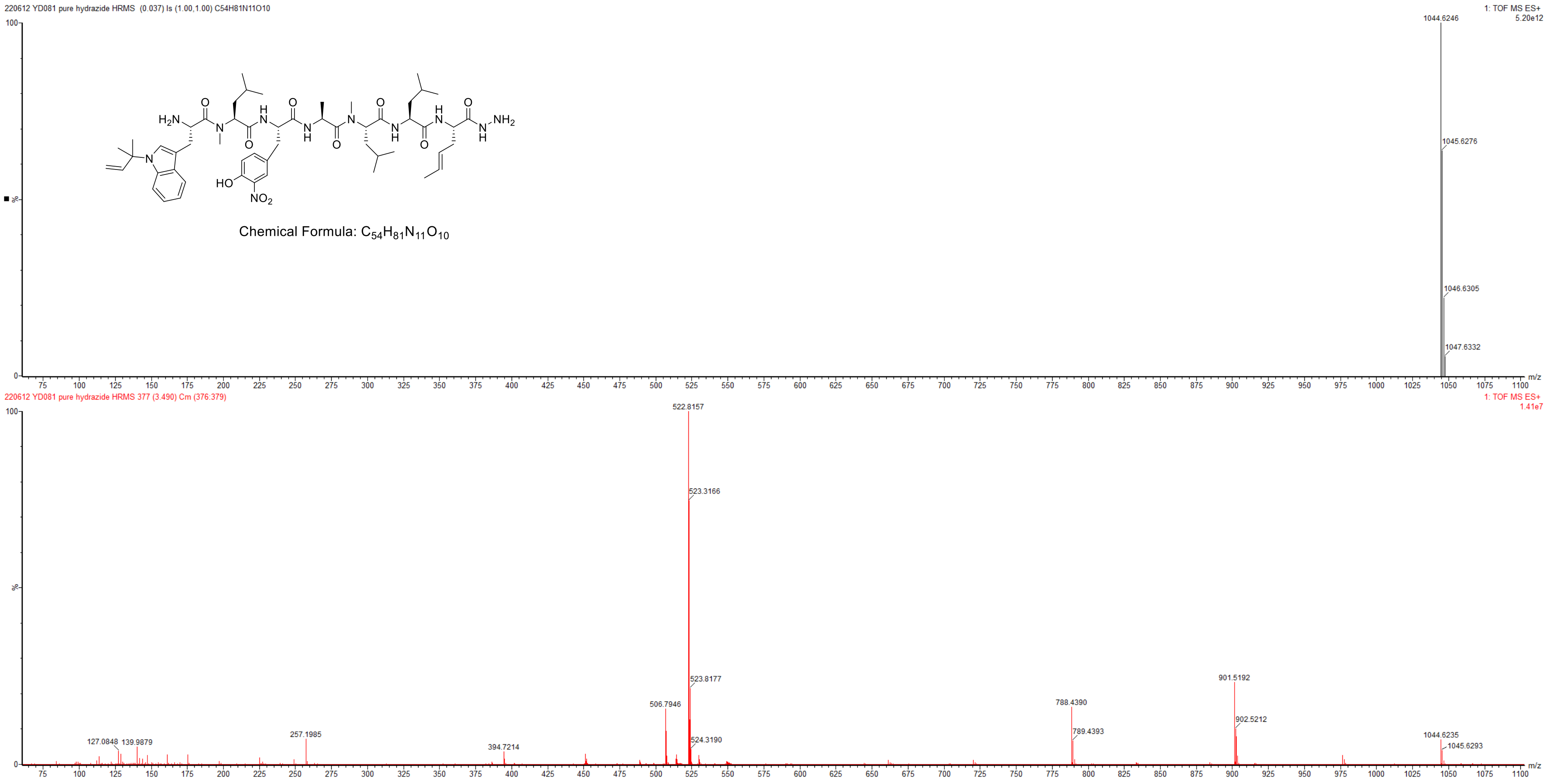

MS^E spectrum for peptide ***1a***

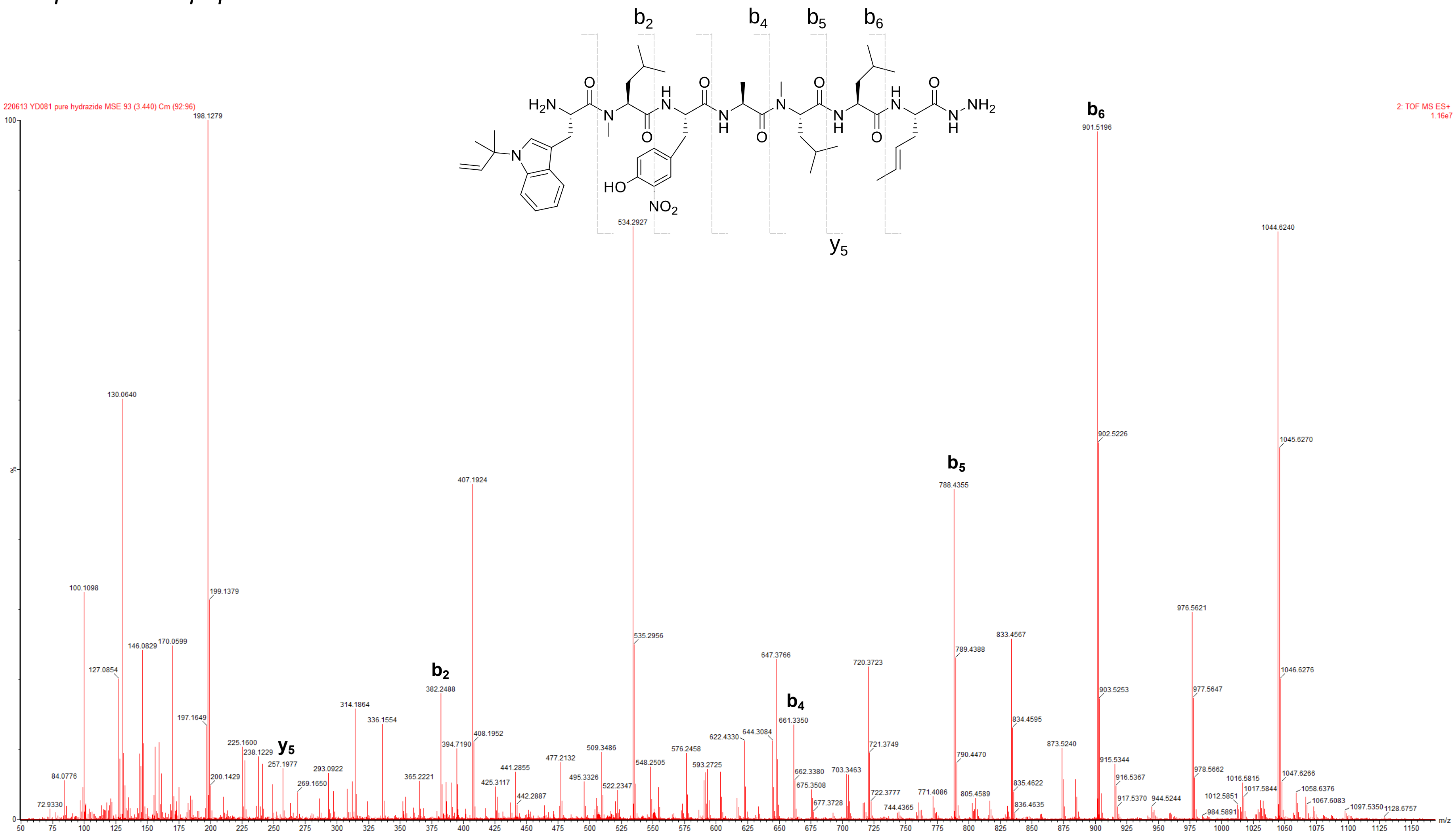

*HRMS spectra for peptide **1b** : simulated spectrum (top), measured spectrum (below).*

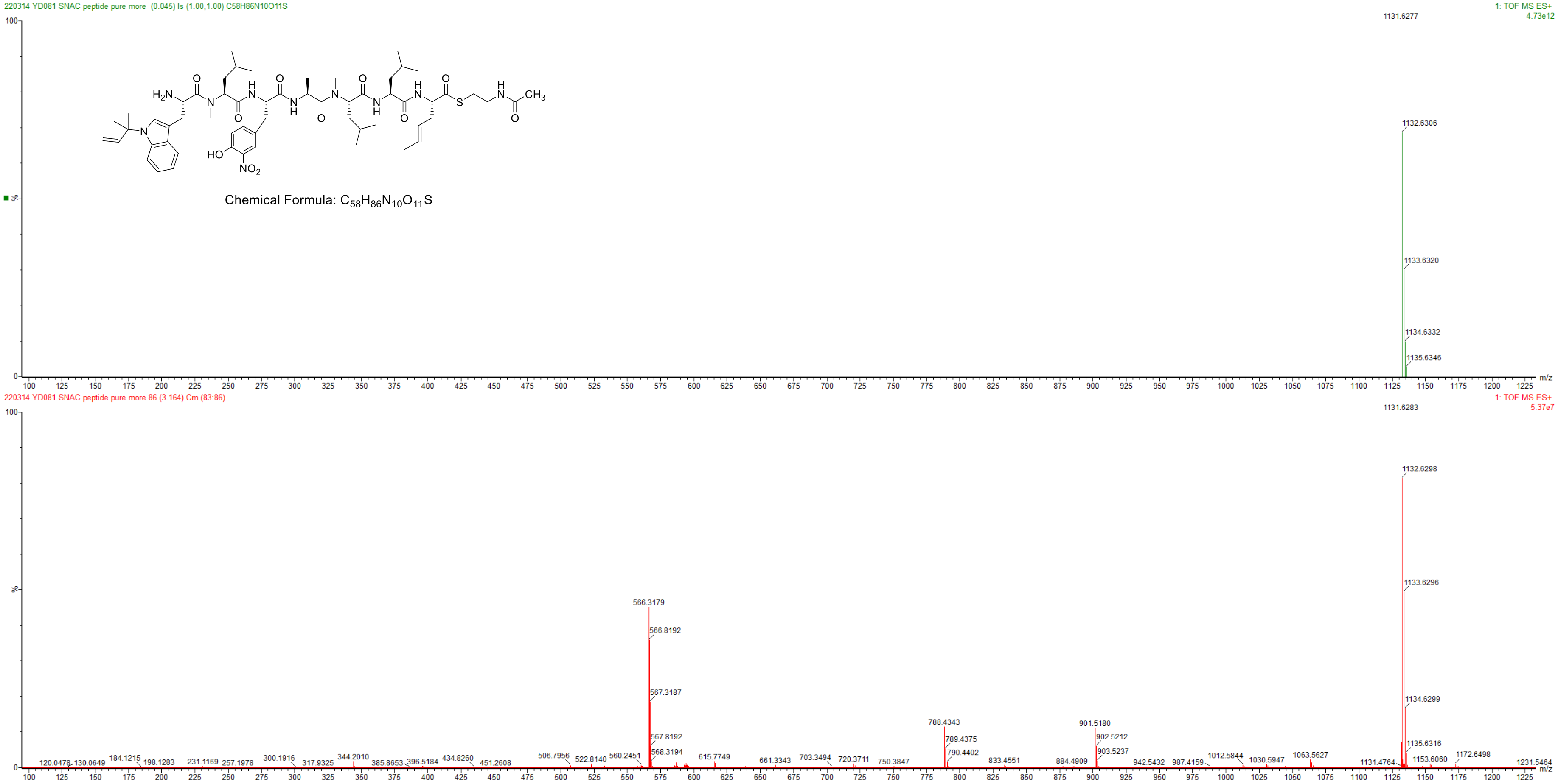

MS^E spectrum for peptide ***1b***

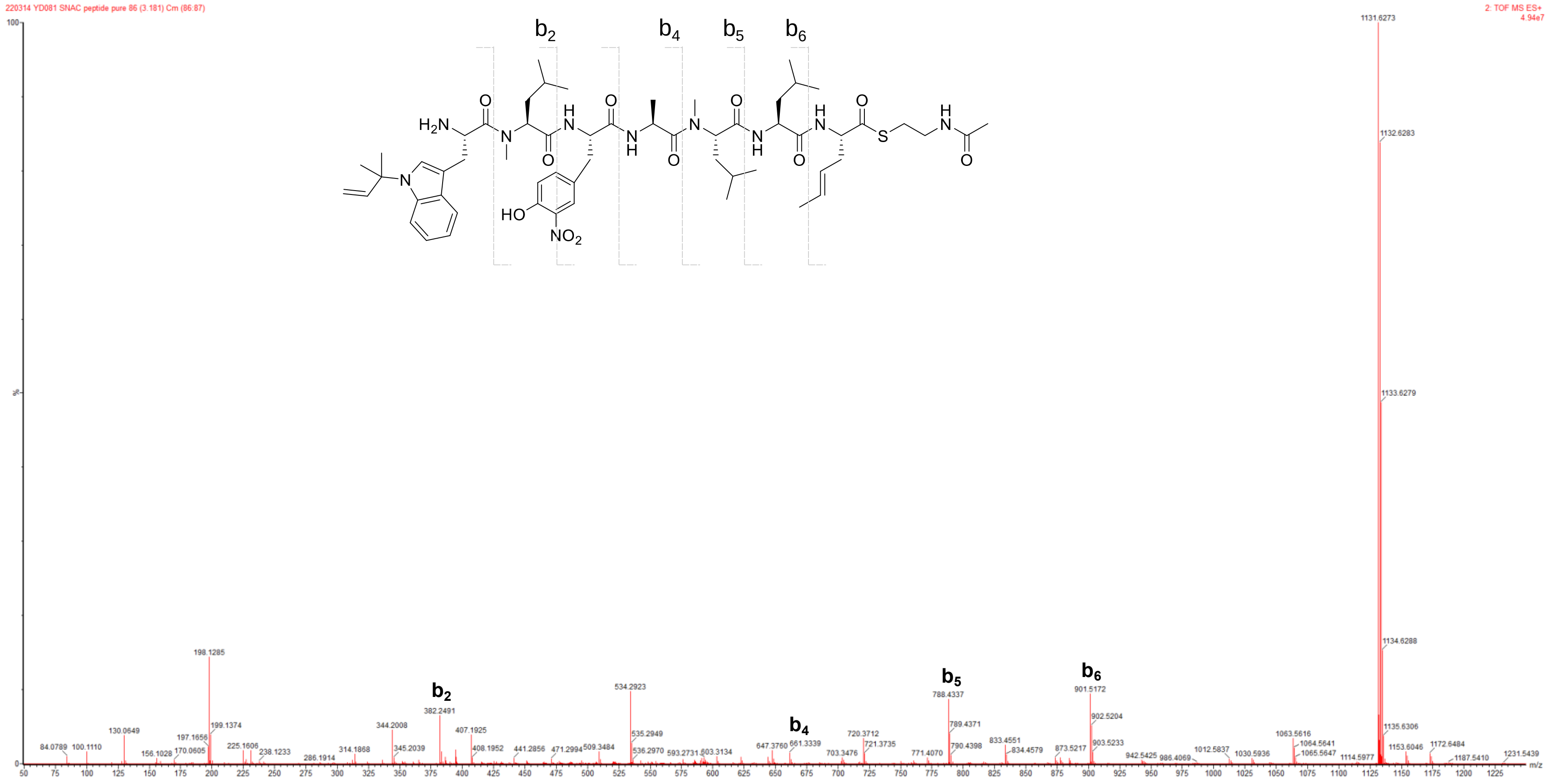

*HRMS spectra for peptide **2a**: simulated spectrum (top), measured spectrum (below).*

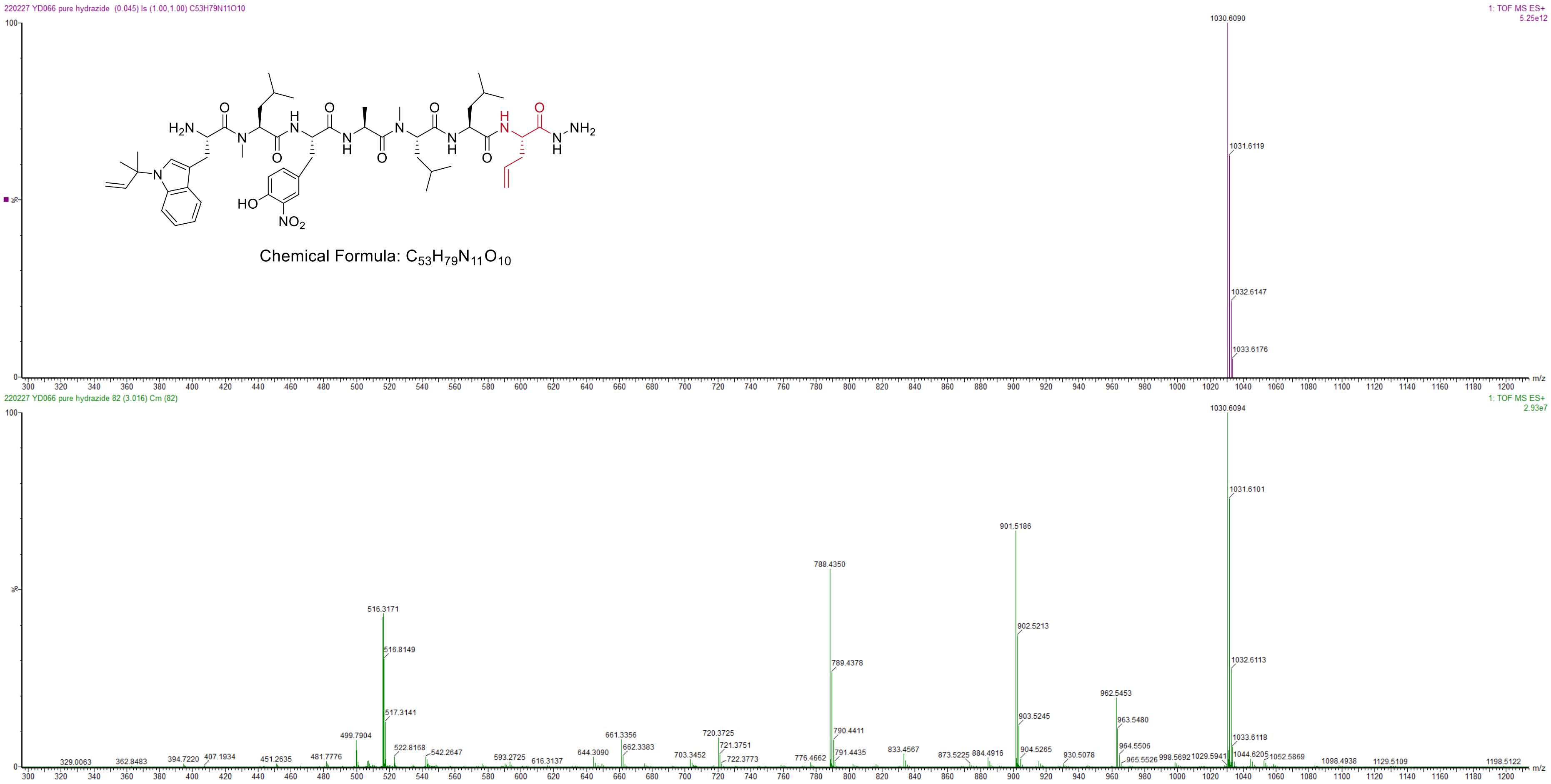

*MS^E spectrum for peptide **2a***

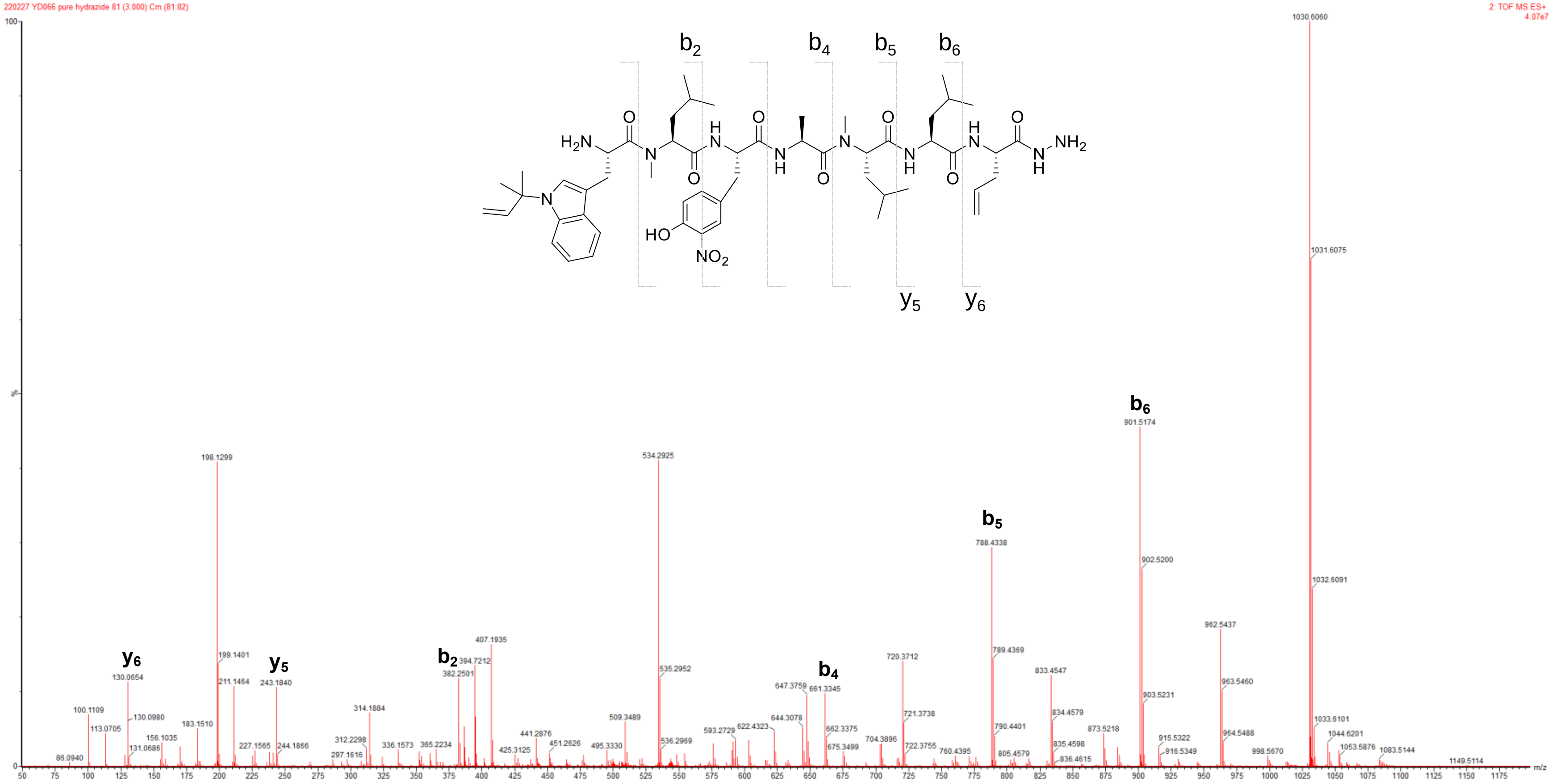

*HRMS spectra for peptide **2b**: simulated spectrum (top), measured spectrum (below).*

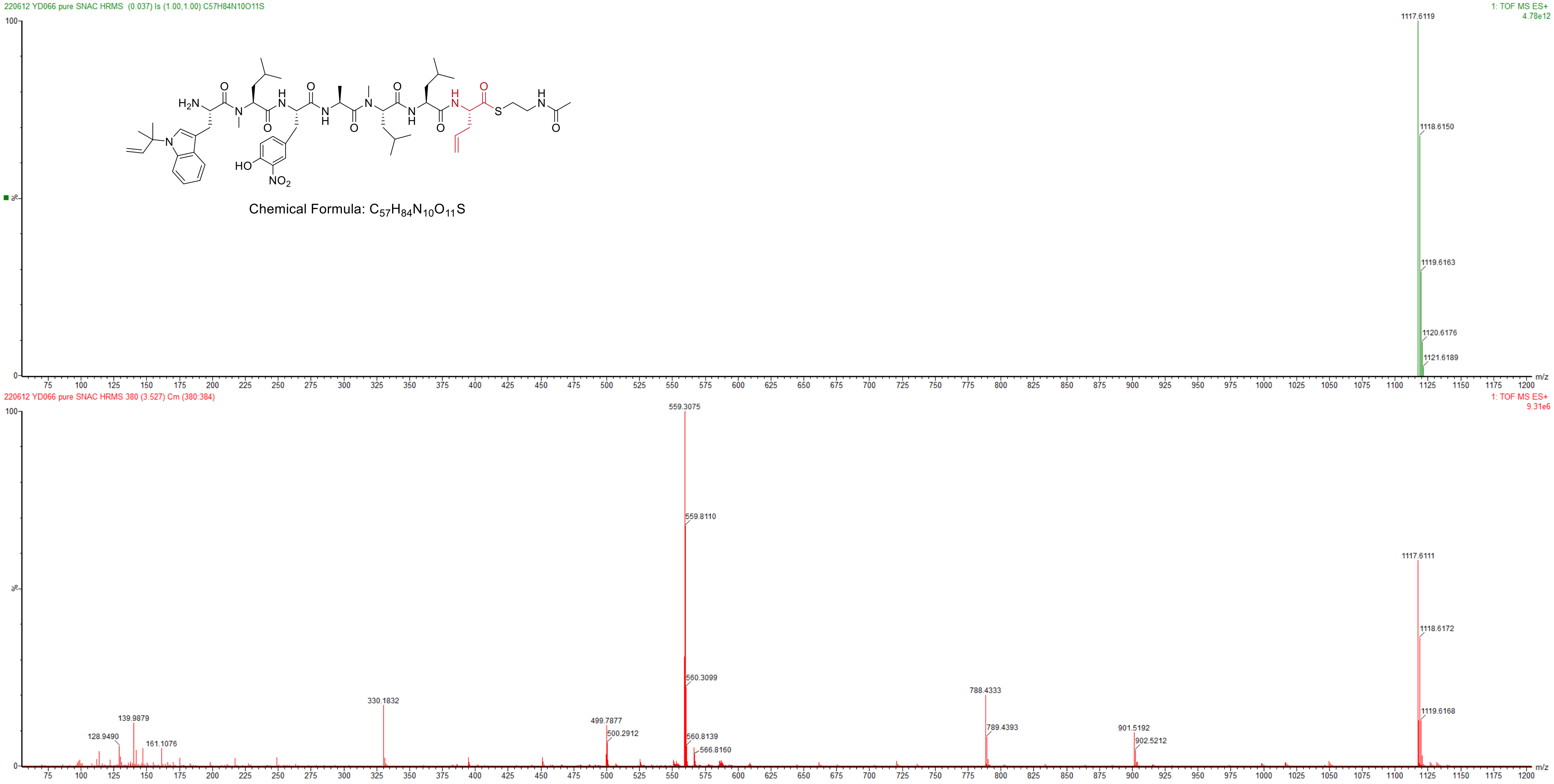

*MS^E spectrum for peptide **2b***

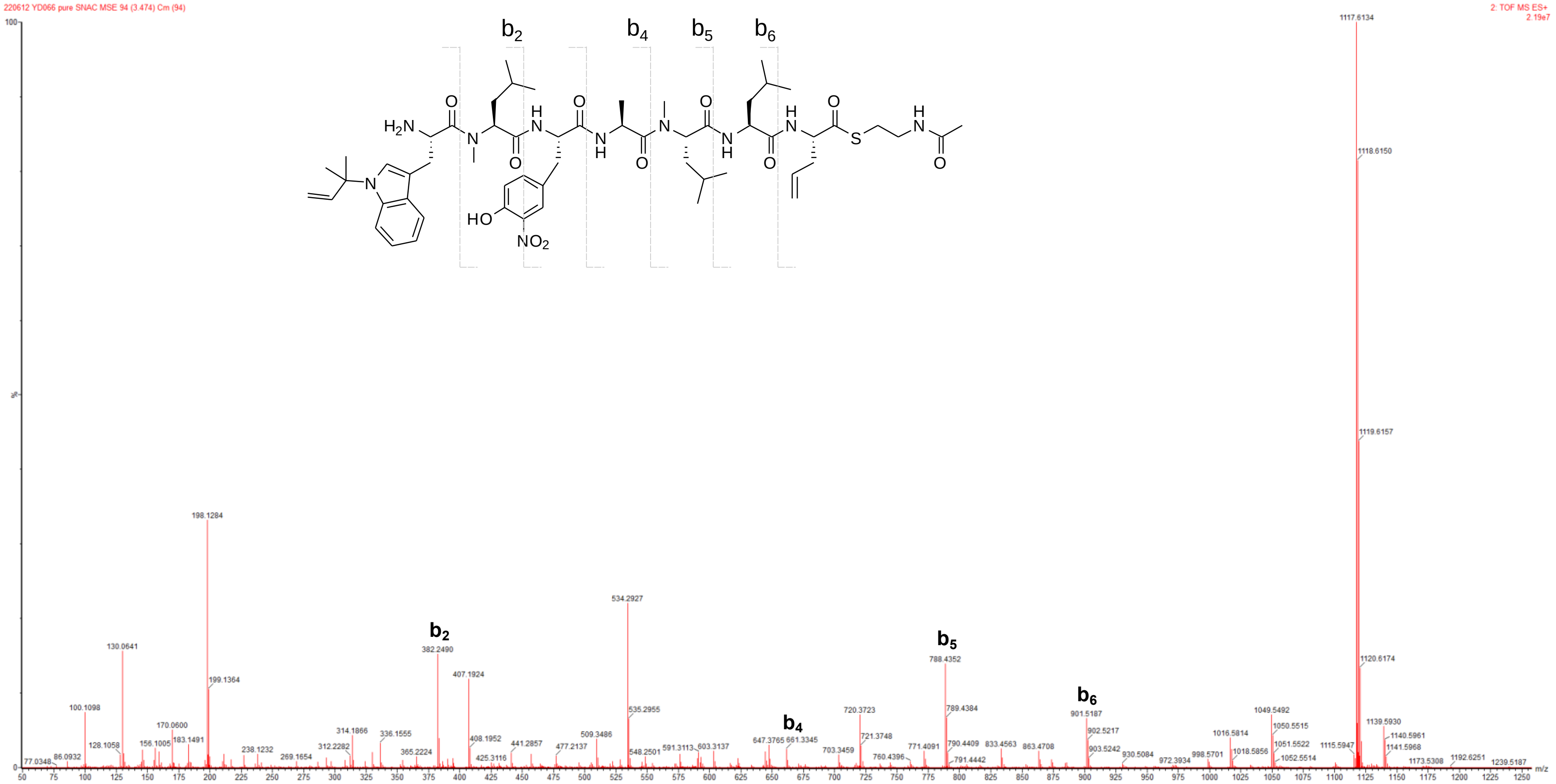

*HRMS spectra for peptide **3a$_L$**: simulated spectrum (top), measured spectrum (below).*

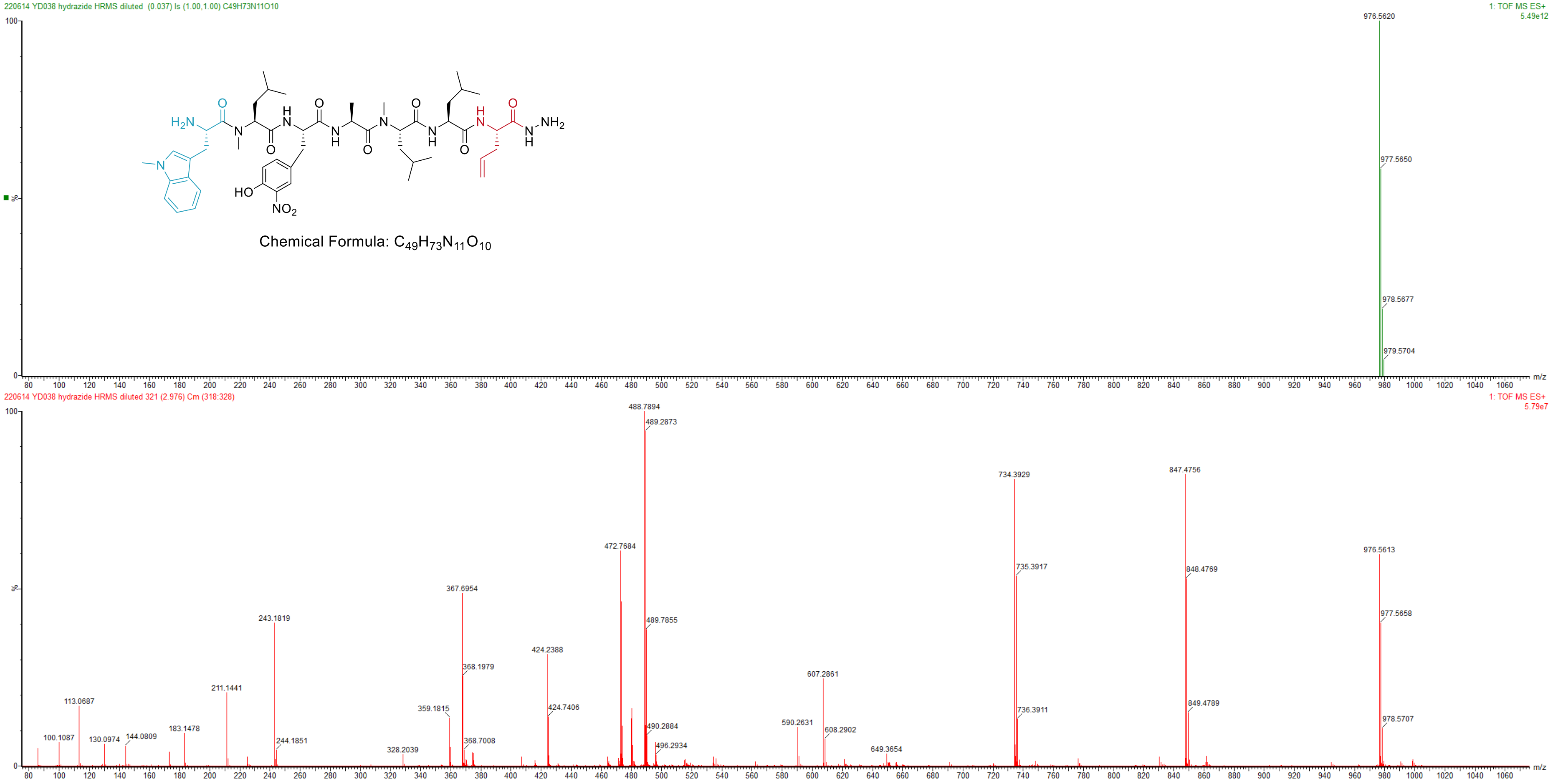

*MS^E spectrum for peptide **$3a_L$**: simulated spectrum (top), measured spectrum (below).*

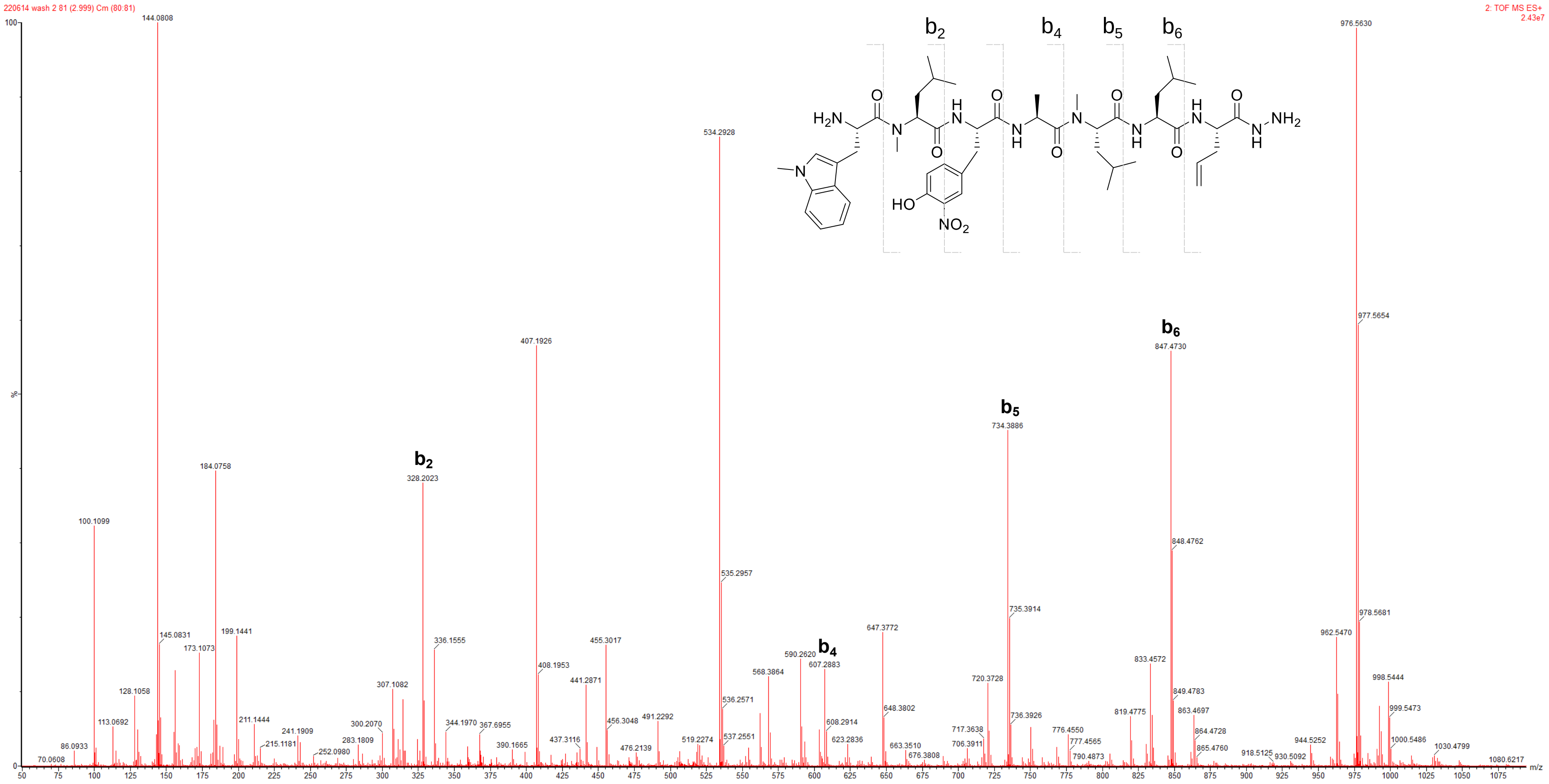

*HRMS spectra for peptide **3b**$_L$: measured spectrum (top), simulated spectrum (below),*

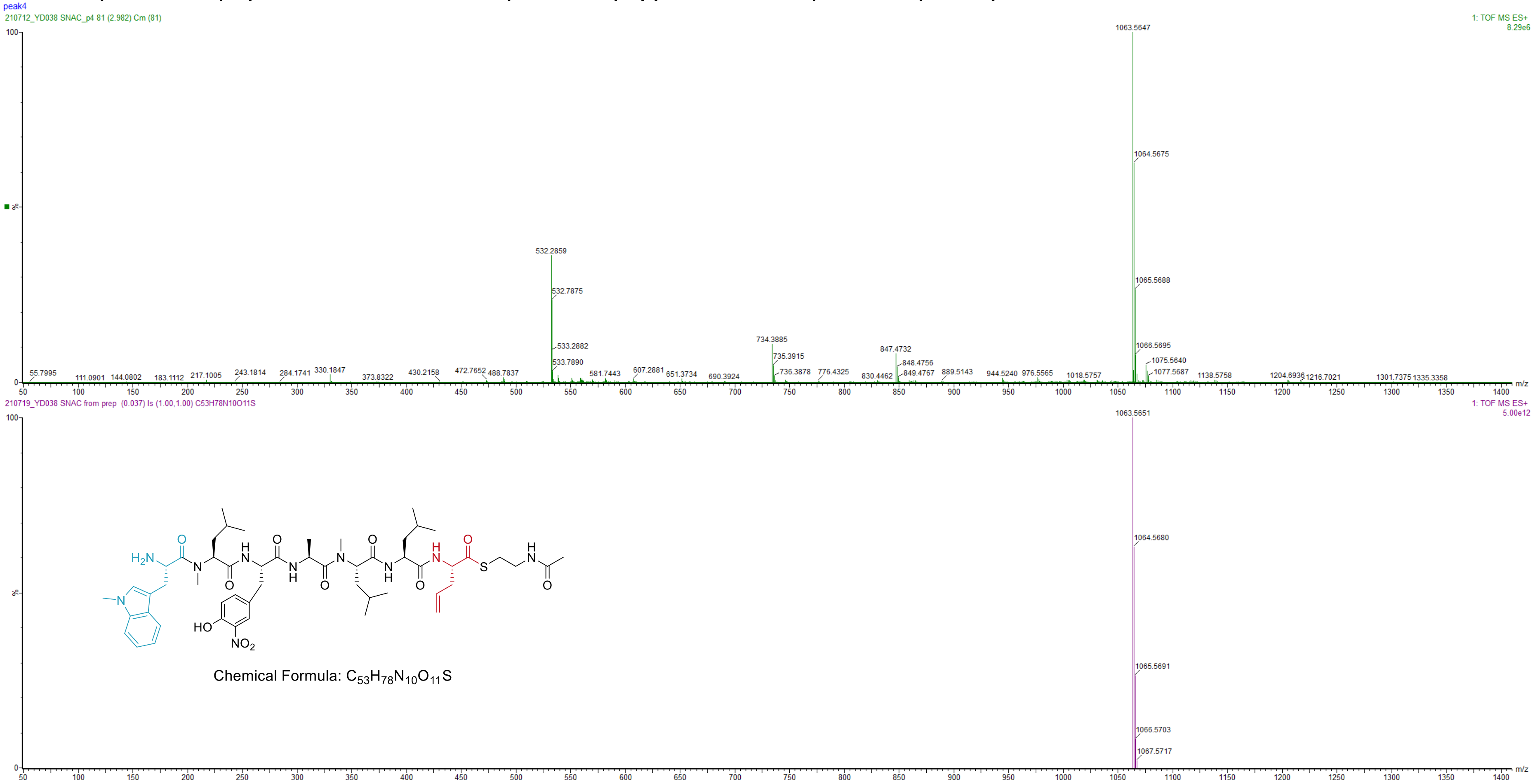

Chemical Formula: $C_{53}H_{78}N_{10}O_{11}S$

*MS^E spectrum for peptide **3b_L***

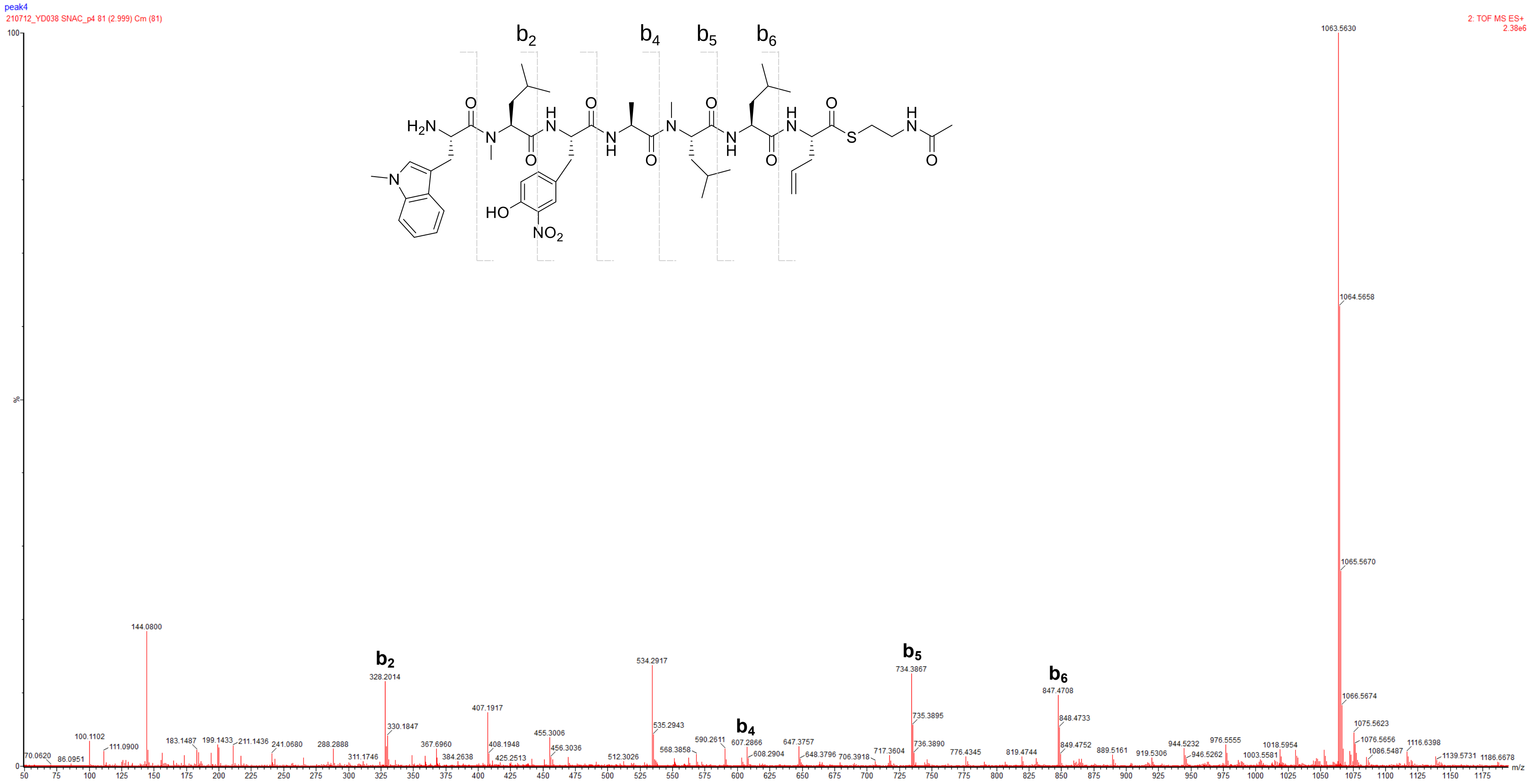

HRMS spectra for peptide ***3a$_D$****: simulated spectrum (top), measured spectrum (below).*

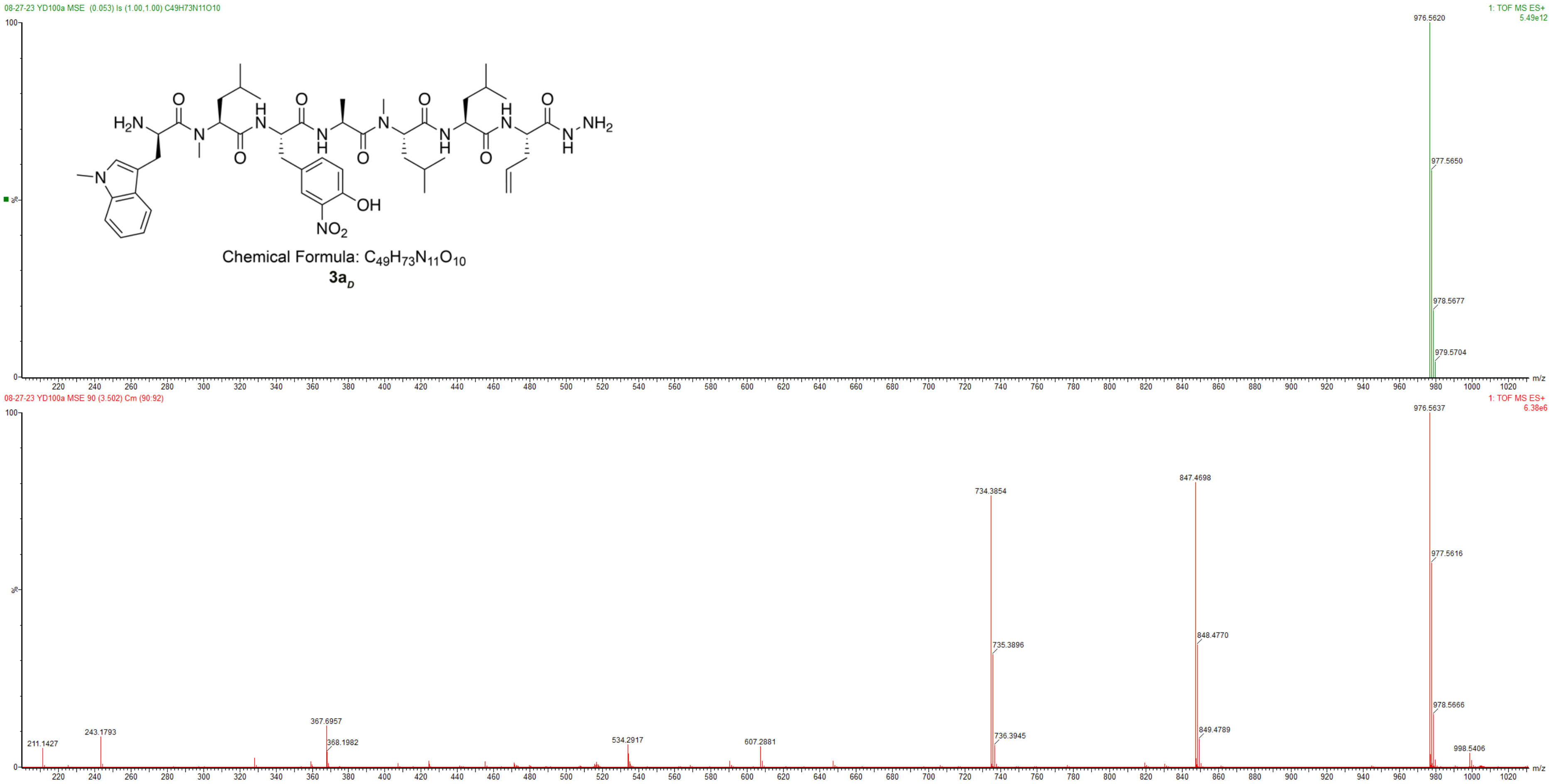

MS^E spectrum for peptide ***3a_D***

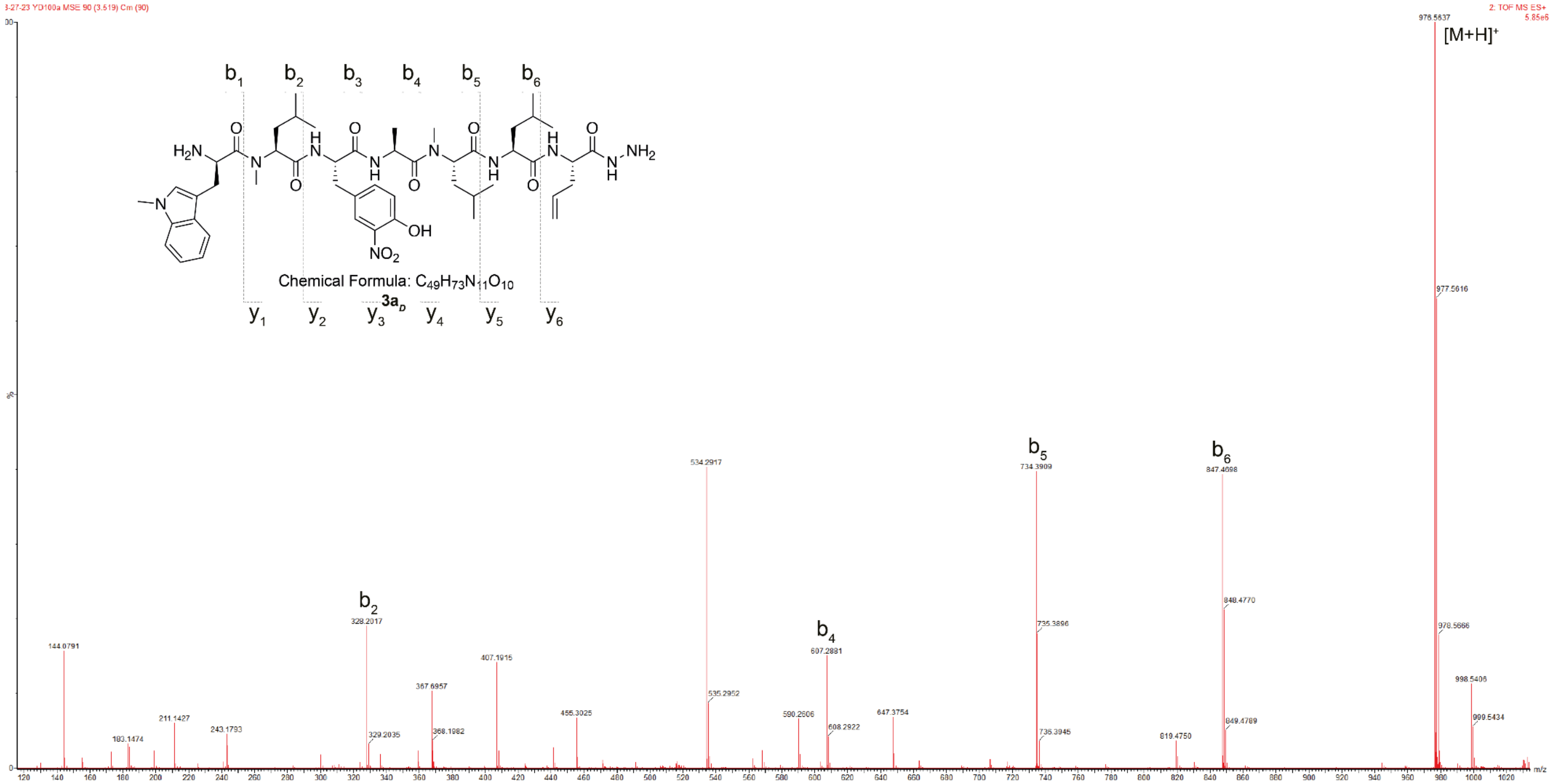

*HRMS spectra for peptide **3b$_D$**: simulated spectrum (top), measured spectrum (below).*

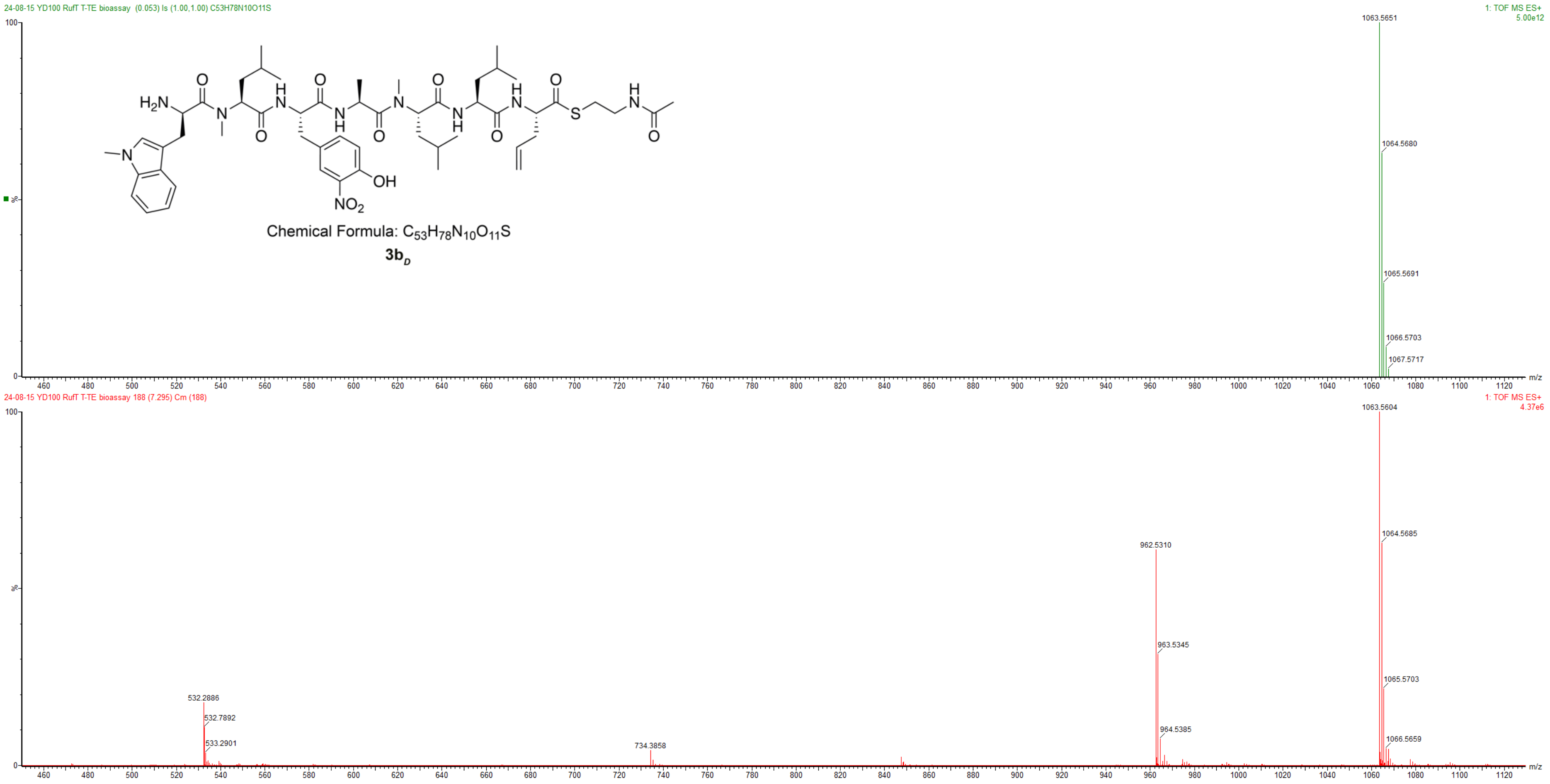

*MS^E spectrum for peptide **3b$_D$***

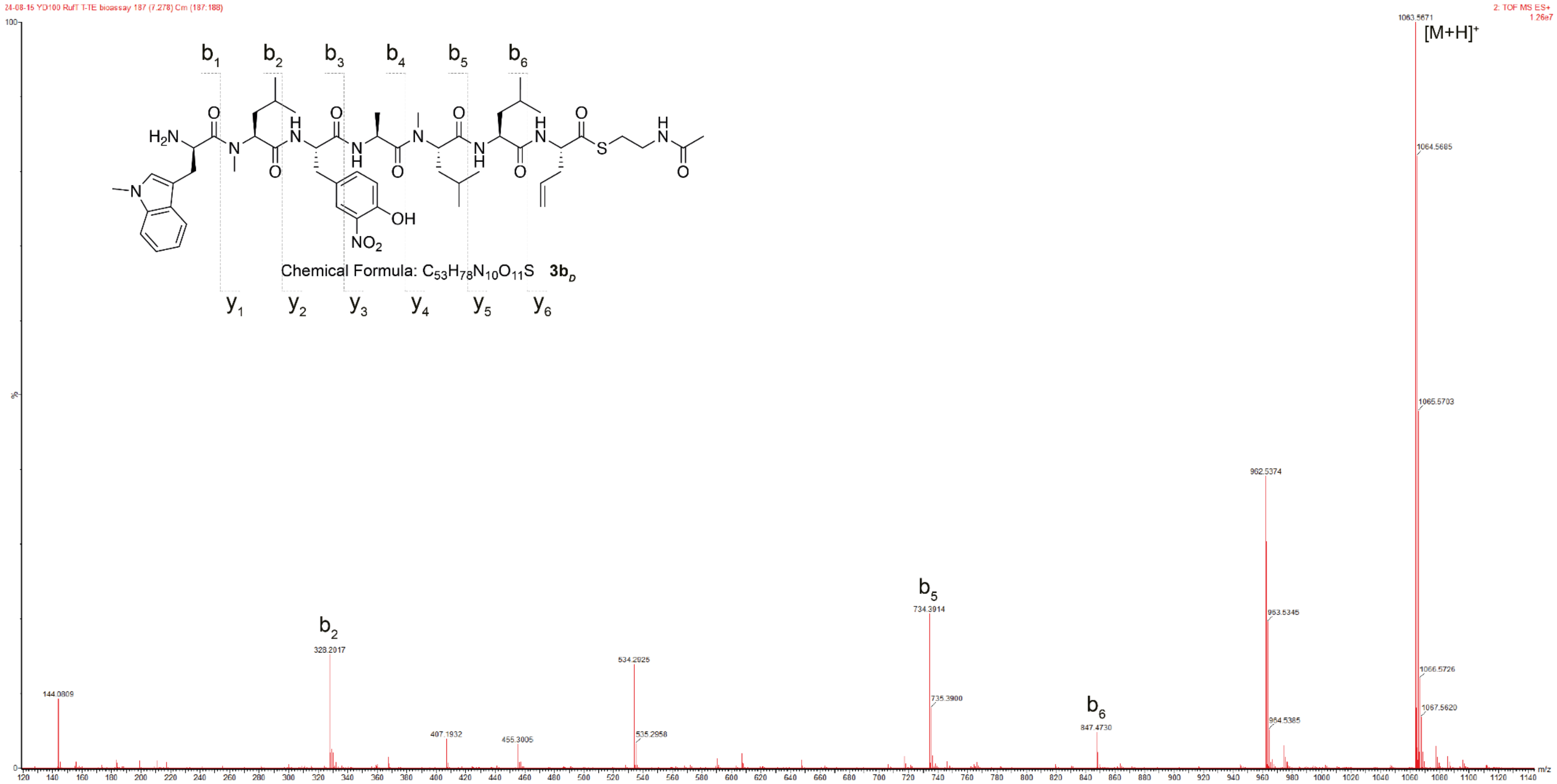

*HRMS spectra for peptide **4a**: simulated spectrum (top), measured spectrum (below).*

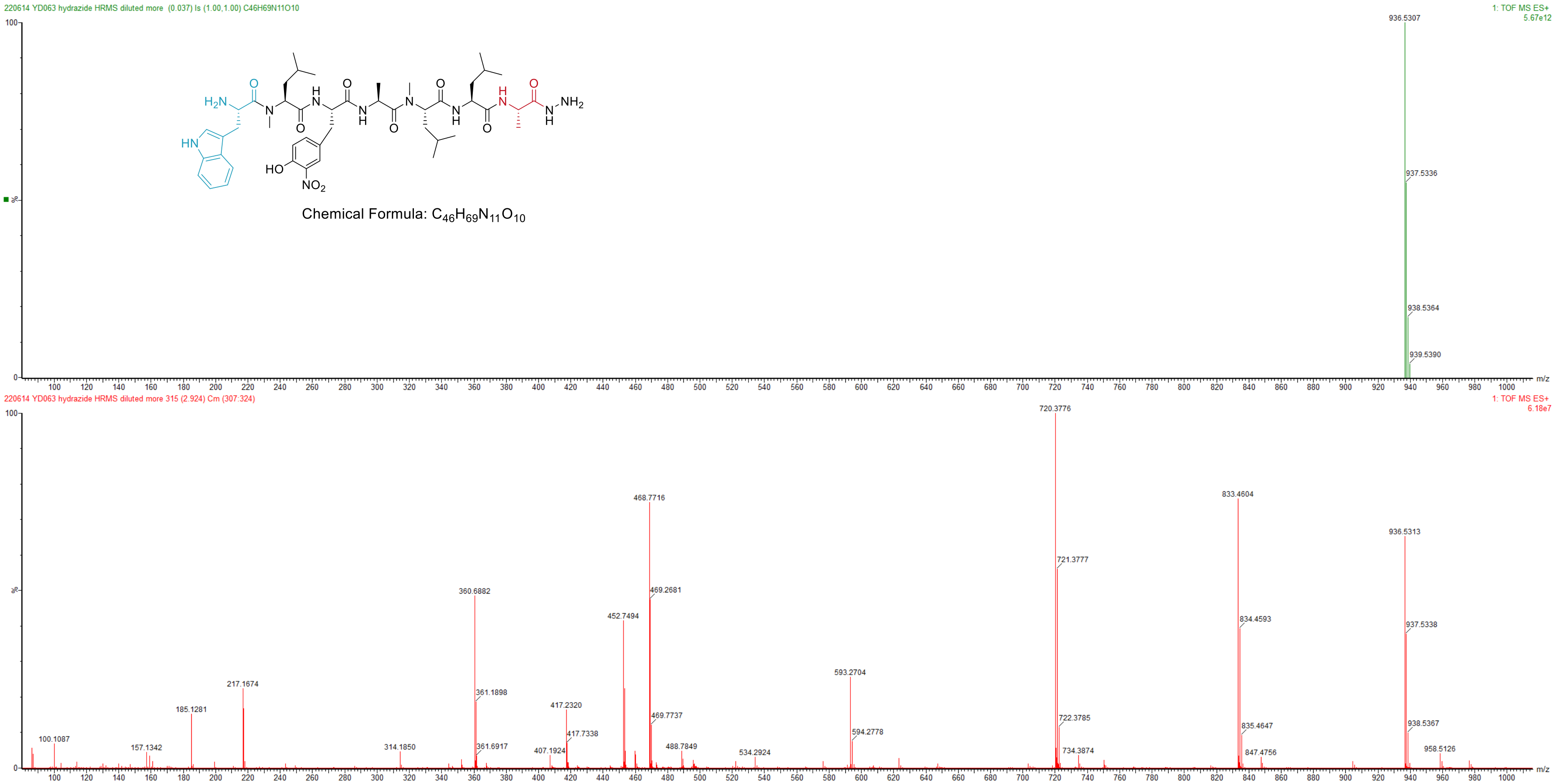

*MS^E spectrum for peptide **4a***

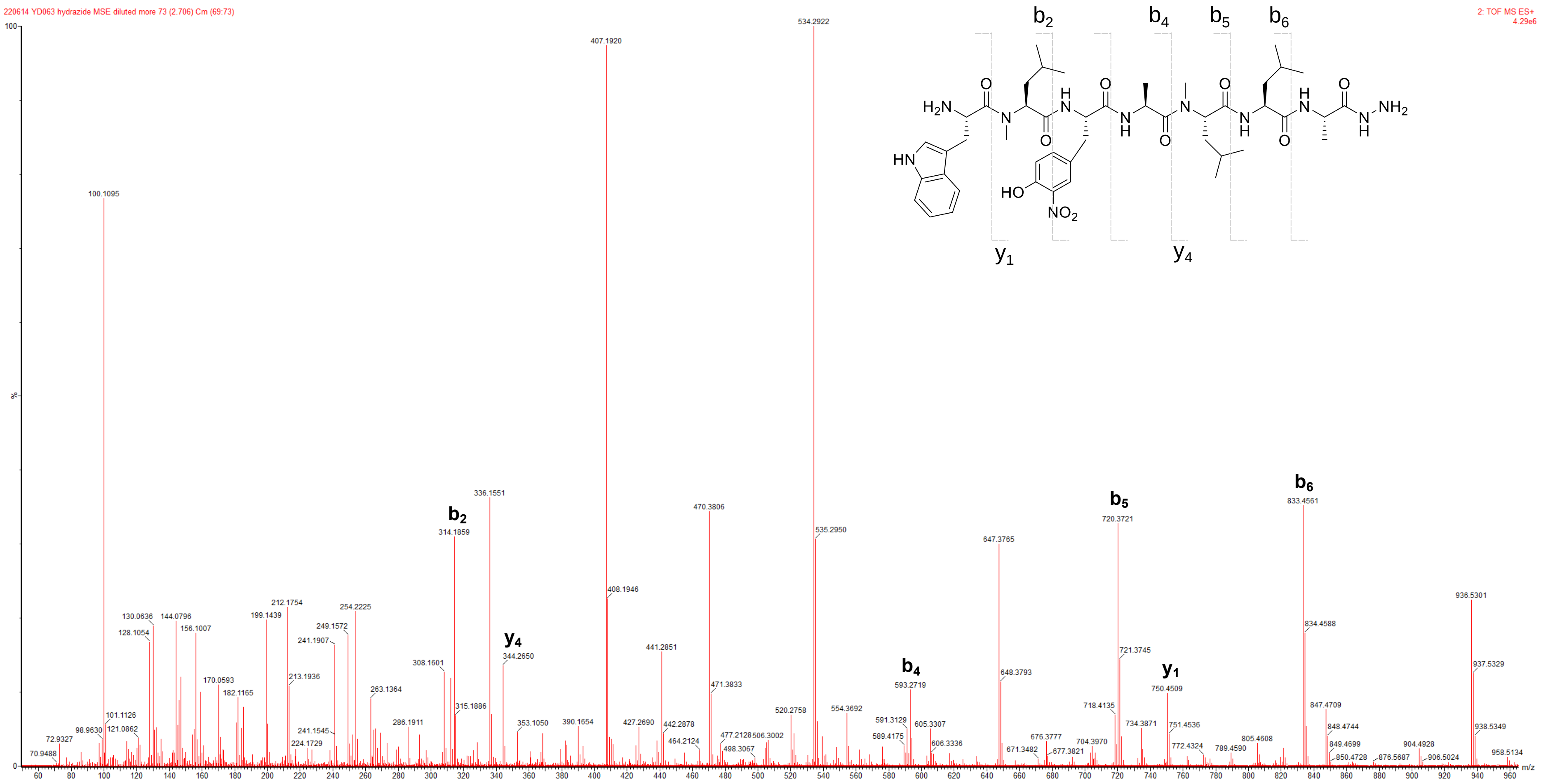

*HRMS spectrum for peptide **4b**: simulated spectrum (top), measured spectrum (below).*

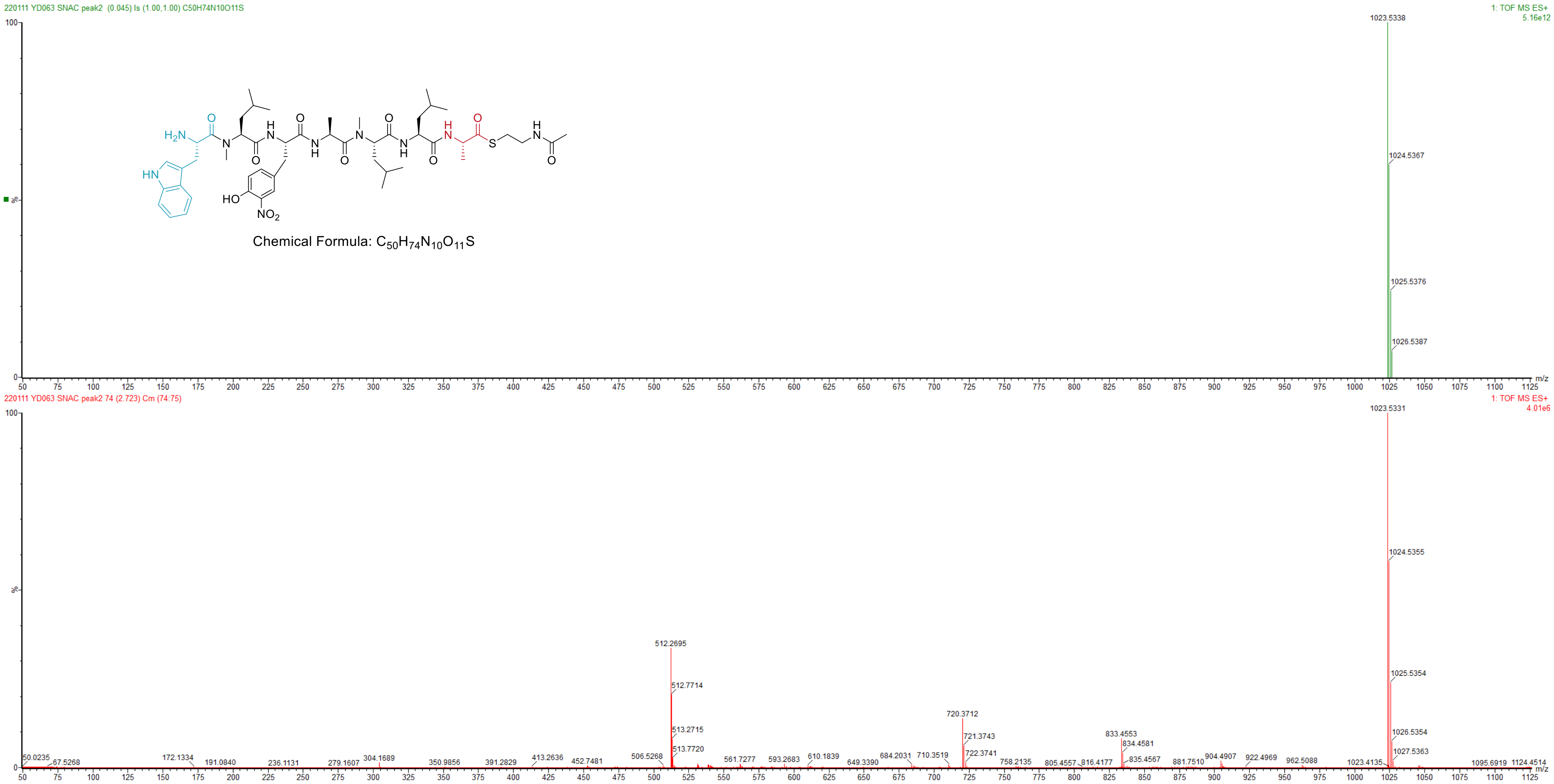

*MS^E spectrum for peptide **4b***

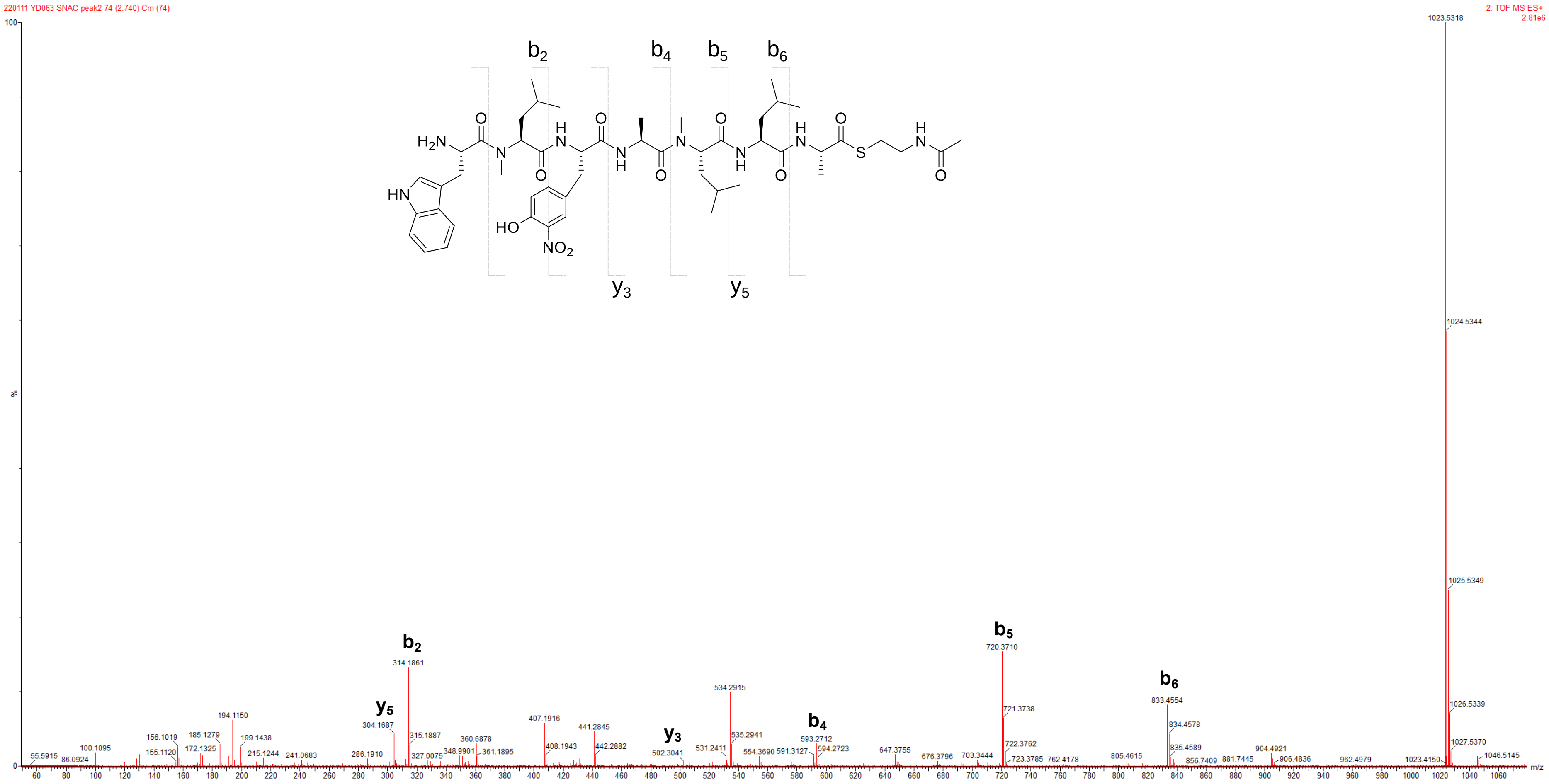

*HRMS spectra for peptide **5a**: simulated spectrum (top), measured spectrum (below).*

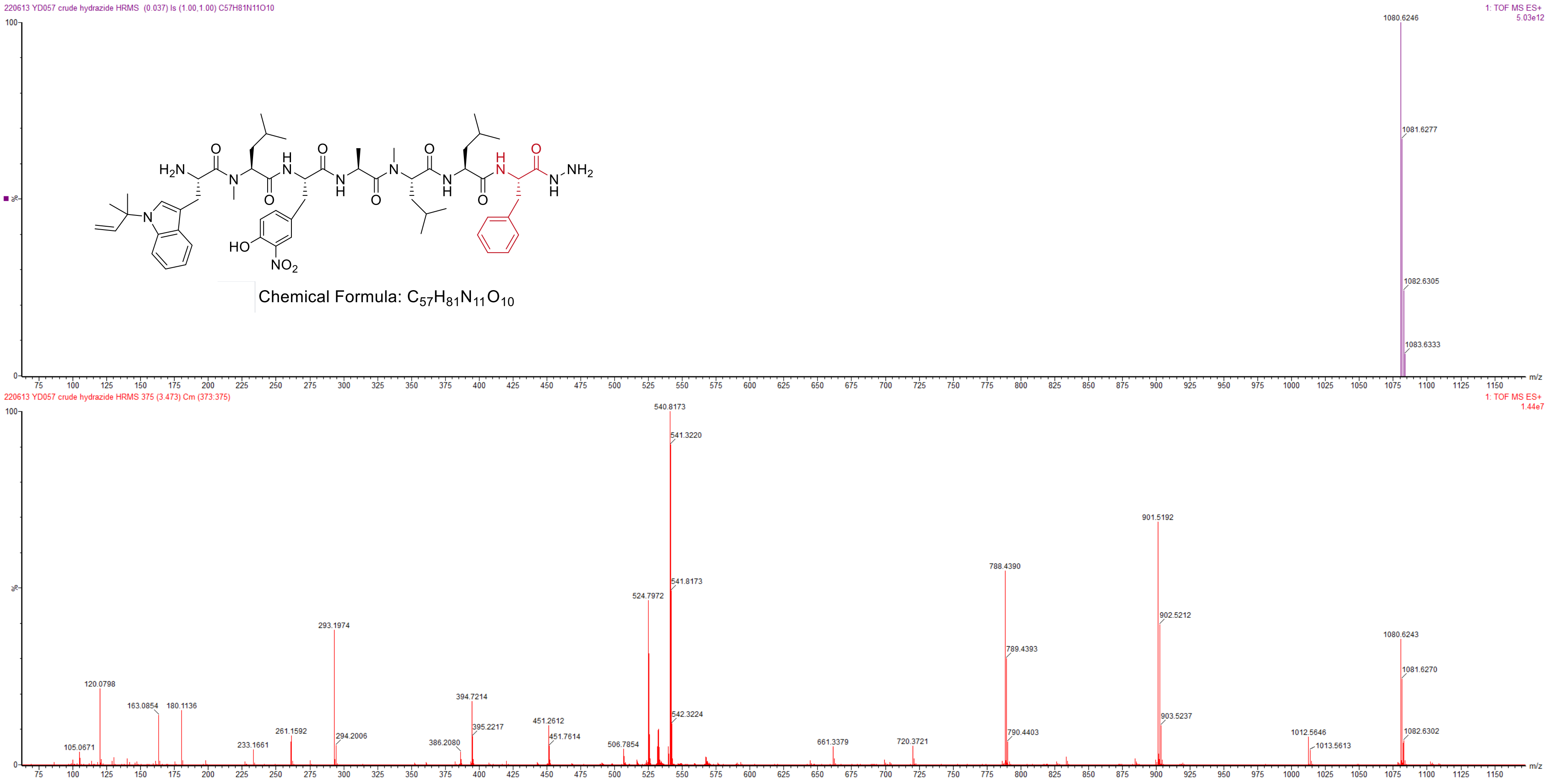

*MS^E spectrum for peptide **5a***

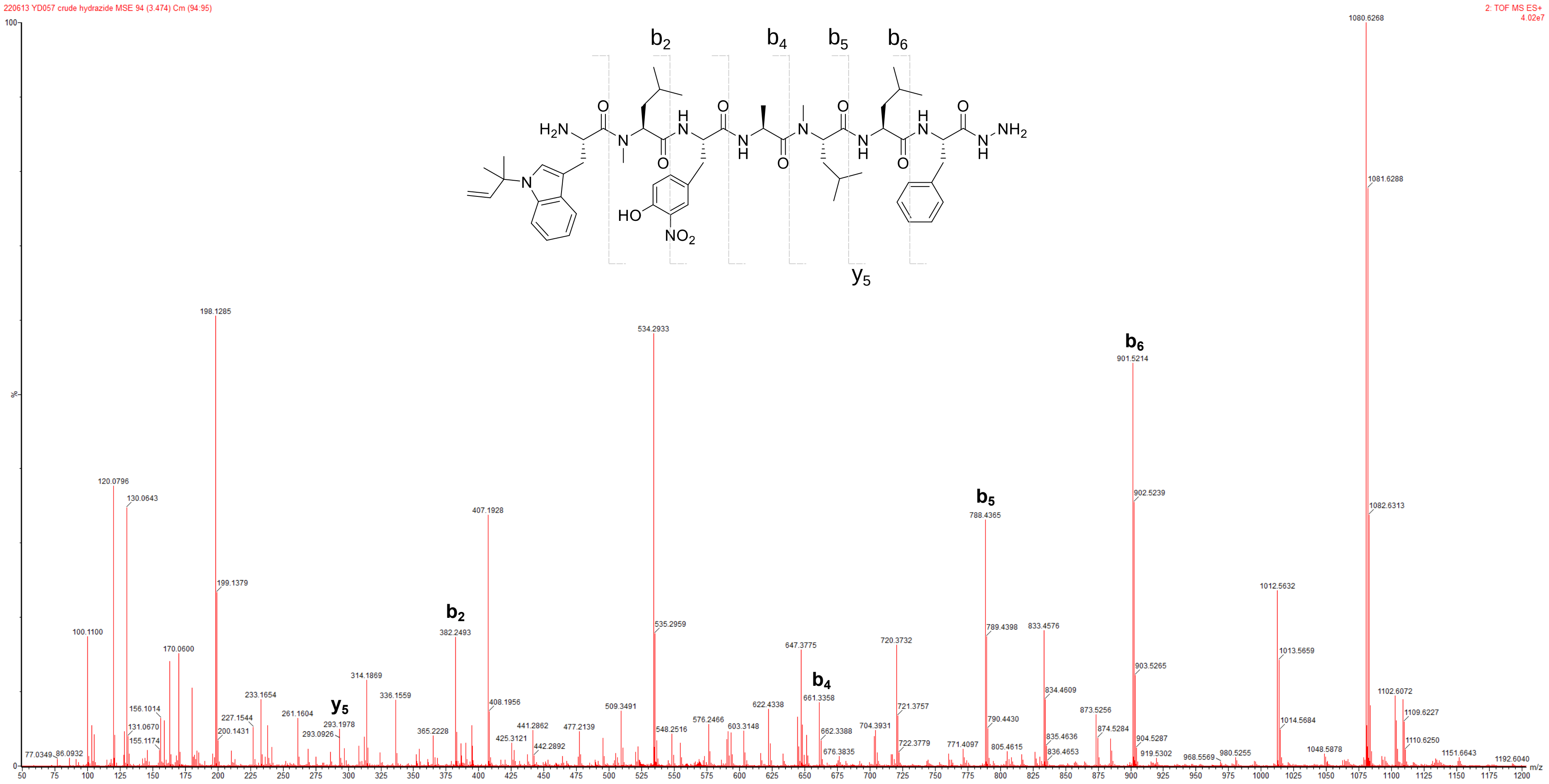

*HRMS spectra for peptide **5b**: simulated spectrum (top), measured spectrum (below).*

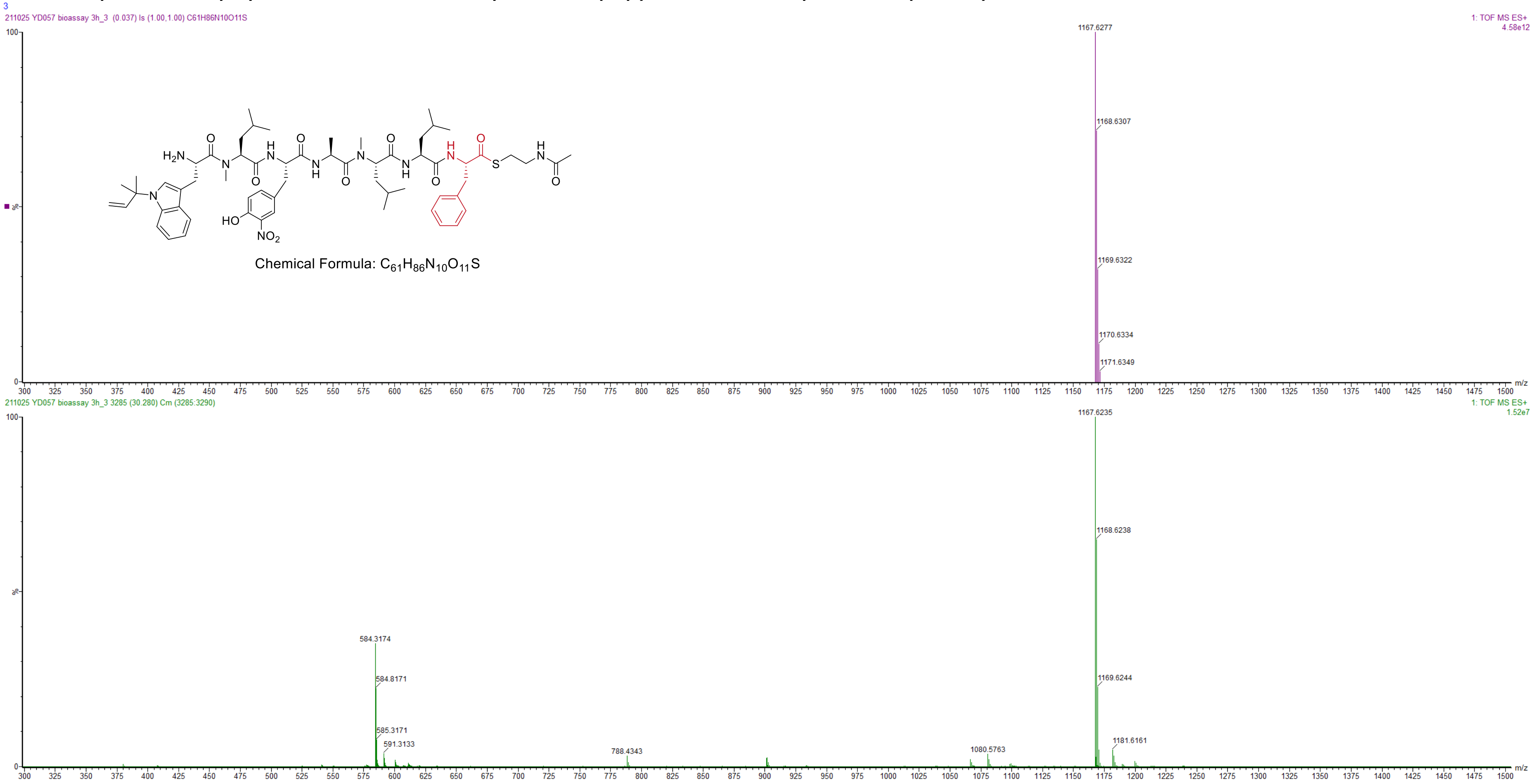

MS^E spectrum for peptide ***5b***

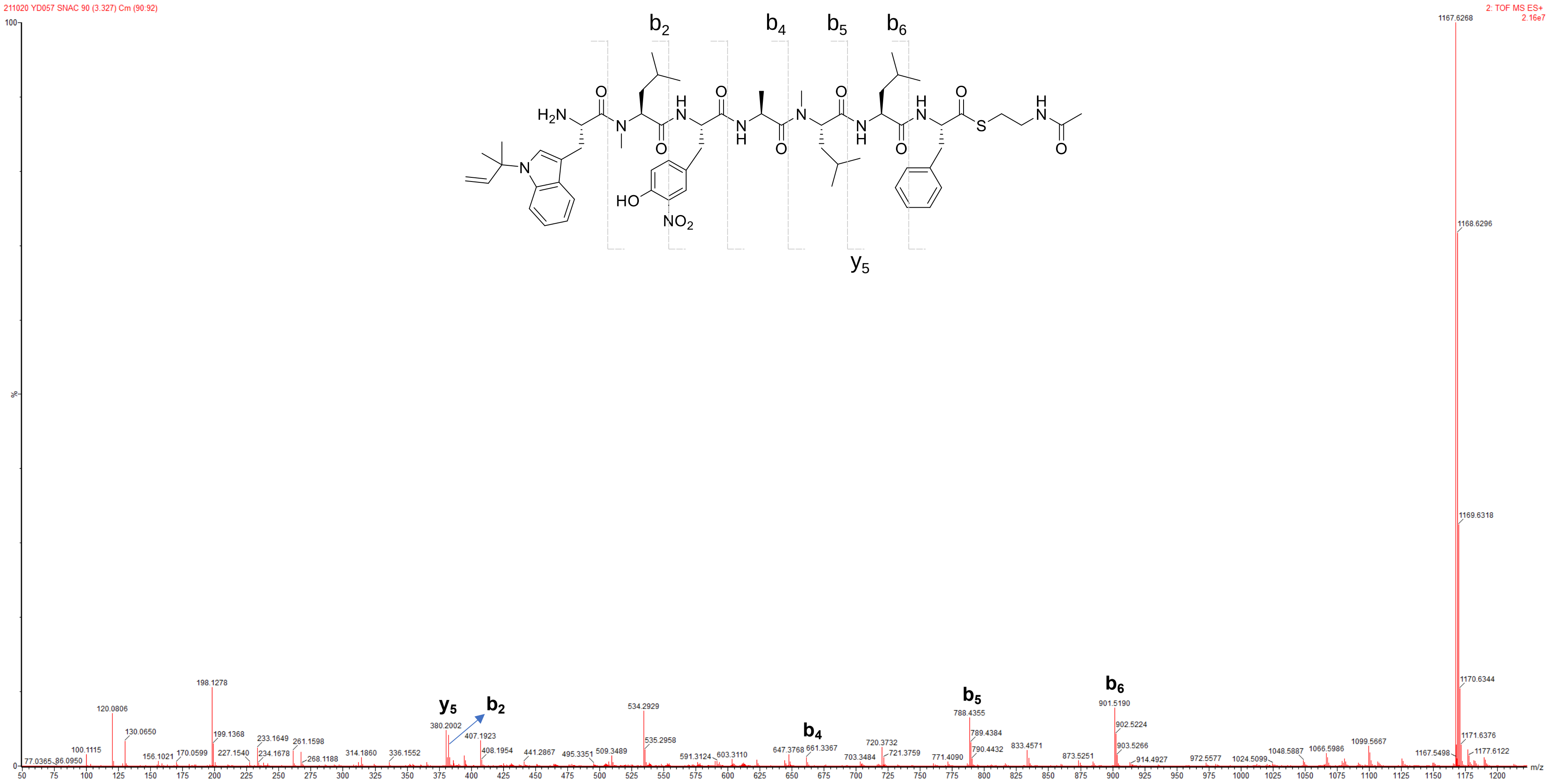

*HRMS spectra for peptide **6a**: simulated spectrum (top), measured spectrum (below).*

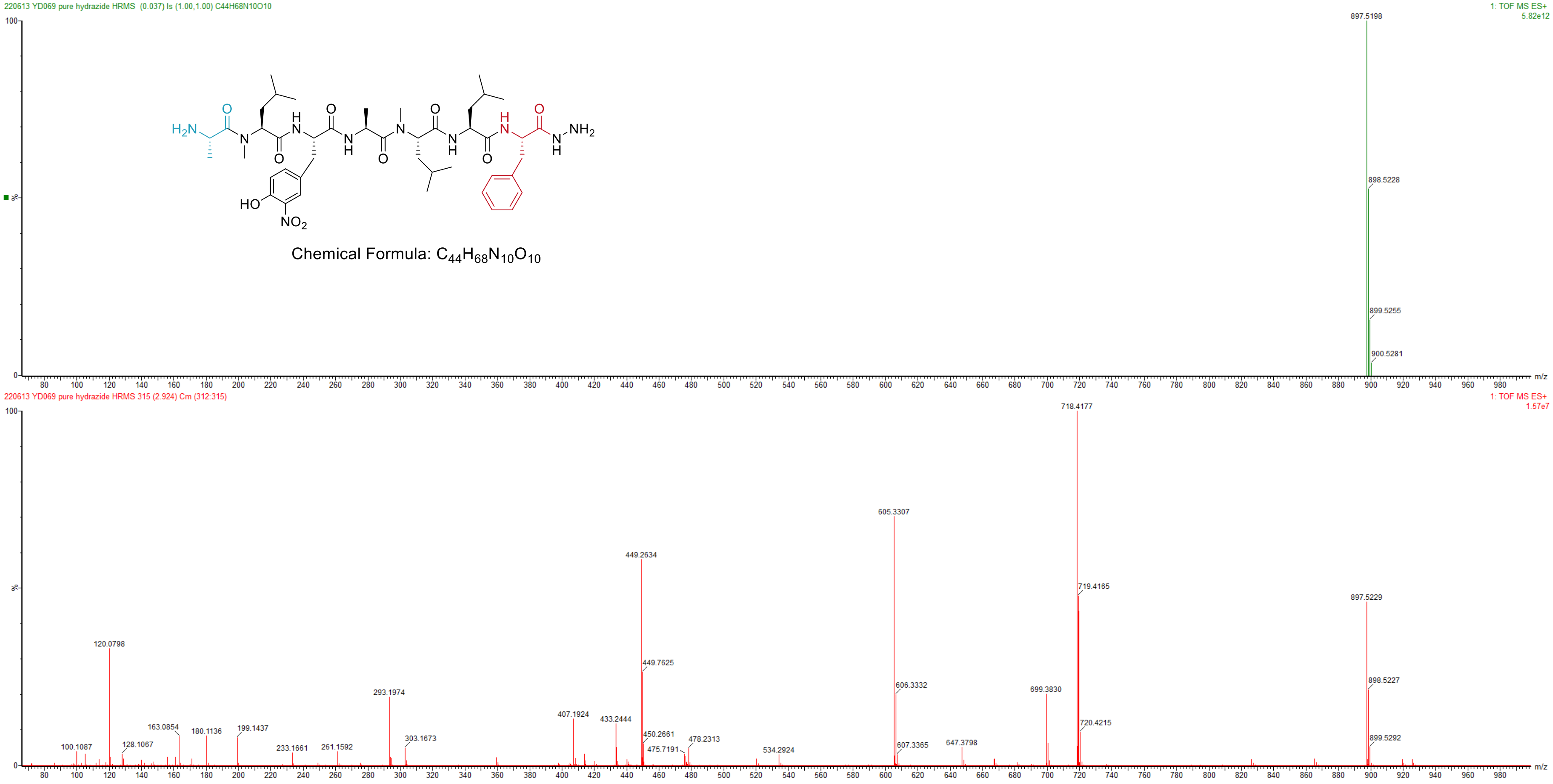

*MS^E spectrum for peptide **6a***

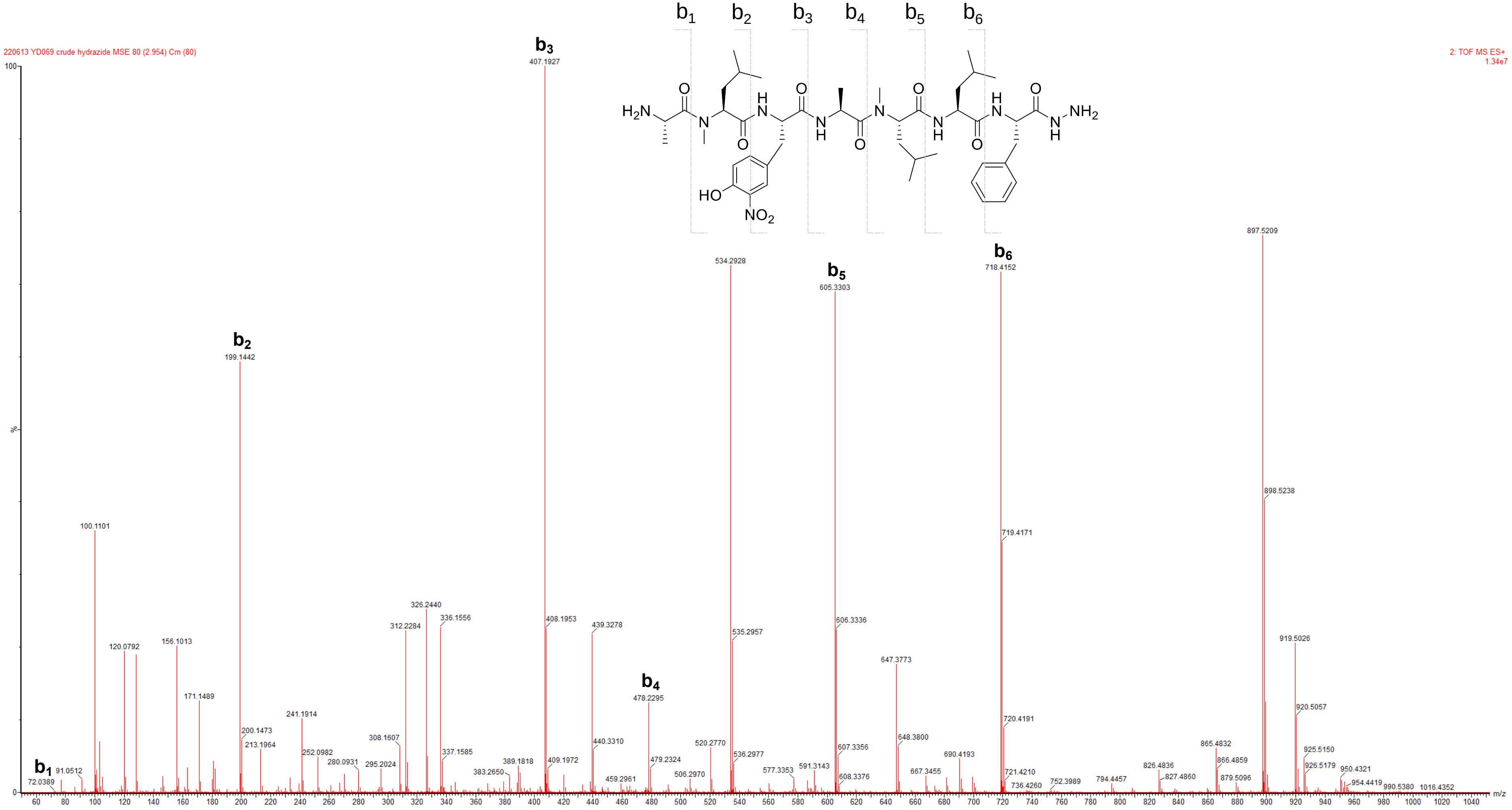

HRMS spectra for peptide ***6b****: simulated spectrum (top), measured spectrum (below).*

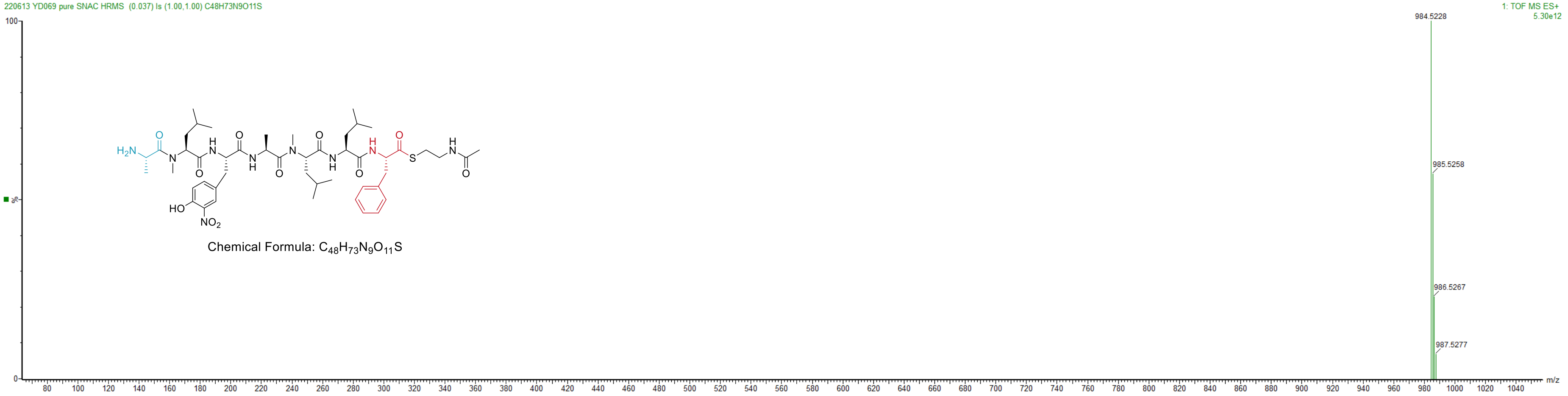

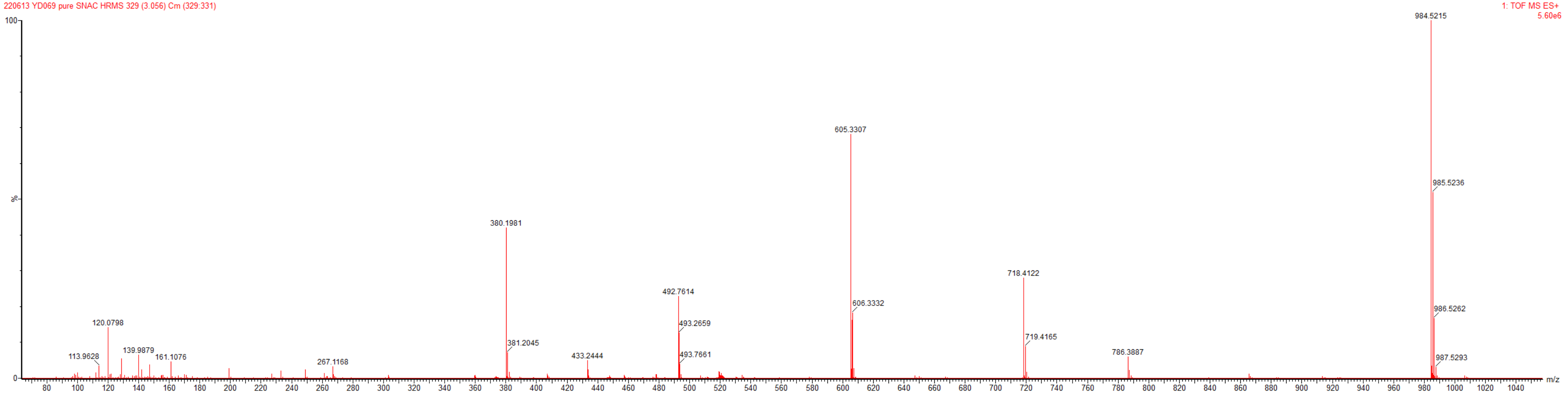

*MS^E spectrum for peptide **6b***

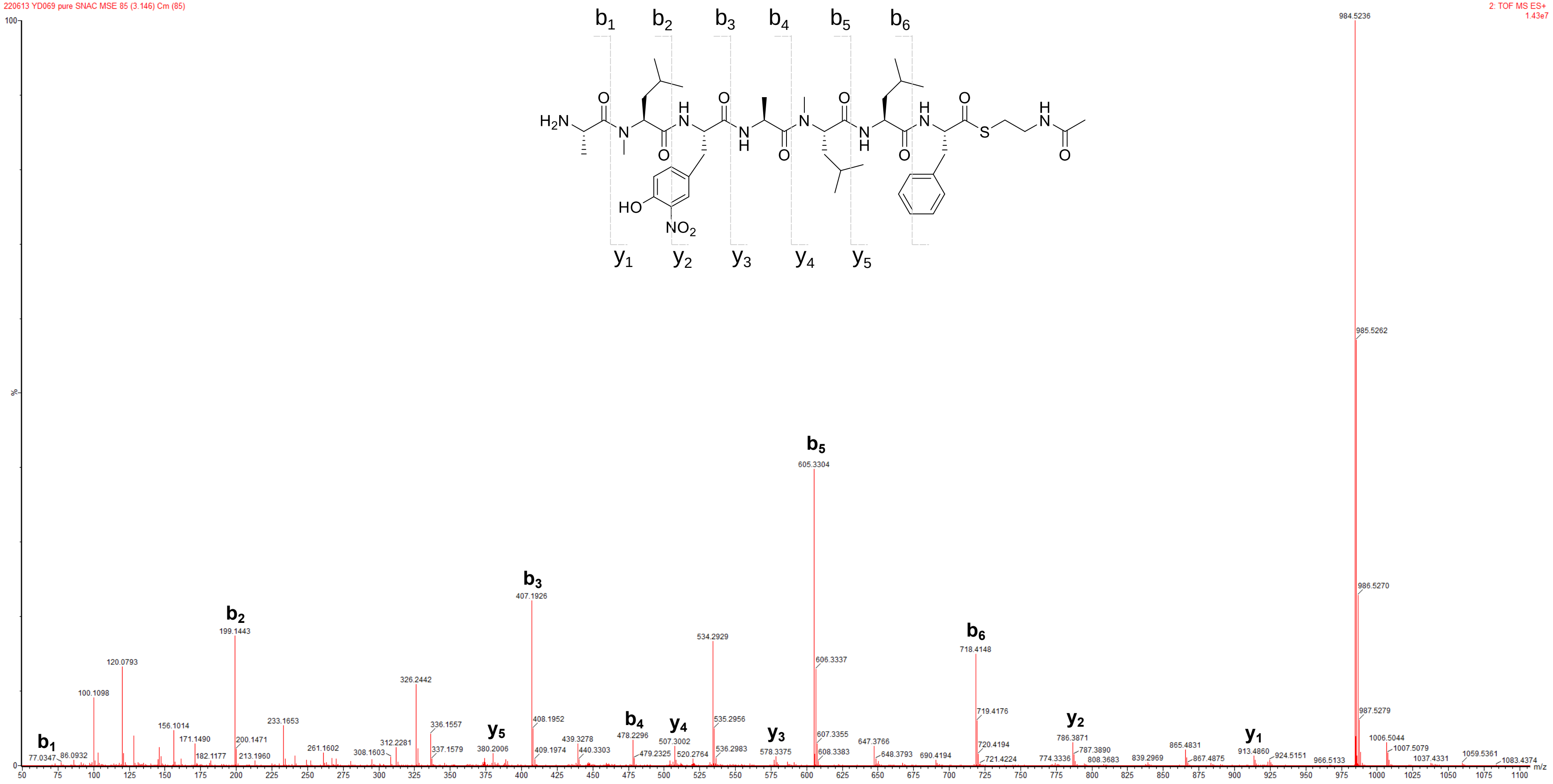

*HRMS spectra for peptide **7a**: measured spectrum (top), simulated spectrum (below),*

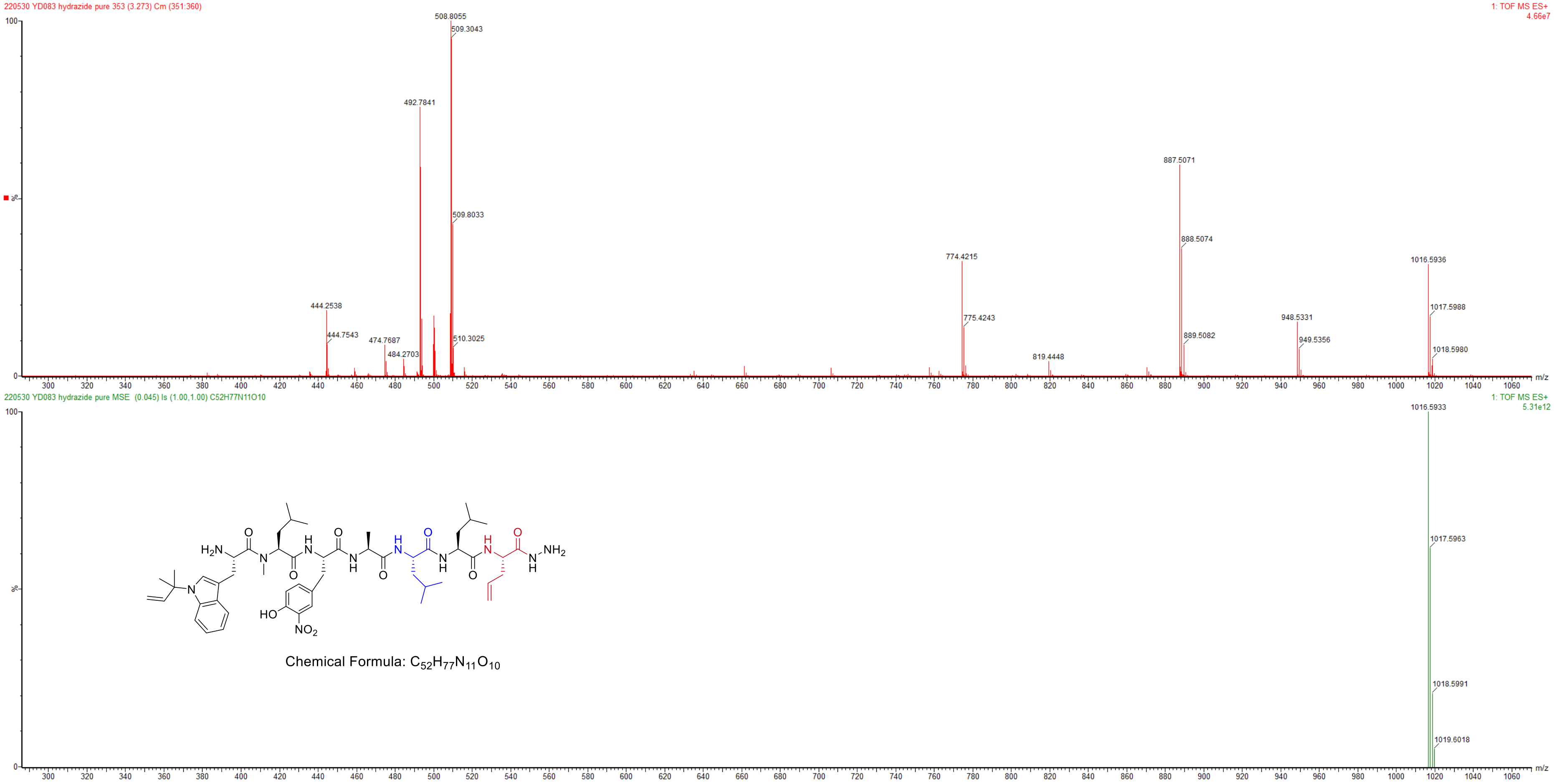

*MS^E spectrum for peptide **7a***

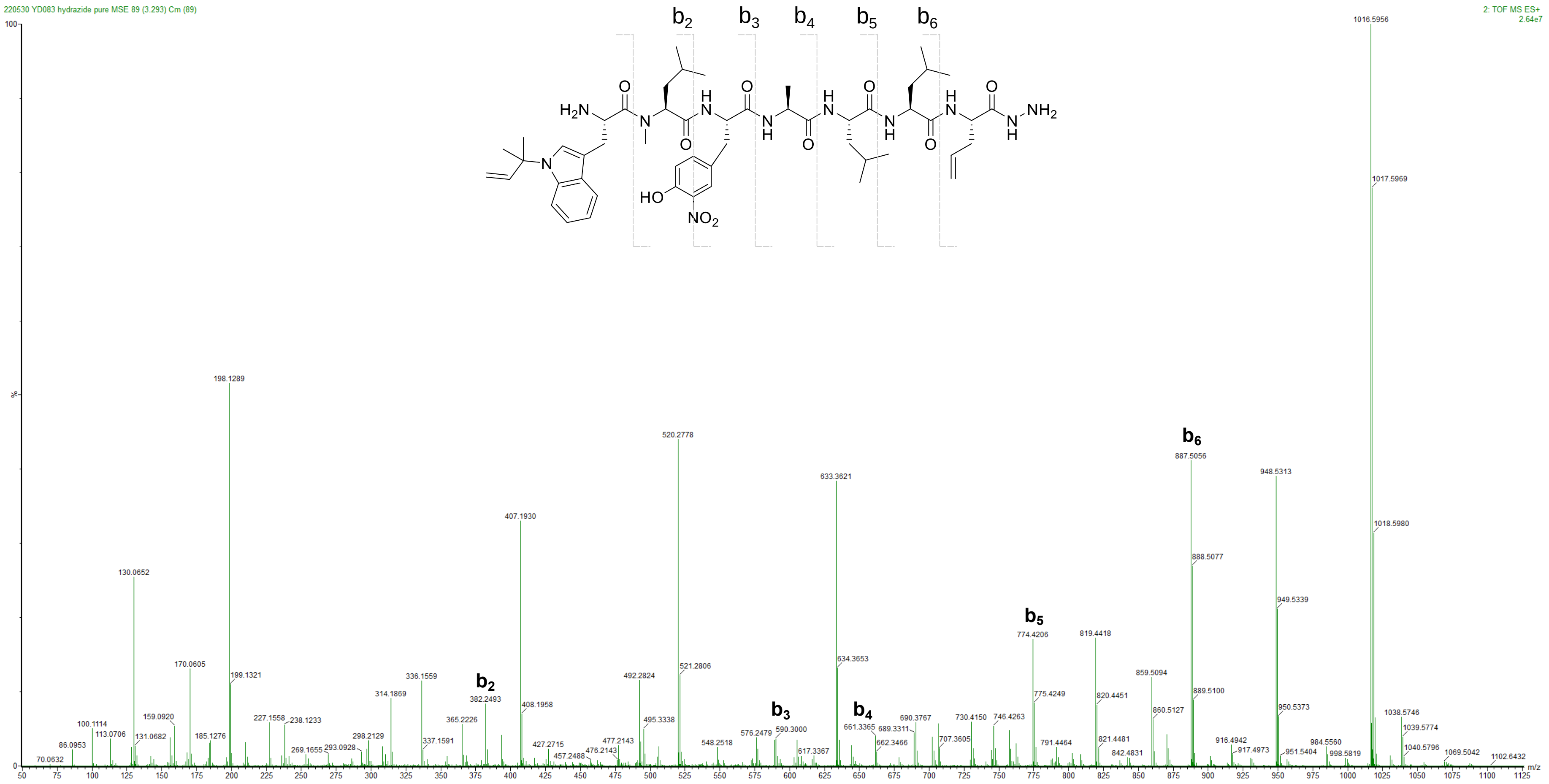

*HRMS spectrum for peptide **7b**: simulated spectrum (top), measured spectrum (below).*

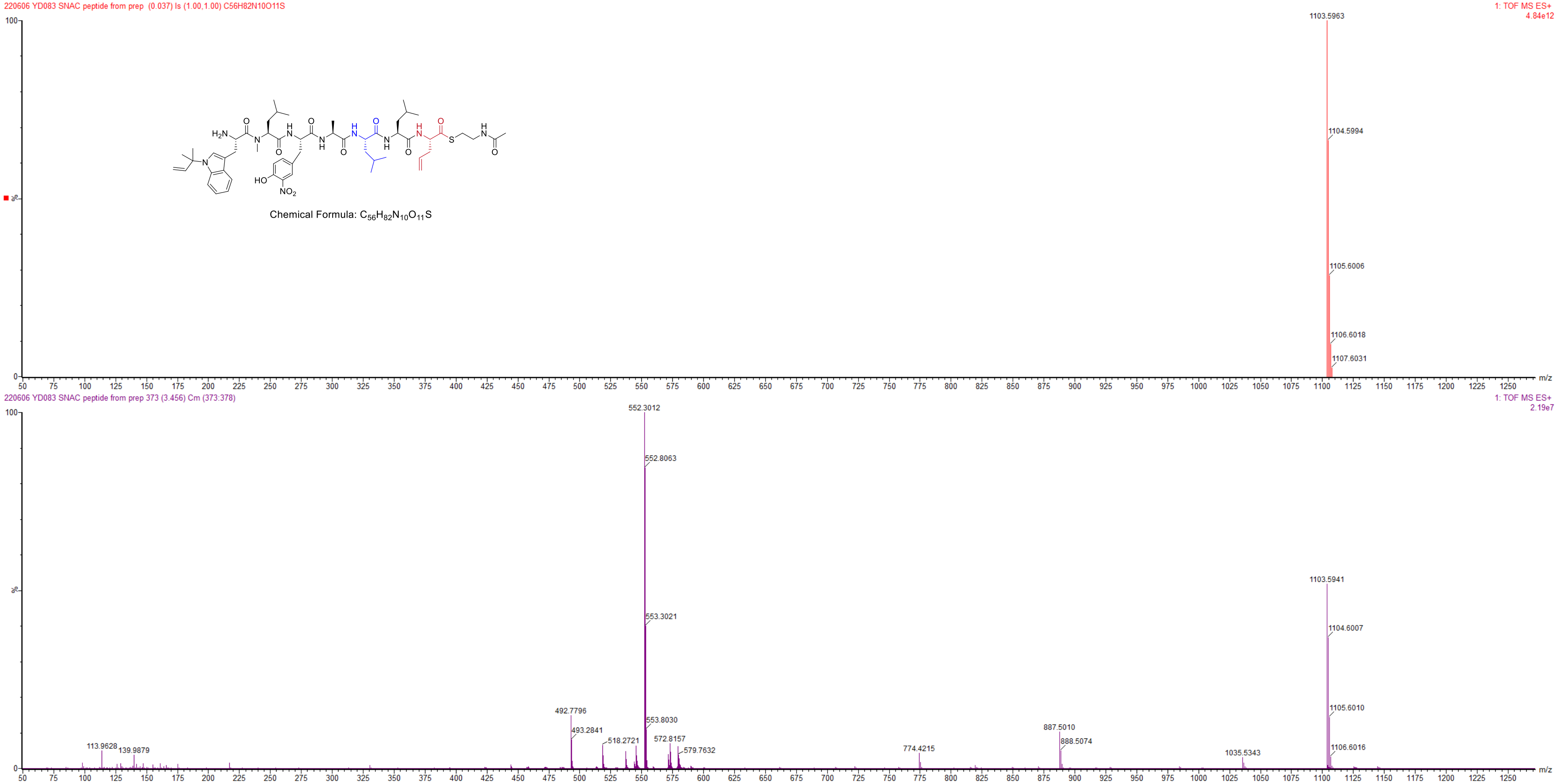

*MS^E spectra for peptide **7b***

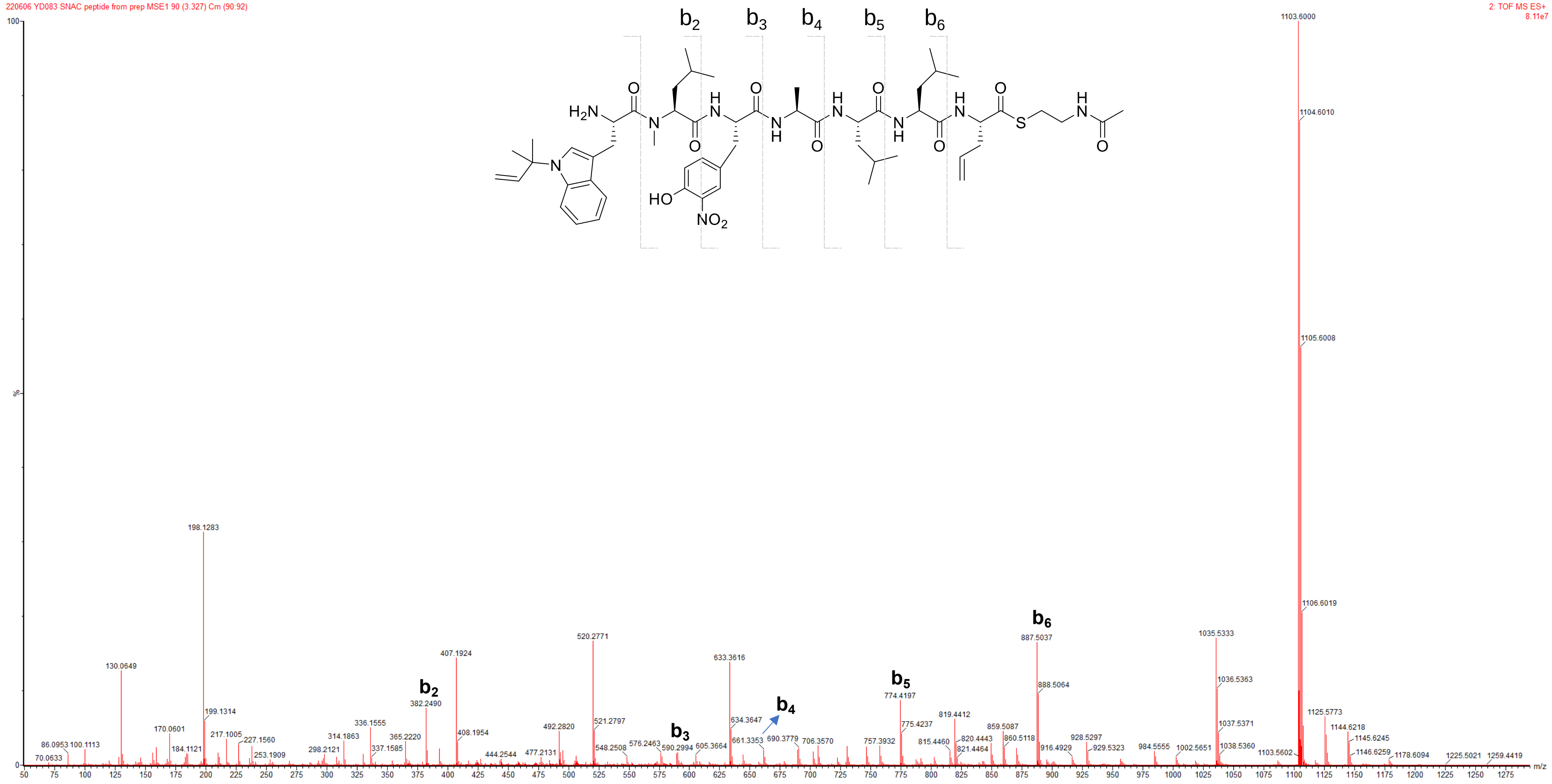

*HRMS spectrum for peptide **8a**: simulated spectrum (top), measured spectrum (below).*

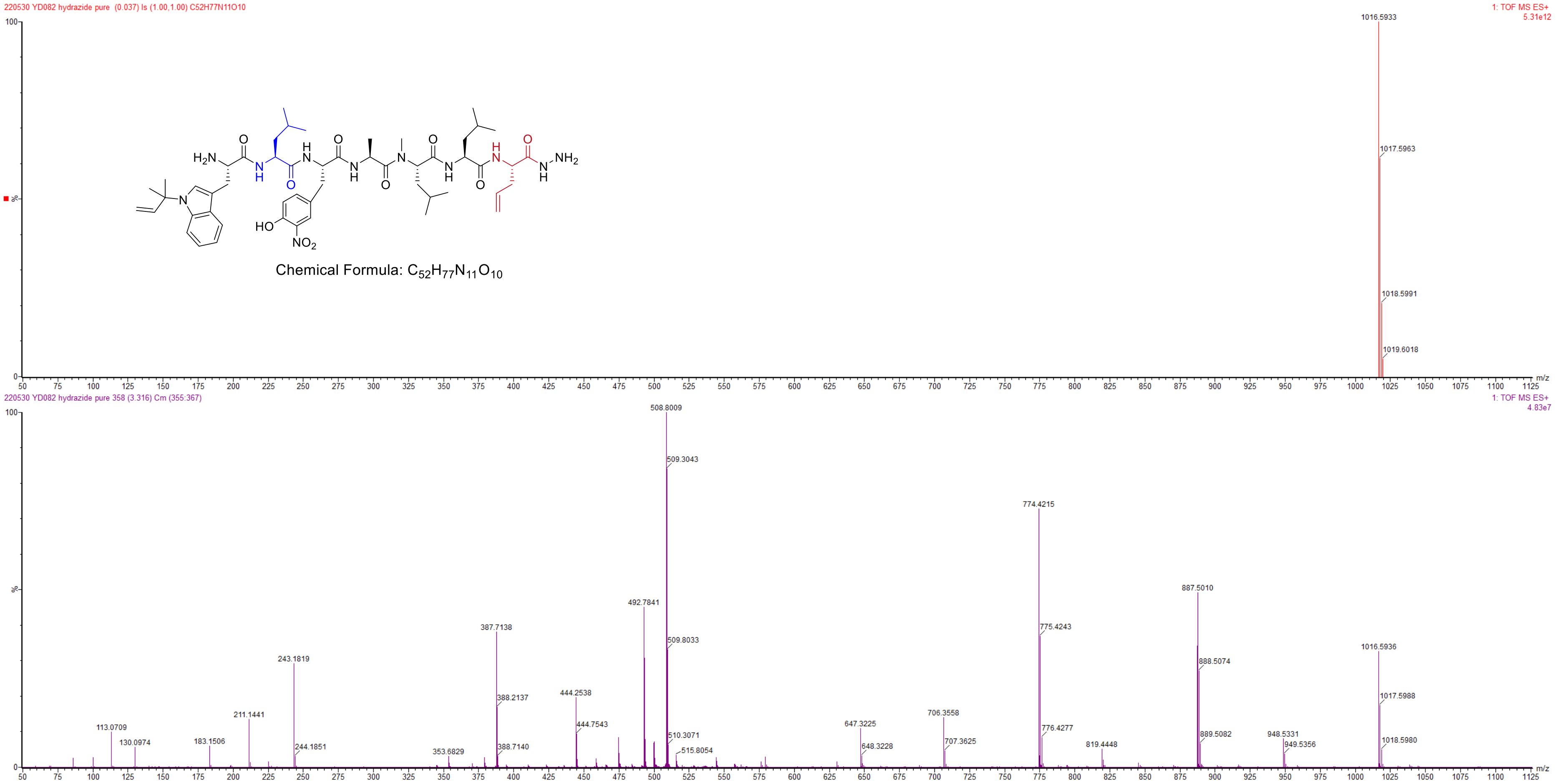

*MS^E spectrum for peptide **8a***

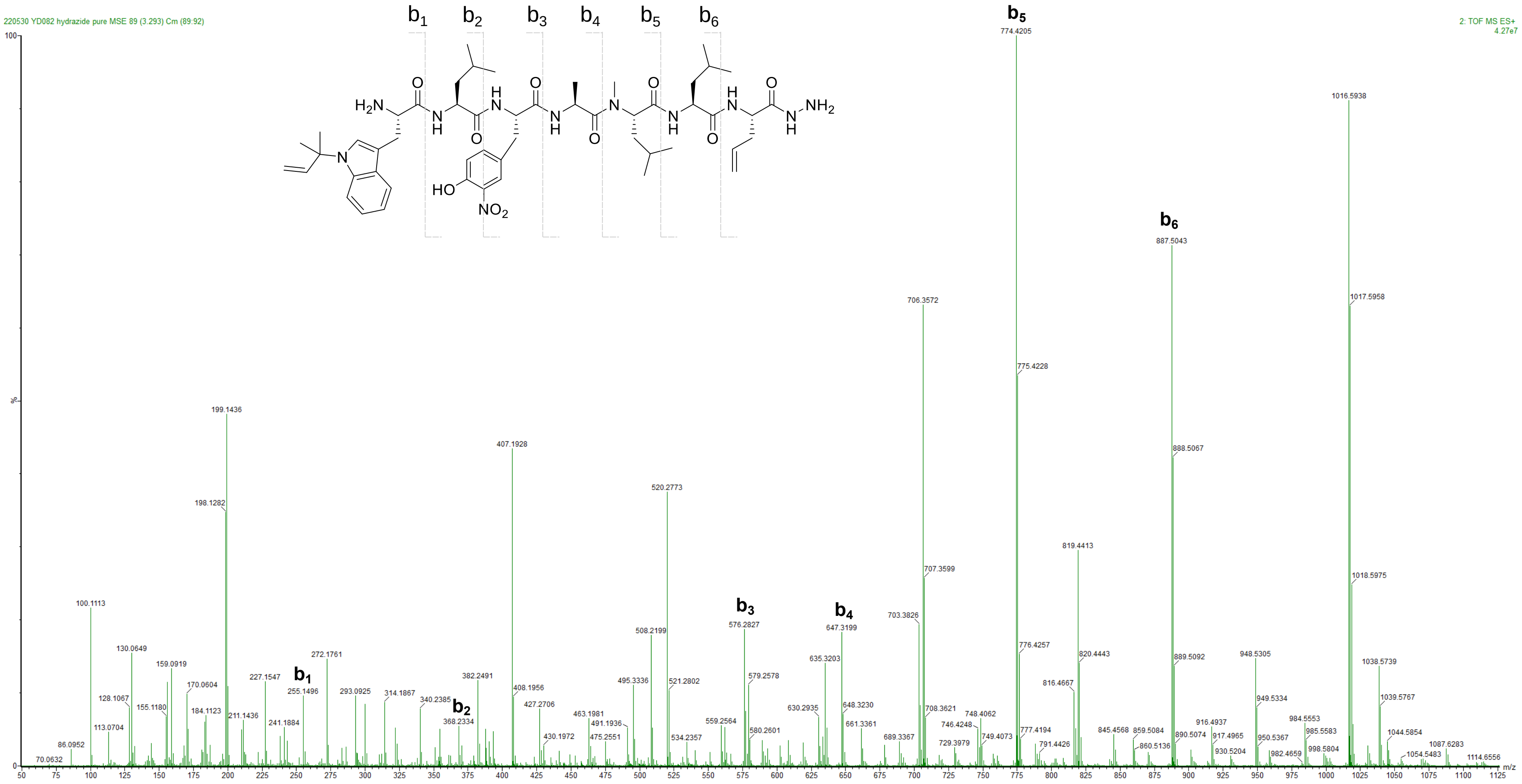

*HRMS spectrum for peptide **8b**: simulated spectrum (top), measured spectrum (below).*

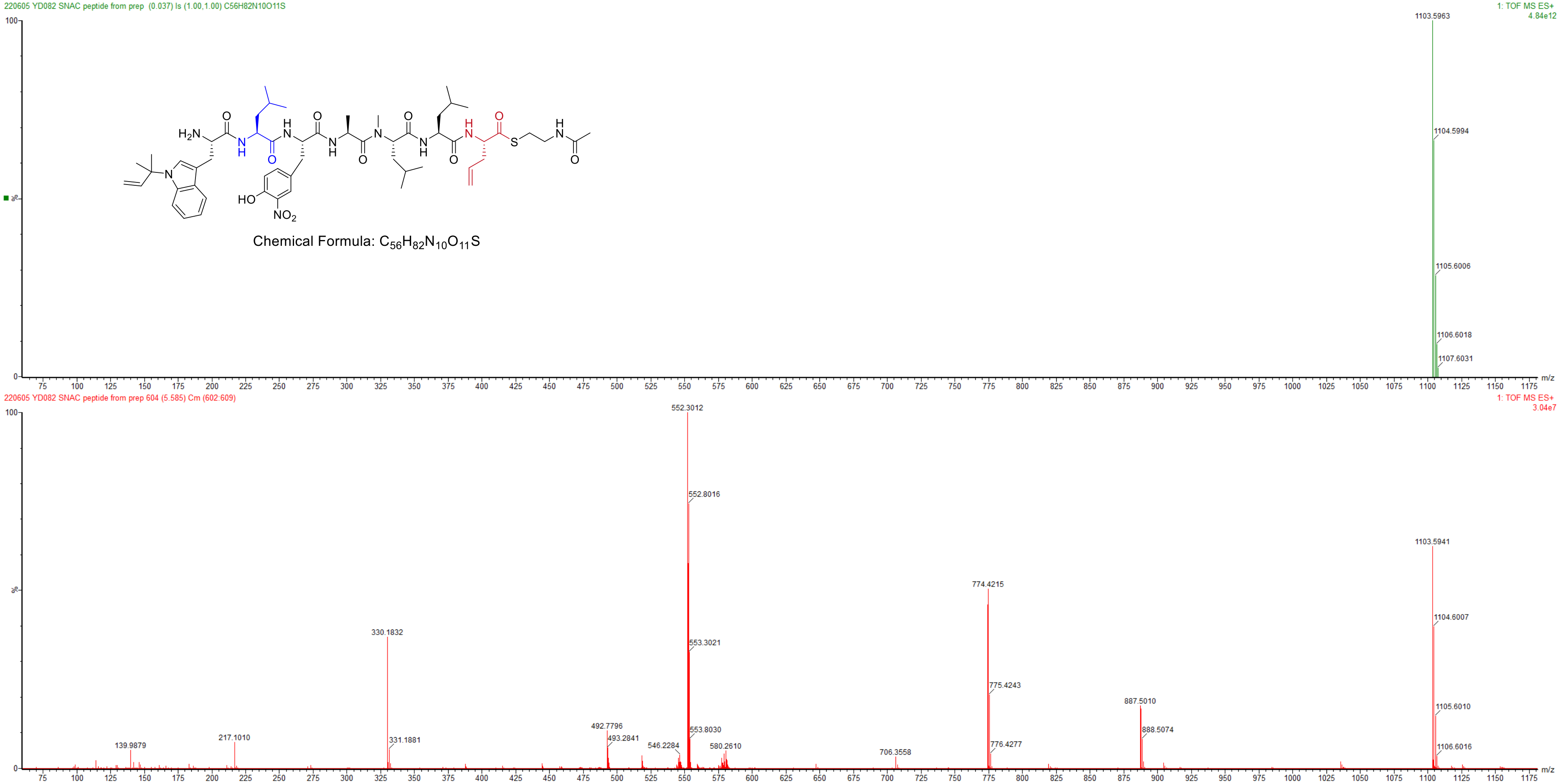

*MS^E spectrum for peptide **8b***

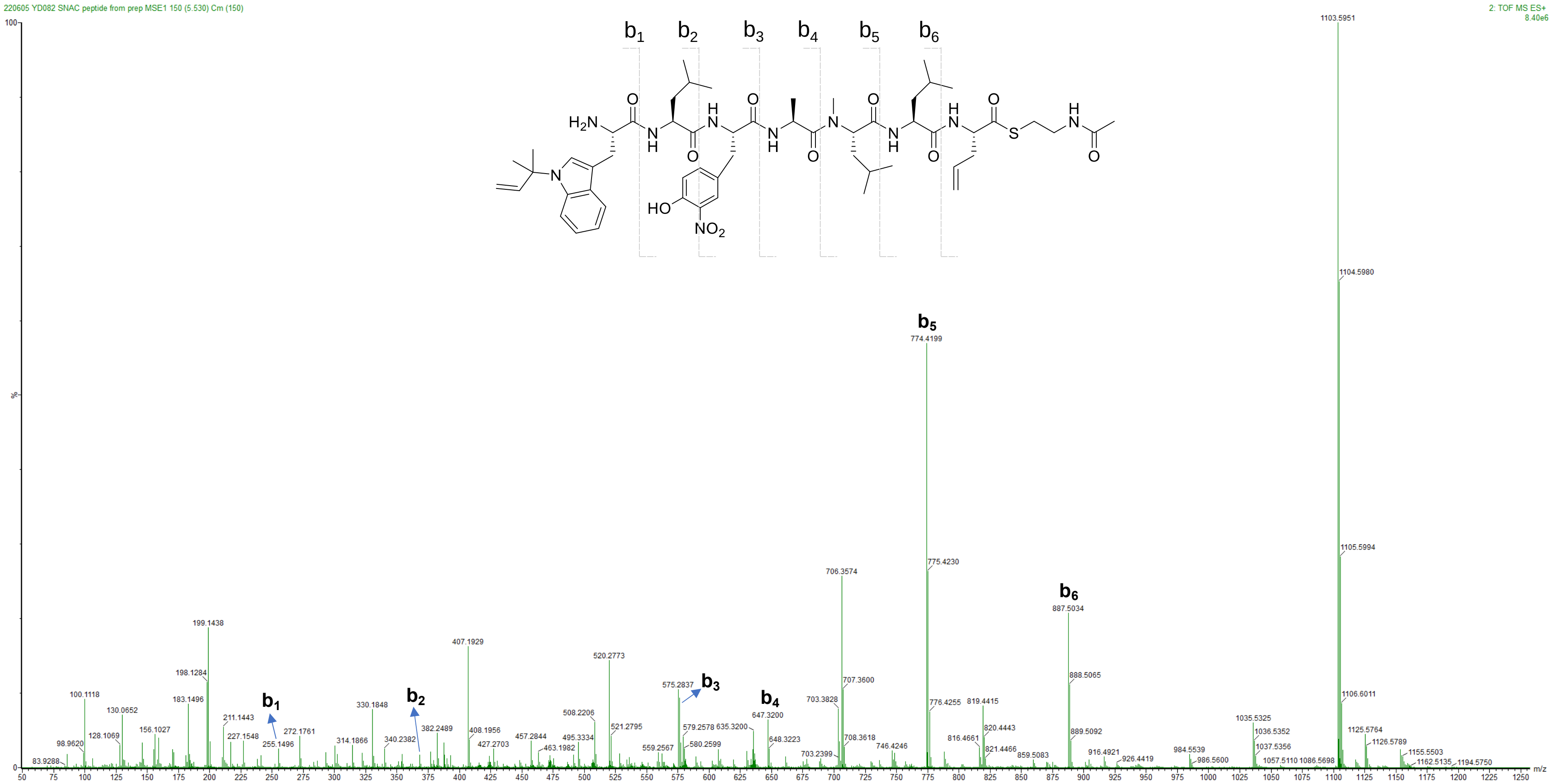

*HRMS spectrum for peptide **2f**: simulated spectrum (top), measured spectrum (below).*

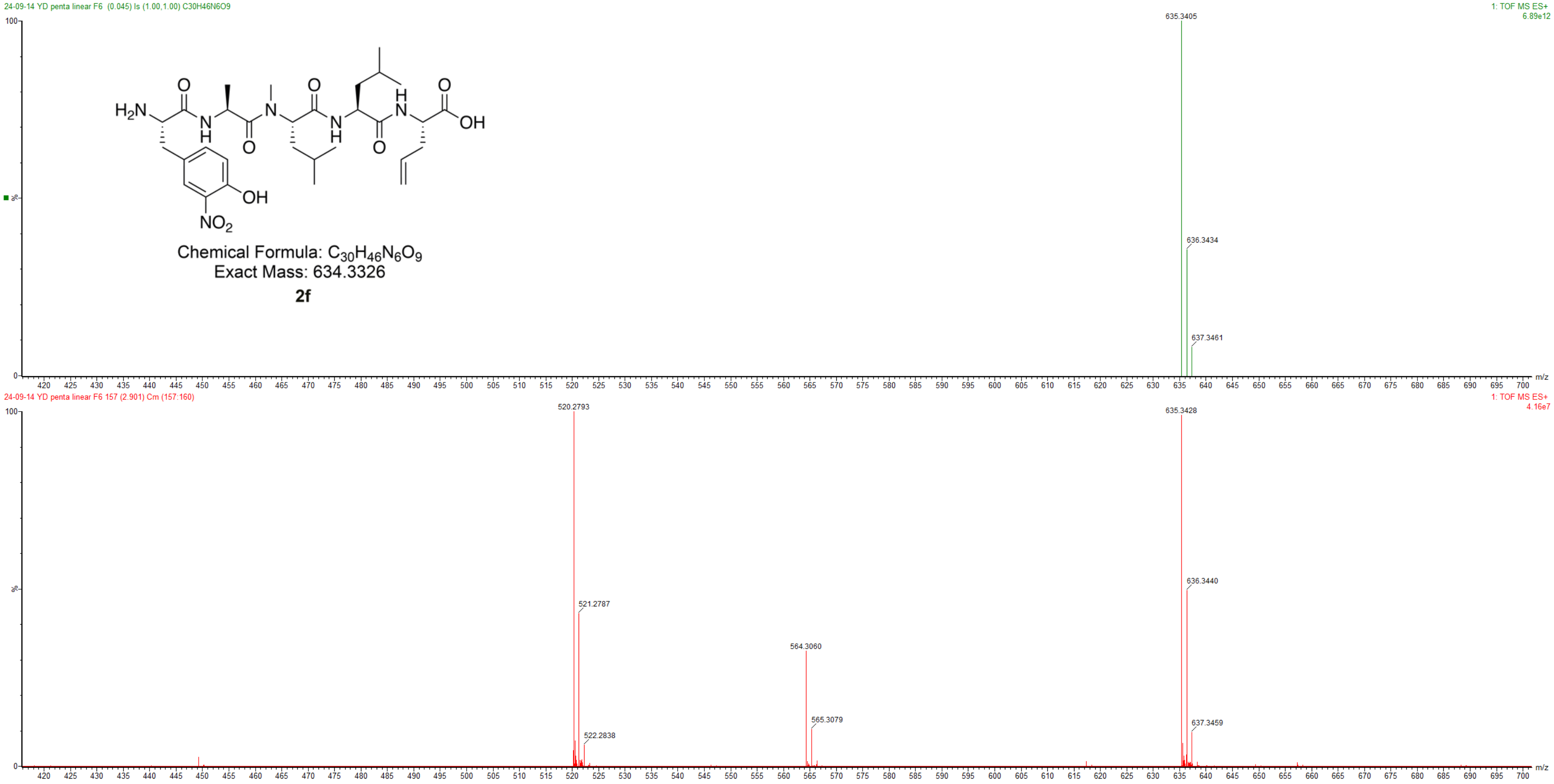

Table 5. DNA and Protein sequence of RufT TE and RufT PCP-TE

	DNA sequence	***Protein sequence***
RufT TE	ATGGGCAGCAGCCATCATCATCATCATCACAGCAGCGGCATGTC<br>GGACTCAGAAGTCAATCAAGAAGCTAAGCCAGAGGTCAAGCCA<br>GAAGTCAAGCCTGAGACTCACATCAATTTAAAGGTGTCCGATGG<br>ATCTTCAGAGATCTTCTTCAAGATCAAAAAGACCACTCCTTTAAG<br>AAGGCTGATGGAAGCGTTCGCTAAAAGACAGGGTAAGGAAATG<br>GACTCCTTAAGATTCTTGTACGACGGTATTAGAATTCAAGCTGAT<br>CAGACCCCTGAAGATTTGGACATGGAGGATAACGATATTATTGA<br>GGCTCACAGAGAACAGATTGGTGGT*GCGAGCTCCAGGGGACCG*<br>*TCCAGTGCCTTCGAGGTGCTGCTGCCGCTGCGCACCAAGGGCG*<br>*ACCGGGCGCCCCTGTTCTGTCTGCACTCGGGCGGCGGGATGA*<br>*GCTGGAATTACGCGAGCCTGCTGCCGCACATCGGCGCCGACAT*<br>*CCCTGTATACGGCCTCCAGGCGCGTGGCCTGTCGGATCCGGAC*<br>*GACCTGCCGGGGTCGGTCGAGGAGGTGGCCGACGACTGCATC*<br>*GAGGCGATGGTCCGGGTGCAGCCCGAAGGGCCGTACCGGCTG*<br>*ATGGGGCACTCCTTCGGGGGCATCGTCGCCCACGCGGTGGCG*<br>*GCCCGGCTGGCCGAGCGCGGTCAGCAGGTCGAACTGATCGTC*<br>*TGCCTCGACGCGAAGCCCGCCGAGGACGAGGAGGACATCCCC*<br>*GAGCACGGGCACGAGGAGTACTACCGGGGGATCCTCGAACTG*<br>*CTGGGCGTGAGCACCGCCGAACTACCGGTGGAGGACCTGACG*<br>*TTCGAGGATTTCGCAGCGGTGGCCCGGACGACCAACACCGTCC*<br>*TCGGCAGCATCGAGGAGAGCGAGTTCCTCACCGTCATGCGGGT*<br>*GATGGAGAACAACATCGAGATCACCAAGGGCTATCGGCACCGA*<br>*CAGGTCGCAACCGAGATGATGCTGTTCGCGGCCACGCAGGAGA*<br>*CCGACACCGTCCTGGAGCCGGATGTGTGGCACGATTACCTTGC*<br>*CGGGCCACTGGAGTACCGGCGCATGGACTGCTCCCACGCCGG*<br>*CATGCTCAAGCCGGAGGTGCTGAGCCAGATCGGCACGTTGATC*<br>*CAGGACCGCCTTAGACGGGGACCAGCGGGTTCTCGG*TAA	MGSSHHHHHHSSGMSDSEVNQEAKPEVKPEVKPETHINLKVSDG<br>SSEIFFKIKKTTPLRRLMEAFAKRQGKEMDSLRFLYDGIRIQADQTP<br>EDLDMEDNDIIEAHREQIGG*ASSRGPSSAFEVLLPLRTKGDRAPLF*<br>*CLHSGGGMSWNYASLLPHIGADIPVYGLQARGLSDPDDLPGSVEE*<br>*VADDCIEAMVRVQPEGPYRLMGHSFGGIVAHAVAARLAERGQQVE*<br>*LIVCLDAKPAEDEEDIPEHGHEEYYRGILELLGVSTAELPVEDLTFED*<br>*FAAVARTTNTVLGSIEESEFLTVMRVMENNIEITKGYRHRQVATEM*<br>*MLFAATQETDTVLEPDVWHDYLAGPLEYRRMDCSHAGMLKPEVLS*<br>*QIGTLIQDRLRRGPAGSR*
RufT PCP-TE	ATGCATCATCACCATCACCATGGTAAGCCTATCCCTAACCCTCT<br>CCTCGGTCTCGATTCTACGGAAAACCTGTATTTTCAGGGAATTG<br>ATCCCTTCACC*GGTGGTCGTGCAGGTCGTCCGCCTCGTACACC*<br>*GCAAGAAGAAGTTCTTTGTACCCTGTTTGCAGAAGTTCTGGGTC*<br>*TGCCTGGTGTTGGTGTTGATGATGGTTTCTTTGATTTAGGTGGT*<br>*CATAGCCTGCTGGCAACCGGTCTGATTAGCCGTATTCAGACCGT*<br>*TCTGGGTGTTGATCTGCCGCTGCGTATTCTGTTTGAAGCAAGCA*<br>*CCGTTGCAGAACTGGCACAGCGTCTGGATCATGATGTTGATGAA*<br>*GGTGCCGGTCGTAGCGCGAGCTCCAGGGGACCGTCCAGTGCC*	MHHHHHHGKPIPNPLLGLDSTENLYFQGIDPFT*GGRAGRPPRTPQ*<br>*EEVLCTLFAEVLGLPGVGVDDGFFDLGGHSLLATGLISRIQTVLGVD*<br>*LPLRILFEASTVAELAQRLDHDVDEGAGRSASSRGPSSAFEVLLPL*<br>*RTKGDRAPLFCLHSGGGMSWNYASLLPHIGADIPVYGLQARGLSD*<br>*PDDLPGSVEEVADDCIEAMVRVQPEGPYRLMGHSFGGIVAHAVAA*<br>*RLAERGQQVELIVCLDAKPAEDEEDIPEHGHEEYYRGILELLGVSTA*<br>*ELPVEDLTFEDFAAVARTTNTVLGSIEESEFLTVMRVMENNIEITKG*<br>*YRHRQVATEMMLFAATQETDTVLEPDVWHDYLAGPLEYRRMDCS*<br>*HAGMLKPEVLSQIGTLIQDRLRRGPAGSR*

	*TTCGAGGTGCTGCTGCCGCTGCGCACCAAGGGCGACCGGGCG CCCCTGTTCTGTCTGCACTCGGGCGGCGGGATGAGCTGGAATT ACGCGAGCCTGCTGCCGCACATCGGCGCCGACATCCCTGTATA CGGCCTCCAGGCGCGTGGCCTGTCGGATCCGGACGACCTGCC GGGGTCGGTCGAGGAGGTGGCCGACGACTGCATCGAGGCGAT GGTCCGGGTGCAGCCCGAAGGGCCGTACCGGCTGATGGGGCA CTCCTTCGGGGGCATCGTCGCCCACGCGGTGGCGGCCCGGCT GGCCGAGCGCGGTCAGCAGGTCGAACTGATCGTCTGCCTCGAC GCGAAGCCCGCCGAGGACGAGGAGGACATCCCCGAGCACGGG CACGAGGAGTACTACCGGGGGATCCTCGAACTGCTGGGCGTGA GCACCGCCGAACTACCGGTGGAGGACCTGACGTTCGAGGATTT CGCAGCGGTGGCCCGGACGACCAACACCGTCCTCGGCAGCAT CGAGGAGAGCGAGTTCCTCACCGTCATGCGGGTGATGGAGAAC AACATCGAGATCACCAAGGGCTATCGGCACCGACAGGTCGCAA CCGAGATGATGCTGTTCGCGGCCACGCAGGAGACCGACACCGT CCTGGAGCCGGATGTGTGGCACGATTACCTTGCCGGGCCACTG GAGTACCGGCGCATGGACTGCTCCCACGCCGGCATGCTCAAGC CGGAGGTGCTGAGCCAGATCGGCACGTTGATCCAGGACCGCCT GCGGCGCGGACCCGCCGGTTCCCGG*TAG	

4.3 High Resolution UPLC-HRMS analysis of His$_6$-SUMO-RufT TE and RufT PCP-TE domain.

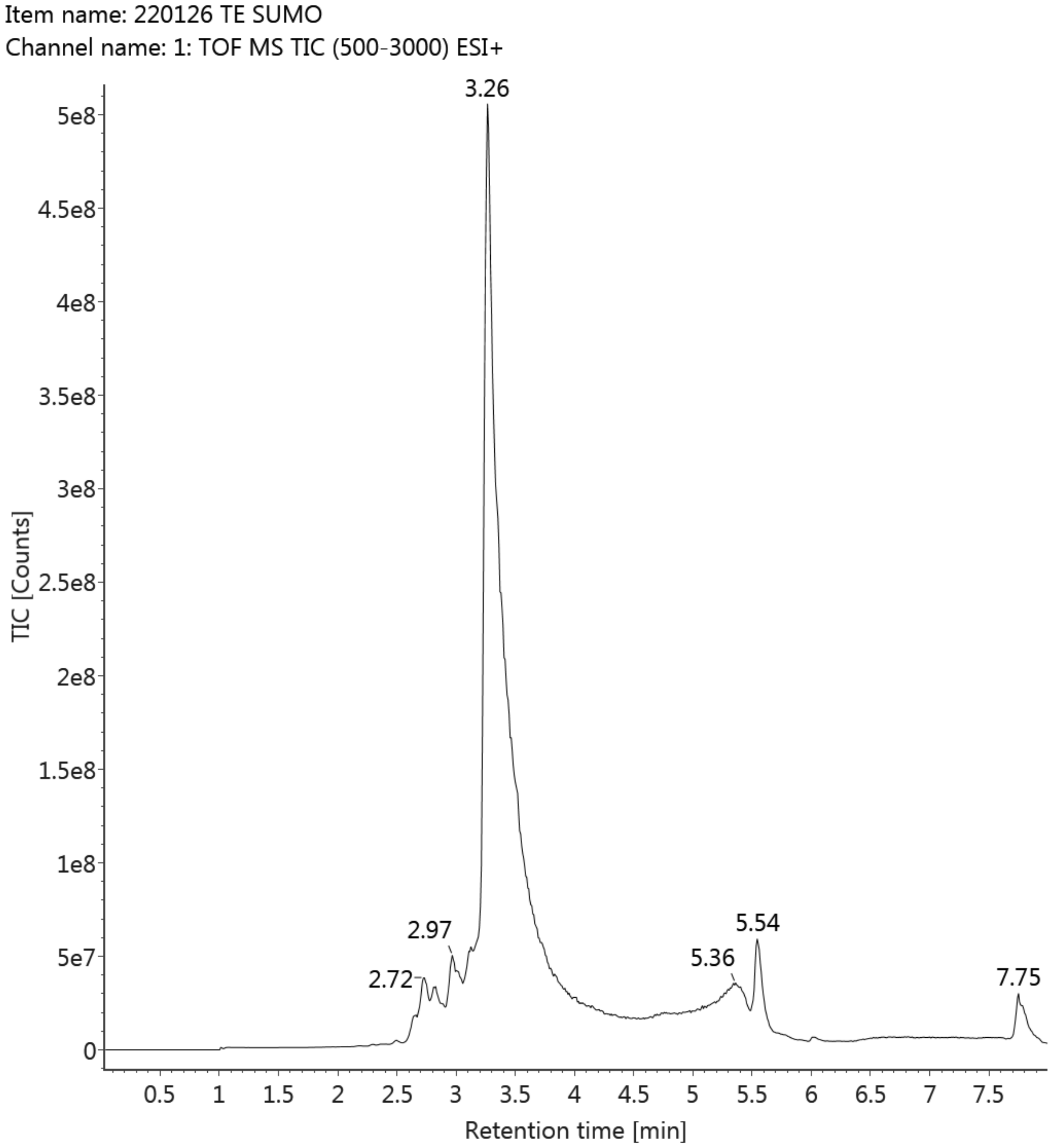

High Resolution Mass Spectrum of protein His$_6$-SUMO-RufT TE (cal. Average mass 42516.80 Da) and (inset) deconvoluted spectrum .

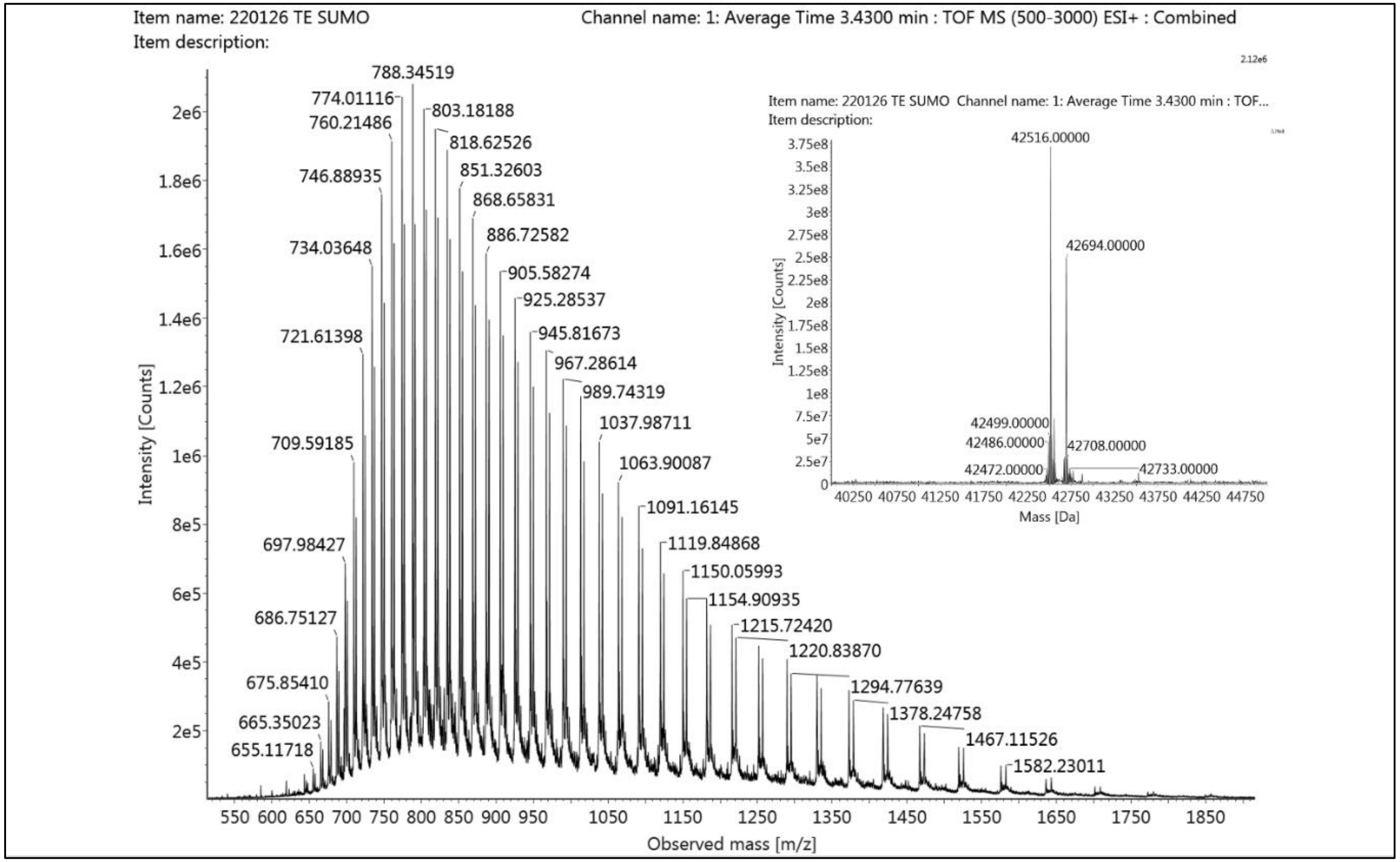

Note: 42,694 m/z corresponds to M+178 Da, which is likely phosphogluconoylation of the His$_6$ Tag during recombinant protein production in *E. coli*.[6]

High Resolution Mass Spectrum of protein RufT PCP-TE (cal. Average mass 43090.68 Da) (deconvoluted spectrum).

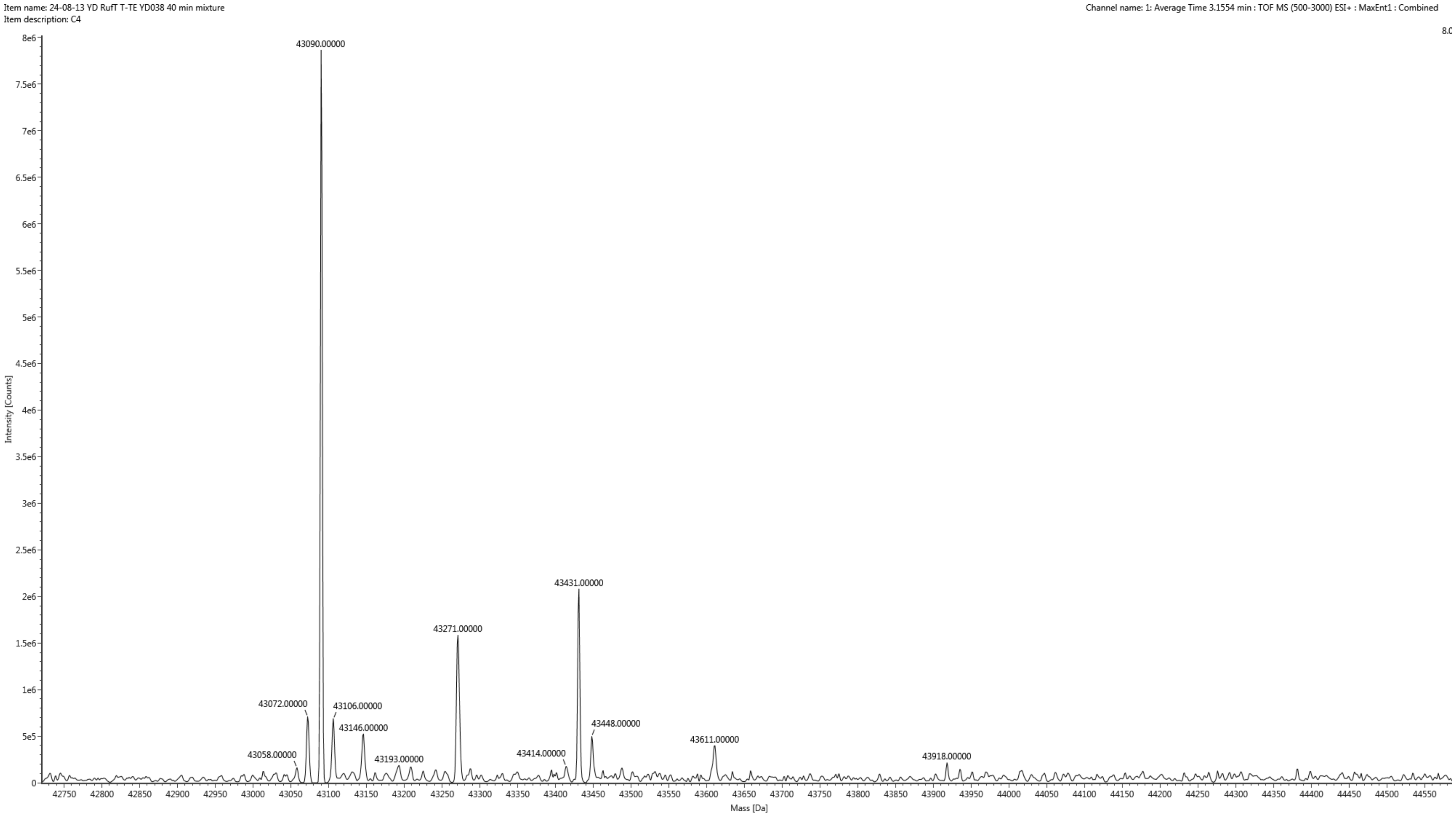

5. Supplementary References

1. Ding, Y. *et al.* Rapid Peptide Cyclization Inspired by the Modular Logic of Nonribosomal Peptide Synthetases. *J. Am. Chem. Soc.* **146**, 16787–16801 (2024).
2. Choules, M. P. *et al.* Residual Complexity Does Impact Organic Chemistry and Drug Discovery: The Case of Rufomyazine and Rufomycin. *J. Org. Chem.* **83**, 6664–6672 (2018).
3. Bachmann, B. O. & Ravel, J. Chapter 8 Methods for In Silico Prediction of Microbial Polyketide and Nonribosomal Peptide Biosynthetic Pathways from DNA Sequence Data. in *Methods in Enzymology* vol. 458 181–217 (Elsevier, 2009).
4. Frueh, D. P. *et al.* Dynamic thiolation–thioesterase structure of a non-ribosomal peptide synthetase. *Nature* **454**, 903–906 (2008).
5. Tomita, H., Katsuyama, Y., Minami, H. & Ohnishi, Y. Identification and characterization of a bacterial cytochrome P450 monooxygenase catalyzing the 3-nitration of tyrosine in rufomycin biosynthesis. *J. Biol. Chem.* **292**, 15859–15869 (2017).
6. Geoghegan, K. F. *et al.* Spontaneous alpha-N-6-Phosphogluconoylation of a “His Tag” in *Escherichia coli*: The Cause of Extra Mass of 258 or 178 Da in Fusion Proteins. *Anal Biochem.* **267**,169-84 (1999).
